# Antioxidant Activity and Phenolic Compound Identification and Quantification in Western Australian Honeys

**DOI:** 10.3390/antiox12010189

**Published:** 2023-01-12

**Authors:** Ivan Lozada Lawag, Md Khairul Islam, Tomislav Sostaric, Lee Yong Lim, Katherine Hammer, Cornelia Locher

**Affiliations:** 1 Cooperative Research Centre for Honey Bee Products Limited (CRC HBP), The University of Western Australia, Agriculture North M085, Crawley, WA 6009, Australia; 2 Division of Pharmacy, School of Allied Health, The University of Western Australia, Curnow Building M315, Crawley, WA 6009, Australia; 3 School of Biomedical Sciences, The University of Western Australia, L Block QEII Medical Centre, Monash Ave., Crawley, WA 6009, Australia

**Keywords:** HPTLC, HPTLC-DPPH, HPTLC-derived database, DPPH, FRAP, TPC, Folin–Ciocalteu assay, phenolics, *Eucalyptus marginata* (Jarrah), *Corymbia calophylla* (Marri), *Calothamnus* spp. (Red Bell), *Agonis flexuosa* (Coastal Peppermint), honey, colour hue

## Abstract

This study reports on the total phenolic content and antioxidant activity as well as the phenolic compounds that are present in *Calothamnus* spp. (Red Bell), *Agonis flexuosa* (Coastal Peppermint), *Corymbia calophylla* (Marri) and *Eucalyptus marginata* (Jarrah) honeys from Western Australia. The honey’s total phenolic content (TPC) was determined using a modified Folin–Ciocalteu assay, while their total antioxidant activity was determined using FRAP and DPPH assays. Phenolic constituents were identified using a High Performance Thin-Layer Chromatography (HTPLC)-derived phenolic database, and the identified phenolic compounds were quantified using HPTLC. Finally, constituents that contribute to the honeys’ antioxidant activity were identified using a DPPH-HPTLC bioautography assay. Based on the results, *Calothamnus* spp. honey (*n* = 8) was found to contain the highest (59.4 ± 7.91 mg GAE/100 g) TPC, followed by *Eucalyptus marginata* honey (50.58 ± 3.76 mg GAE/100 g), *Agonis flexuosa* honey (36.08 ± 4.2 mg GAE/100 g) and *Corymbia calophylla* honey (29.15 ± 5.46 mg GAE/100 g). In the FRAP assay, *Calothamnus* spp. honey also had the highest activity (9.24 ± 1.68 mmol Fe^2+^/kg), followed by *Eucalyptus marginata* honey (mmol Fe^2+^/kg), whereas *Agonis flexuosa* (5.45 ± 1.64 mmol Fe^2+^/kg) and *Corymbia calophylla* honeys (4.48 ± 0.82 mmol Fe^2+^/kg) had comparable FRAP activity. In the DPPH assay, when the mean values were compared, it was found that *Calothamnus* spp. honey again had the highest activity (3.88 ± 0.96 mmol TE/kg) while the mean DPPH antioxidant activity of *Eucalyptus marginata*, *Agonis flexuosa*, and *Corymbia calophylla* honeys were comparable. Kojic acid and epigallocatechin gallate were found in all honeys, whilst other constituents (e.g., m-coumaric acid, lumichrome, gallic acid, taxifolin, luteolin, epicatechin, hesperitin, eudesmic acid, syringic acid, protocatechuic acid, t-cinnamic acid, o-anisic acid) were only identified in some of the honeys. DPPH-HPTLC bioautography demonstrated that most of the identified compounds possess antioxidant activity, except for t-cinnamic acid, eudesmic acid, o-anisic acid, and lumichrome.

## 1. Introduction

Next to antibacterial activity, the antioxidant activity of honey has attracted considerable interest in recent years because of its association with anti-inflammatory, anti-cancer and also anti-aging effects [1]. Commonly, the determination of the antioxidant activity of honey involves the use of several popular colorimetric assays, such as the measurement of total phenolic content (TPC) [2,3,4,5,6], total flavonoid content (TFC) [5,6,7], free radical scavenging activity using the 2,2-diphenyl-1-picrylhydrazyl (DPPH) assay [4,5,6,7,8,9], or measuring the ferric reducing antioxidant power (FRAP) [2,8,10] which is also known as Trolox equivalent antioxidant capacity (TEAC). High antioxidant potential in these assays is usually observed for samples with high phenolic and flavonoid content [11]. Thus, the variance in antioxidant properties among honeys from different floral and geographical origins is mainly due to the difference in the composition of their polyphenolic fraction.

Although they are present in honey in only small amounts, phenolic compounds are well studied due to their biological activities [1,12] and their influence on honeys’ organoleptic characteristics [1,13,14,15,16,17]. They have also been identified as potential chemical markers for quality assurance and in authenticating the geographical and botanical origin of honeys [18,19,20,21]. For example, kaempferol is seen as a key marker for rosemary honey [22], naringenin, caffeic acid and hesperetin for citrus blossom honey [23,24], safflomin for safflower honey [16], ellagic acid for heather honey, caffeic, p-coumaric and ferulic acids for chestnut honey [25] and quercetin for sunflower honey [21]. Similarly, pinocembrin, pinobanksin and chrysin are not only characteristic flavonoids present in propolis but have also been found in many European honeys [21]. The identification of such marker compounds in honey is, however, challenging because they are present at only low concentrations and their isolation and successful chemical identification is strongly dependent on the respective extraction and analysis methods employed [13,26,27,28].

Based on a comprehensive review of 130 research papers, it was found that 161 phenolic compounds have been identified in honey to date [29], most of which belong to the class of hydroxycinnamic acid derivatives, hydroxybenzoic acid derivatives, flavonols, flavones and flavanones [29]. High-Performance Liquid Chromatography (HPLC) coupled with diode array detection (DAD) appears to be the most commonly employed technique for the qualitative and quantitative analysis of phenolic compounds, followed by Liquid Chromatography-Mass Spectrometry (LC-MS).

Recently, a novel High Performance Thin-Layer Chromatography (HPTLC)-derived database has also been developed and successfully used for the identification of phenolic compounds in Manuka honey [30]. An important advantage of HPTLC over other chromatographic techniques is that it can be paired with post-chromatographic derivatization, even with biochemical reagents. An example is HPTLC-DPPH analysis, which allows visualization and quantification of the antioxidant activity of individual compounds in the chromatographically separated mixture [31]. This method has already been employed in honey analysis and has been demonstrated to be useful in visualizing honey constituents with antioxidant properties [32,33,34].

Western Australia (WA) is home to 8 of Australia’s 15 biodiversity hotspots, which are characterised by a high percentage of endemic flora, (https://www.dcceew.gov.au/science-research/australias-biological-resources/access-resources/wa, accessed on 22 December 2022) therefore, most of the plants foraged by bees (*Apis mellifera*) are unique only to the State. Based on unpublished data by the Cooperative Research Centre for Honey Bee Products (a program established and supported under the Australian Government’s Cooperative Research Centres Program), honey samples collected (437 samples) across Western Australia were mostly monofloral in nature and belonged to 48 different botanical species from 10 different families, a majority of which belonged to Myrtaceae (34) and Proteaceae (7) families. Myrtaceae include the genus *Eucalyptus* which represents the most abundant type of trees in the State, along with trees and shrubs of the genus *Melaleuca*, and shrubs of the genus *Calothamnus.* Myrtaceae is the most important plant family foraged by bees. Proteaceae is another important honey producing family and includes trees and shrubs from the genus *Banksia* and *Grevilla* [35]. Table S1 summarises the identity, botanical origin, and families of the honeys collected in WA. Among the honeys produced in the state, honeys from *Eucalyptus marginata* (Jarrah, Myrtaceae), *Corymbia calophylla* (Marri, Myrtaceae), and *Agonis flexuosa* (Coastal Peppermint, Myrtaceae) and a shrub honey harvested from *Calothamnus* spp. (Red Bell, Myrtaceae) are considered iconic and are popular amongst consumers.

The level of information available on the chemical composition and bioactivity of various honeys of different floral origin varies considerably. While some, such as New Zealand Manuka honey derived from *Leptospermum scoparium*, have attracted considerable academic and commercial interests [36], research data on other honeys is scant. This is undoubtedly the case for honeys derived from Western Australian (WA) floral sources. This presents a significant gap in current knowledge that is addressed in this study. For Western Australian monofloral honeys such as *Eucalyptus marginata* (Jarrah), *Corymbia calophylla* (Marri), *Calothamnus* spp. (Red Bell) and *Agonis flexuosa* (Coastal Peppermint), very few studies have yet focused on their antioxidant properties [37,38] and the constituents that contribute to this activity. For example, to date, only some phenolic constituents have been reported for Jarrah honey [39]. To address these knowledge gaps, the aims of this study were to determine the total phenolic content and the total antioxidant activity of the said Western Australian honeys using a modified Folin–Ciocalteu as well as FRAP and DPPH assays, to identify and quantify phenolic constituents in these honeys using a HPTLC-derived phenolic database and to determine the contribution of various constituents to the overall antioxidant activity of the honeys using an HPTLC-DPPH assay. The findings of this research will assist WA’s honey industry in selecting appropriate floral sources that might lead to high-value products.

## 2. Materials and Methods

### 2.1. Chemicals and Reagents

The chemicals and reagents used in this study were sourced as follows: Folin and Ciocalteu’s phenol reagent 2N, (F9252-1L), 2,4,6-tris(2-pyridyl)-1,3,5-triazine (TPTZ, 3682-35-7), iron (III) chloride hexahydrate (10025-77-1), iron (II) sulphate heptahydrate (7782-63-0), trolox (53188-07-1), fructose (57-48-7), and maltose (6363-53-7) from Sigma Aldrich Truganina, Australia; vanillin (121-33-5) from Sigma-Aldrich, St. Louis, MO, USA; anhydrous magnesium sulfate (7487-88-9), anhydrous sodium carbonate (497-19-8), aminoethyl diphenylborinate (524-95-8), glucose (50-99-7) sucrose (57-50-1), ethanol (64-17-5), from Chem Supply, Port Adelaide, South Australia, Australia; toluene from APS Chemicals, Sydney, New South Wales, Australia; naringenin (98%, 67604-48-2) from Alfa Aesar, Heysham, Lancashire, UK; anhydrous sodium acetate (127-09-3), glacial acetic acid (64-19-7), ethyl acetate (141-78-6), and formic acid (64-18-6) from Ajax Finechem, Wollongong, New South Wales, Australia; hydrochloric acid (7647-01-0) from Asia Pacific Specialty Chemicals Limited, Seven Hills, New South Wales, Australia; 2,2-diphenyl-1-picrylhydrazyl (DPPH, 1898-66-4) from Fluka AG, Buchs, St. Gallen, Switzerland; 3,4,5-trihydroxybenzoic acid (149-91-7) from Ajax Chemicals Ltd. Sydney, New South Wales, Australia; dichloromethane (75-09-2), acetonitrile (75-05-8), concentrated sulfuric acid (7664-93-9) and HPTLC Silica gel 60 F254 Plates 10 × 20 cm from Merck KGaA, Darmstadt, Hesse, Germany; PEG (25322-68-3) from PharmAust Manufacturing, Welshpool, Western Australia, Australia; Methanol (CH_3_OH, B.n. 19758725, 67-56-1) from Scharlau, Barcelona, Catalonia, Spain.

Phenolic compounds and other standards that were included in the HPTLC database were chosen based on an extensive review of phenolic compounds reported in honey [29] and were purchased from Ajax Finechem Pvt. Ltd., (Sydney, New South Wales, Australia), AK Scientific, Inc. (Union City, CA, USA), Alfa Aesar (Heysham, Lancashire, UK), Angene International Ltd. (Nanjing, China), Chem Supply Australia Pty Ltd. (Port Adelaide, Australia), Combi-Blocks Inc., (San Diego, CA, USA), Wuhan ChemFaces Biochemical Co., Ltd. (Wuhan, China), Sigma Aldrich (Castle Hill, Australia), and Sigma-Aldrich (St. Louis, MO, USA) [30].

### 2.2. Honey Samples

**Figure 1:**
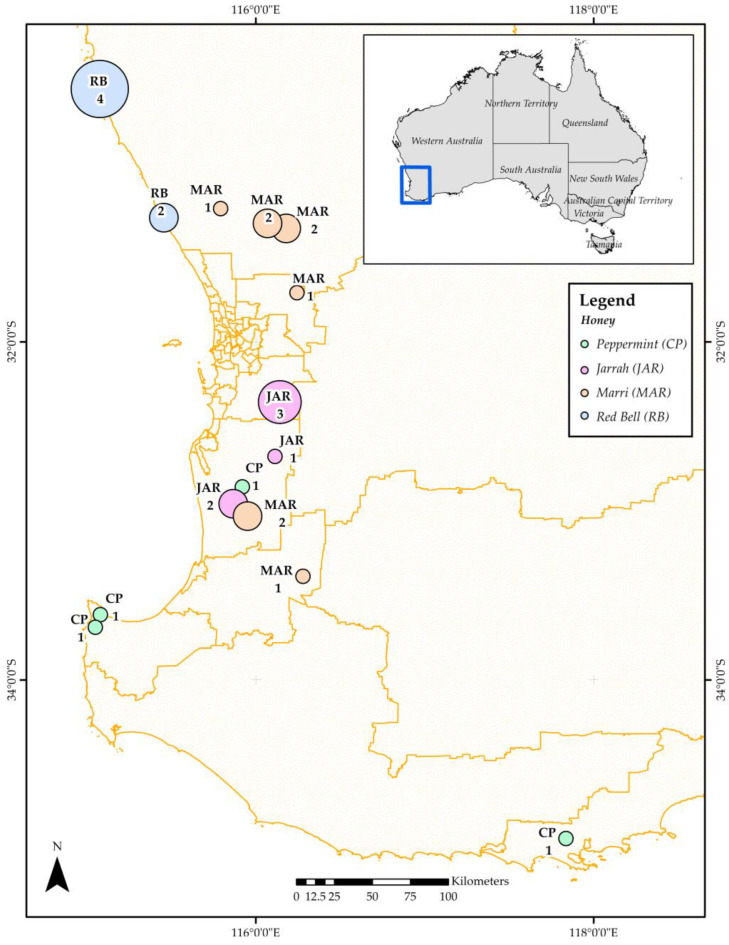
Collection sites of honeys used in this study (samples shown for which this information was available). Map generated using ARCGIS Version 10.8. Redlands, CA, USA (Note: Numbers correspond to the number of samples of honey collected from each specific location; Source: https://www.abs.gov.au/statistics/standards/australian-statistical-geography-standard-asgs-edition-3/jul2021-jun2026/access-and-downloads/digital-boundary-files, accessed on 10 November 2022).

*Calothamnus* spp. honey (Red Bell, Myrtaceae, *n* = 8), *Agonis flexuosa* honey (Coastal Peppermint, Myrtaceae, *n* = 5), *Corymbia calophylla* honey (Marri, Myrtaceae, *n* = 13), and *Eucalyptus marginata* honey (Jarrah, Myrtaceae, *n* = 6) were purchased from different suppliers in Western Australia (WA) (see Table S1). Figure 1 shows the geographical locations from where the honeys were collected.

Individual honey samples were authenticated based on their HPTLC fingerprints following established protocols (see Figures S1–S4) [37,38,40] and based on this authentication, a pooled sample for each honey was prepared by mixing equal amounts of each individual sample from the same floral source. It was deemed that such a pooled sample would better reflect the typical chemical composition of a honey rather than analysing an individual honey with a chemical profile that specifically mirrors its unique location, time of collection and processing [40]. Therefore, the pooled honey samples were used in this study for constituent identification and quantification.

An artificial honey solution was prepared by mixing 21.625 g of fructose, 18.125 g of glucose, 1.000 g of maltose, 0.750 g of sucrose and 8.500 g of water [41].

### 2.3. Preparation of Honey Samples

For the total phenolic content analysis and antioxidant analyses, individual honeys were prepared in triplicates as 20% *w*/*v* aqueous solutions while for phenolic identification and quantification experiments, pooled honeys were extracted using an organic solvent. The extraction process involved adding 1 g of each pooled honey sample to 2 mL deionised water in stoppered glass test tubes followed by vortex mixing. The resulting solution was then extracted three times with 5 mL dichloromethane and acetonitrile (1:1, *v*/*v*). The combined organic extracts were dried using anhydrous MgSO_4_, filtered, and evaporated to dryness using a heating block (Stuart SBHCONC/1 Sample Concentrator) set at 35 °C. The organic honey extracts were stored at 4 °C until analysis for which they were reconstituted with 100 µL methanol.

### 2.4. Determination of Total Phenolic Content (TPC)

The TPC assay was performed based on the methodology described by Liberato et al. with minor modifications [42]. This protocol has previously been employed in the analysis of the TPC of some Western Australian bee products [37,38,41,43].

In brief, 200 µL of aqueous honey solution (20%, *w*/*v*) or 100 µL of gallic acid standards (0.06 mg/mL to 0.18 mg/mL) spiked with 100 µL of artificial honey solution (40%, *w*/*v*) [39] were reacted with 1 mL of diluted Folin–Ciocalteu reagent (1 mL of Folin–Ciocalteu reagent in 30 mL deionised water). After 5 min, 800 µL of 0.75% Na_2_CO_3_ was added and allowed to react for 2 h, excluded from light. Sample absorbance at 760 nm was then measured (Carry 60 Bio UV–Vis spectrophotometer) using 100 µL of deionised water spiked with 100 µL of artificial honey solution (40%, *w*/*v*), 1 mL of Folin–Ciocalteu reagent and 800 µL of 0.75% Na_2_CO_3_ as a blank. The analysis was carried out in triplicate and the mean result for each sample was expressed as mg gallic acid equivalent (GAE) per 100 g of honey.

(1) $$TPC\;Value\;of\;Sample\;\left(mg\;Gallic\;Acid\;\right)=\frac{\left(\Delta Abs-intercept\right)}{slope}\;$$

### 2.5. Determination of Antioxidant Activity Using the Ferric Reducing Antioxidant Power (FRAP) Assay

The FRAP assay, which is based on the reduction of ferric 2,4,6-tris(2-pyridyl)-1,3,5-triazine [Fe(III)-TPTZ] to ferrous complex at low pH followed by a spectrophotometric analysis, was performed according to the protocol described by Almeida et al. [44] with minor modifications. This protocol has previously been used in our laboratory to determine the FRAP activity of various bee products [37,38,41,43].

In brief, a 1:1:10 (*v*/*v*/*v*) ratio of the FRAP reagent was prepared by mixing 10 mM TPTZ (dissolved in 40 mM HCl), 20 mM aqueous FeCl_3_·6H_2_O and 300 mM aqueous acetate buffer (pH 3.6). The reagent mixture was freshly prepared prior to each experiment and incubated at 37 °C prior to use. Ferrous sulphate (FeSO_4_·7H_2_O) standards ranging from 200 µM to 1200 µM, along with the standard concentration of 600 µM which was used as a positive control, were freshly prepared prior to each experiment and stored on ice.

A total of 20 µL of honey solution or standards were mixed with 180 µL of FRAP reagent in a 96-well microplate (Greiner Bio-One 96-well Microplate Flat Bottom), and the absorbance of the reaction mixture after 30 min of incubation at 37 °C was determined at 620 nm (BMG Labtech POLARstar Optima Microplate Reader). The FRAP antioxidant activity was determined based on the interpolation of the standard curve and expressed as mmol Fe^2+^ equivalent (FE)/kg of honey (mean of triplicate results).

(2) $$FRAP\;Value\;of\;Sample\;\left(µM\;Fe\;\left(II\right)\right)=\frac{\left(\Delta Abs-intercept\right)}{slope}$$

### 2.6. Determination of Antioxidant Activity Using the 2,2-Diphenyl-1-Picrylhydrazyl (DPPH) Radical Scavenging Assay

The DPPH assay in this study was based on the protocol described by Karabagias et al. [45] with minor modifications [37,38,41,43]. The radical 2,2-diphenyl-1-picrylhydrazyl (DPPH) is purple in colour and decays to yellow in the presence of antioxidants. The resulting change can be captured at 520 nm. The DPPH reagent mixture was prepared using a ratio of 19:10 (*v*/*v*) of 0.130 mM methanolic DPPH solution and 100 mM pH 5.5 aqueous NaC_2_H_3_O_2_ buffer. Aqueous Trolox solutions with concentrations ranging from 100–600 µM (pH adjusted to pH 7.0) were used to derive the calibration curve, with the 400 µM standard also serving as a positive control.

A total of 10 µL of aqueous honey solution or standards were placed in a 96-well microplate, followed by 290 µL of DPPH reagent, and then mixed. The reaction mixture was kept in the dark and the absorbance was measured at 520 nm after 120 min using a microplate reader (Greiner Bio-One 96-well Microplate Flat Bottom). The mean radical scavenging activity of triplicate samples of honey solutions or standards was expressed as Trolox Equivalent (TE), calculated based on the interpolation of the standard curve, and for the honey samples then also expressed as µmol Trolox equivalent per kg of honey.

(3) $$DPPH\;Value\;of\;Sample\;\left(µM\;Trolox\right)=\frac{\left(\Delta Abs-intercept\right)}{slope}$$

### 2.7. Phenolic Constituent Identification in Honey

The identification of phenolic honey constituents was performed using a validated HPTLC based database of phenolic compounds. In brief, honeys were first fingerprinted using HPTLC under various conditions and the resulting data (i.e., Rf values, colour hues, UV-Vis and fluorescence λmax and λmin prior to derivatisation, UV-Vis and fluorescence λmax after derivatisation) were matched with standards included in the database [30]. Potential matches were confirmed by spectral overlay analysis [30].

In this study, as additional confirmation of correct identification, a mixture of the identified compounds (7.4 µL, for concentrations see Table 1) in each honey was used to over-spot the respective neat honey extract (7 µL). A corresponding increase in the absorbance of the respective honey extract bands was seen as confirmation of the correct identification.

The CAMAG HPTLC system (Muttenz, Switzerland) used in this study consisted of a CAMAG TLC visualizer 2, Linomat V semi-automatic sample applicator, and ADC2 automated development chamber, a TLC scanner IV, a derivatiser, and a TLC plate heater III. The system was operated by VisionCATS Version 3.1 software, which controls all chromatographic operations and analyses.

In order to perform the phenolic compound identification, honey extracts were subjected to the same HPTLC conditions used to establish the database (Table 2) using two solvent systems: (a) MPA, consisting of toluene: ethyl acetate: formic acid (2:8:1, *v*/*v*/*v*), and (b) MPB, consisting of toluene: ethyl acetate: formic acid (6:5:1, *v*/*v*/*v*) [44,45], as well as two different derivatising reagents, natural product-polyethylene glycol reagent (NP-PEG) and vanillin-sulfuric acid reagent (VSA).

Naringenin (0.5 mg/mL in methanol), with an application volume of 4 µL, was used as HPTLC reference standard, and for all honey extracts a volume of 7 µL was used. All samples were applied as 8 mm bands, 8 mm from the bottom of the HPTLC plate at a rate of 150 nLs^−1^ (aided by liquid nitrogen at a pressure of 10,000 mmHg). The chromatographic separation was performed on 20 × 10 cm HPTLC plates (glass-backed silica gel 60 F_254_ plates) in an automated twin trough development chamber activated with MgCl_2_·6H_2_O at 33–38% relative humidity. Saturation pads were used to saturate the system for 15 min and plates were preconditioned with the mobile phase for 5 min, and then developed automatically to a distance of 70 mm at room temperature before being automatically dried for 5 min. Photo-documentations under 254 nm, 366 nm, and white light in transmittance mode (T) were performed on the developed plates in order to detect the separated honey constituents. From this information corresponding peak profiles were generated, and major peaks automatically determined by the software.

The scanning of individual major bands in the honey extracts was carried out using the TLC Scanner 4 in both UV-Vis mode (190–900 nm) and fluorescence mode (190–380 nm) with the following settings: Dimension set at 5 × 0.2 mm (micro), optimisation set for maximum resolution, scanning speed 20 nm/s and use of K400 optical filter. Deuterium (190–380 nm) and tungsten (380–900 nm) were used as lamps and the scans in fluorescence excitation mode were set at 380 < /400 nm and the emissions were observed at 190–270 nm. Three spectral scans were performed for each sample, prior to and after derivatisation with each of the derivatisation reagents used.

To perform the derivatisation of the plates with NP-PEG reagent, plates were first sprayed with 3 mL of 1% NP reagent using a green nozzle at level 3 and then allowed to dry for 5 min at 40 °C. The plates were then sprayed again, this time with 5% PEG reagent using a blue nozzle at level 2, dried for 5 min at 40 °C and the resulting image was captured at 366 nm [29]. To derivatise using VSA reagent, plates were sprayed with 3 mL of 1% vanillin sulphuric acid reagent using a yellow nozzle at level 3, and then heated for 3 min at 115 °C for 3 min, and after cooling for 2 min, the plates were visualised at 366 nm and T white light.

A system suitability test (SST) was performed for each plate analysis as a quality control step. This was performed by utilising the Rf and the minimum height of the reference sample (naringenin) prior to derivatisation at 254 nm and only those plates that passed the set threshold of ±0.05 for the Rf and the minimum height for MPA (Rf 0.690, minimum height 0.108) and MPB (Rf 0.550, minimum height 0.120) were used in the qualitative and quantitative analysis.

### 2.8. Quantification of Phenolic Compounds in Honey

The same chromatographic instrumentation and parameters as described in Section 2.7 were employed in the quantification of the identified phenolic compounds in the various honey samples. Standard concentrations, application volumes, derivatisation and scanning conditions were optimised. The optimised application volumes for the various standards ranged from 5.0 to 9.8 µL (1.2 µL interval) and each compound was quantified at its specific λmax using the evaluation feature of the VisionCATS software. Table 1 summarises the key parameters for the standards used in the quantification experiments.

### 2.9. HPTLC-DPPH Antioxidant Activity

The same chromatographic instrumentation and parameters as described in Section 2.7 were also employed to perform the HPTLC-DPPH analysis for antioxidant activity in the honey extracts and their respective matched constituents. Seven microliters of each honey extract were used for the analysis alongside the standards in varying volumes. After development, the plates were derivatised with 3 mL of 0.4% DPPH solution (1:1 ratio of methanol and water) using the yellow nozzle and sprayed at level 1 [32,33]. Plate images were obtained at transmittance in white light after 1 h, 2 h and 3 h. Peak profiles at 517 nm were also generated and from these the Rf values of the respective peaks were generated. Each band’s colour in the form of RGB values was determined and then converted into corresponding hue values [30]. Compounds that possess antioxidant activity will quench the DPPH radical either by electron transfer or hydrogen atom transfer through radical attack, which is observed as a discoloration at 517 nm due to the formation of 2,2-diphenyl-1-hydrazine or a substituted analogue hydrazine [33]. Gallic acid was used as positive control, its quenching activity resulting in a maximum hue value of 40° (yellow colour). All obtained hue values were calculated using previously reported formula [30]. The DPPH radical scavenging activity (% DPPH RSA) of a band of interest was calculated as follows:

(4) $$\%\;\mathrm{DPPH}\;\mathrm{RSA}=\left(\frac{\Delta H{}^{\circ}B}{H{}^{\circ}P\to 40{}^{\circ}}\right)*100$$

where: H°P→40°–Hue values (°) of unreacted DPPH on the plate (*n* = 10), ΔH°B-hue values (°) of the bands up to 40° (Note: Hue = 40° or yellow was the maximum hue value of the gallic acid).

The respective band’s % DPPH RSA was then categorised as indicated in Table 3.

The DPPH antioxidant activity of luteolin, epicatechin, epigallocatechin gallate, gallic acid, protocatechuic acid, m-coumaric acid and kojic acid was analysed at varying concentrations to validate the bioautographic analysis. Furthermore, the DPPH antioxidant activity of the matched compounds was determined at low and high concentrations to determine their inherent antioxidant activity.

### 2.10. Statistical Analysis

Analysis of variance (ANOVA) was performed using Graphpad Prism 9 (GraphPad Software, San Diego, CA, USA) in order to determine whether there was a significant difference in the total phenolic content, FRAP activity, and DPPH antioxidant activity of different honeys. Tukey’s post hoc comparisons were used to identify differences between the groups (*p* < 0.05).

## 3. Results

### 3.1. Total Phenolic Content

**Figure 2:**
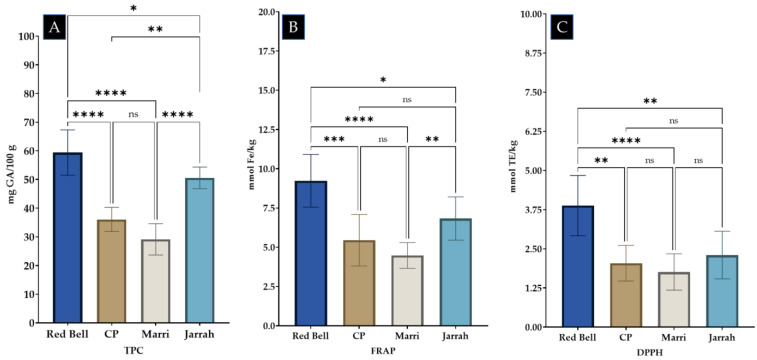
Comparison of the TPC (**A**), FRAP (**B**), and DPPH (**C**) of *Calothamnus* spp. (Red Bell), *Agonis flexuosa* (Coastal Peppermint, CP), *Corymbia calophylla* (Marri), and *Eucalyptus marginata* (Jarrah) honey. (Tukey post-hoc comparison: ns (not significant) = *p* > 0.05, * = *p* < 0.05, ** = *p* < 0.005, *** = *p* < 0.0005, **** = *p* < 0.0001).

Table 4 shows the average total phenolic content for *Calothamnus* spp. (Red Bell), *Agonis flexuosa* (Coastal Peppermint), *Corymbia calophylla* (Marri), and *Eucalyptus marginata* (Jarrah) honeys. Individual sample values, expressed as mg GAE/100 g of honey and based on the 32 samples tested, ranged from 18.91 (Marri honey) to 75.56 (Red Bell honey), with an overall average of 59.4. Individual TPC values for each investigated honey are shown in Table S2 (Supplementary Materials). The average TPC value for Red Bell honey (*n* = 8) was found to be the highest (59.4 ± 7.91 mg GAE/100 g), followed by Jarrah honey (50.58 ± 3.76 mg GAE/100 g), Coastal Peppermint honey (36.08 ± 4.2 mg GAE/100 g) and Marri honey (29.15 ± 5.46 mg GAE/100 g). The average TPC of the four honeys differed significantly when analysed using One way ANOVA (*p* < 0.0001). Tukey’s post hoc analysis demonstrated that Red Bell honey had higher TPC than the other three honeys (Coastal Peppermint and Marri honey (*p* = <0.0001), Jarrah honey (*p* = 0.0407) while Jarrah honey also showed higher TPC compared to Coastal Peppermint (*p* = 0.0016) and Marri (*p* = <0.0001) honeys). No difference, however, was observed when the mean TPC values of Coastal Peppermint and Marri honeys were compared (see Figure 2).

### 3.2. Ferric Reducing Antioxidant Power (FRAP) Assay

Table 4 shows the average FRAP antioxidant activity of the investigated Western Australian honeys, expressed as mmol Fe^2+^ equivalent/kg. Based on the analysis of the 32 individual samples tested, mean FRAP activity was 6.26 and ranged from 3.47 (*Corymbia calophylla* (Marri) honey) to 11.66 (*Calothamnus* spp. (Red Bell) honey). The FRAP antioxidant activity of individual honeys is shown in Table S2 (Supplementary Materials). When the means of each honey type were analysed, it was found that *Calothamnus* spp. (Red Bell) honey had the highest activity, followed by *Eucalyptus marginata* (Jarrah), whereas *Agonis flexuosa* (Coastal Peppermint) and *Corymbia calophylla* (Marri) honey had comparable FRAP activity. One way ANOVA analysis demonstrated a significant difference (*p* = <0.0001) between the means of the honeys and post hoc analysis showed that the average Red Bell honey’s FRAP activity was higher than that of Jarrah honey (*p* = 0.01030), Coastal Peppermint honey (*p* = 0.0001), and also Marri honey (*p* = <0.0001). The FRAP activity of Jarrah honey was also found to be higher than that of Marri honey (*p* = 0.0058), whereas Coastal Peppermint and Marri honeys had comparable average FRAP antioxidant activities (*p* = 0.5061) (see Figure 2). In line with findings reported by others [46,47,48,49], a high correlation (0.912) was observed between FRAP antioxidant activity and TPC, indicating that the antioxidant activity of these honeys is strongly related to their phenolic constituents.

### 3.3. 2,2-Diphenyl-1-Picrylhydrazyl (DPPH) Radical Scavenging Assay

Table 4 shows the average DPPH radical scavenging activity of the investigated Western Australian honeys, expressed as mmol TE/kg honey. Based on the results of the analysed 32 samples, a mean radical scavenging activity of 2.44 was found, ranging from 1.01 (*Corymbia calophylla* (Marri) honey) to 5.41 (*Calothamnus* spp. (Red Bell) honey). The DPPH radical scavenging activity of individual honey samples is shown in Table S2 (Supplementary Materials). When the mean values were compared, it was found that Red Bell honey had the highest activity. One way ANOVA analysis found a significant difference (*p* = 0.0001) amongst the means of the different honeys and post hoc analysis demonstrated that the mean DPPH antioxidant activity of Red Bell honey was higher when compared to *Eucalyptus marginata* (Jarrah) (*p* = 0.0026), *Agonis flexuosa* (Coastal Peppermint) (*p* = 0.0053), and Marri honey (*p* = <0.0001). Jarrah, Coastal Peppermint and Marri honey, have, however, comparable DPPH radical scavenging activities (*p* = >0.05) (see Figure 2). A high correlation (0.832) between DPPH antioxidant activity and TPC values of the individual honeys was observed, confirming that phenolic constituents contribute to honey’s antioxidant activity. Furthermore, a high correlation (0.948) between DPPH and FRAP antioxidant activity was also observed.

### 3.4. Phenolic Compound Identification

The phenolic compound identification was carried out based on a previously reported database filtering approach [30]. The summary of the data (as described in Section 2.7) used to determine the identity of various phenolic constituents in the four investigated pooled honey samples is shown in Tables S3–S18 (Supplementary Materials). In addition, the identified candidate compounds for each significant band in the four different Western Australian honeys are shown in Table 5 along with correlations and percent match data based on the spectral overlays of four different UV-Vis spectra of the unknown and the candidate match compounds (254 nm and 366 nm prior to derivatisation, and 366 nm after derivatisation with VSA and NP-PEG reagents).

**Figure 3:**
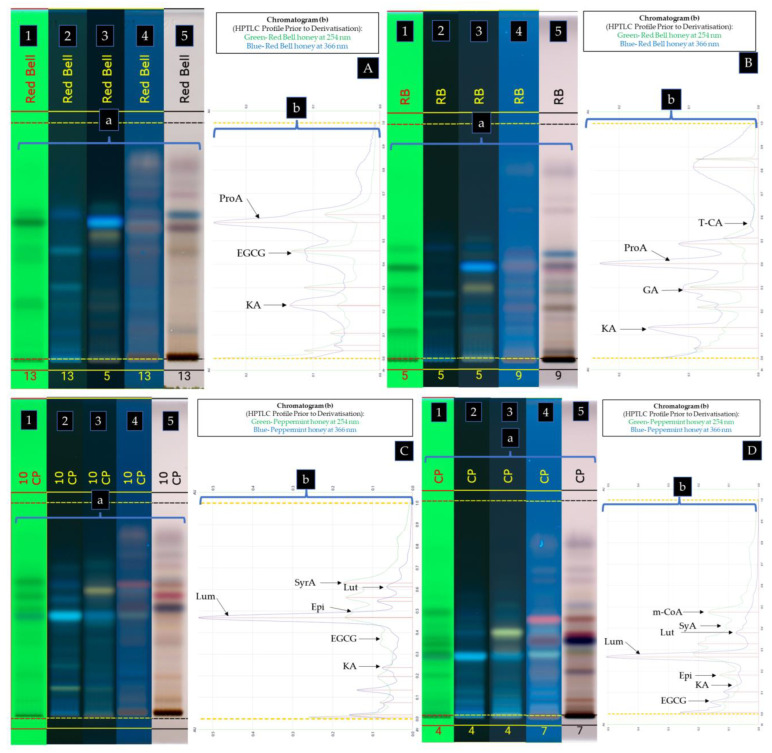
HPTLC Profile of *Calothamnus* spp. (Red Bell) honey (**A**,**B**) and *Agonis flexuosa* (Coastal Peppermint) honey (**C**,**D**) using MPA (**A**,**C**), and MPB (**B**,**D**). (**a**) Plate images obtained under the following light conditions: 254 nm prior to derivatisation (**1**), 366 nm prior to derivatisation (**2**), 366 nm after derivatisation with NP-PEG (**3**), 366 nm after derivatisation with VSA (**4**), transmittance in white light after derivatisation with VSA (**5**); (**b**) Chromatograms prior to derivatisation obtained at 254 nm and 366 nm.

**Figure 4:**
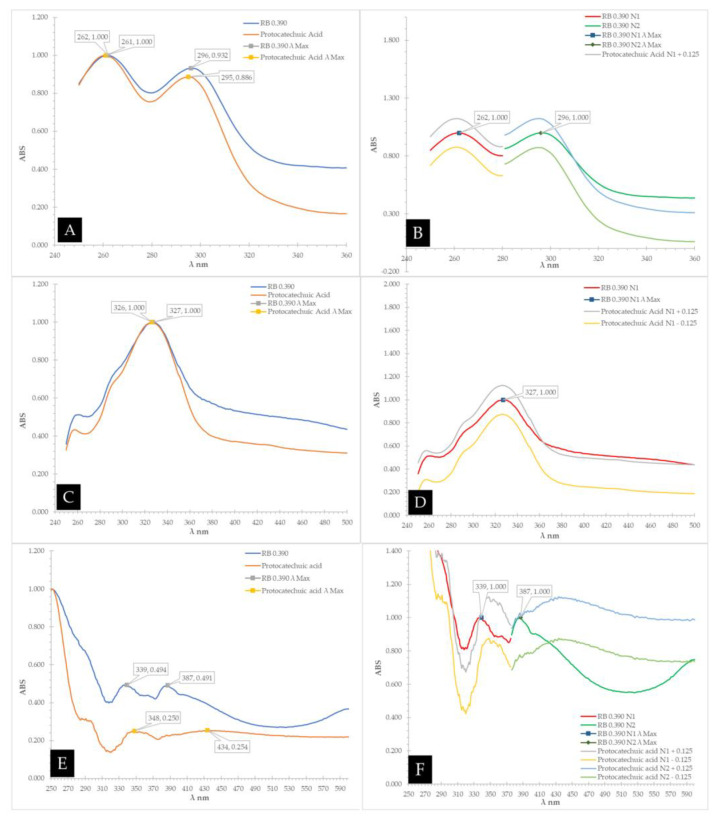
Spectra overlay of unknown band at Rf 0.390 in *Calothamnus* spp. (Red Bell) honey vs. protocatechuic acid (**10**) using MPB. (**A**)—UV-Vis spectra and (**B**)—overlay of the ±0.125 AU comparison prior to derivatisation, (**C**)—UV-Vis spectra, (**D**)—overlay of the ±0.125 AU comparison after derivatisation with NP-PEG, (**E**)—UV-Vis spectra, (**F**)—overlay of the ±0.125 AU comparison after derivatisation with VSA.

**Figure 5:**
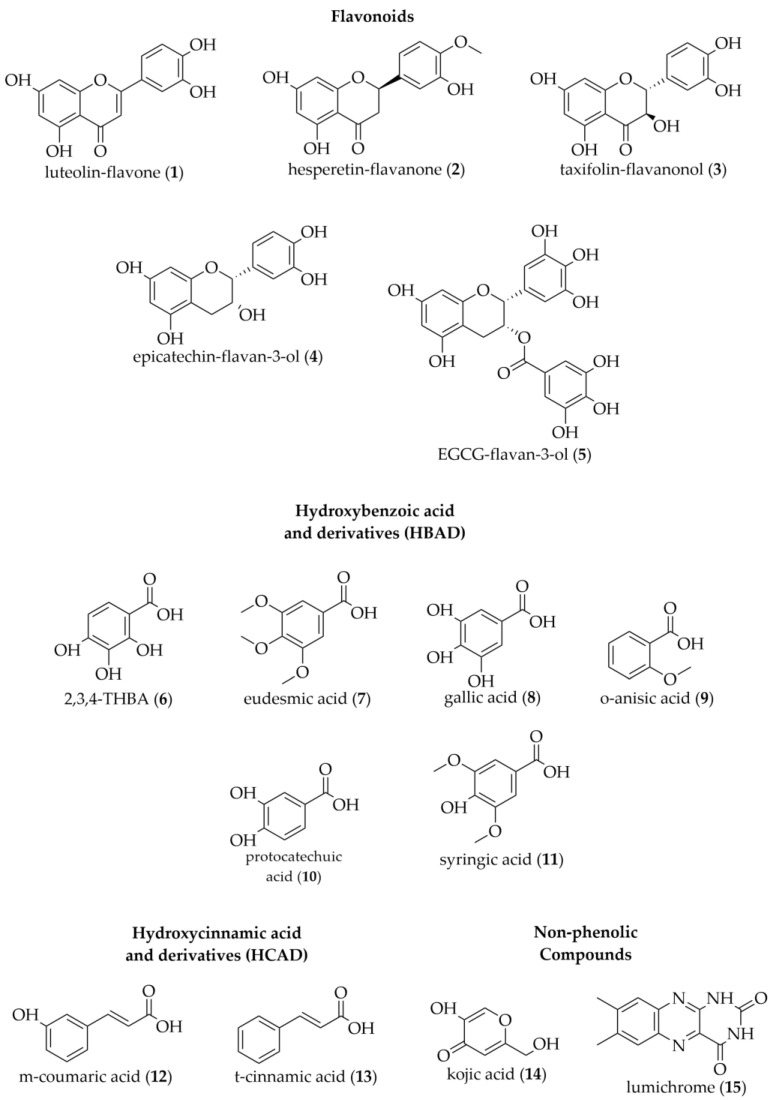
Structures of the compounds identified in Western Australian honeys (generated using ChemDraw version 20.1.1, PerkinElmer Informatics, Inc., Waltham, MA, USA).

Based on the results obtained using database 1A and 1B (Figure 3A), the compound at Rf 0.570 in *Calothamnus* spp. (Red Bell) honey was identified as protocatechuic acid (**10**) as shown by the similarity of the spectral overlays of the unknown band and the standard when analysing their UV-Vis spectra prior to derivatisation (Figure 4A,B), after derivatisation with NP-PEG (Figure 4C,D), and also after derivatisation with VSA (Figure 4E,F). The unknown band at Rf 0.423 in Red Bell honey was identified as epigallocatechin gallate (**5**), and the unknown band at Rf 0.226 as kojic acid (**14**). By employing database 2A and 2B (Figure 3B), which utilised a less polar solvent, the unknown band at Rf 0.550 in Red Bell honey was identified as t-cinnamic acid (**13**), the band at Rf 0.380 as protocatechuic acid (**10**), the band at Rf 0.270 as gallic acid (**8**), and the band at Rf 0.115 as kojic acid (**14**) (see Figure 5 for structures).

**Figure 6:**
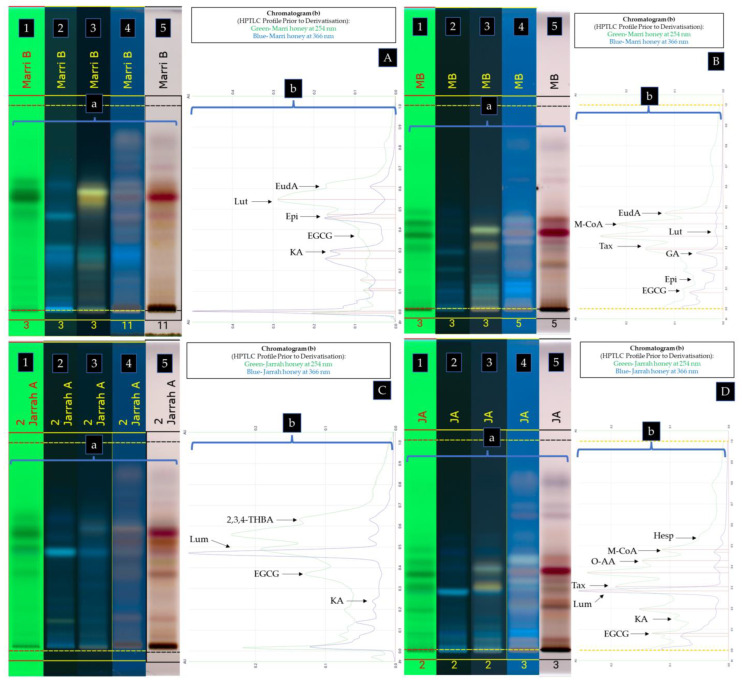
HPTLC Profile of *Corymbia calophylla* (Marri) honey (**A**,**B**) and *Eucalyptus marginata* (Jarrah) honey (**C**,**D**) using mobile phase A (**A**,**C**), and mobile phase B (**B**,**D**). Plate images (**a**) obtained under the following light conditions: 254 nm prior to derivatisation (**1**), 366 nm prior to derivatisation (**2**), 366 nm after derivatised with NP-PEG (**3**), 366 nm after derivatisation with VSA (**4**), transmittance in white light after derivatisation with VSA (**5**) and chromatograms (**b**) prior to derivatisation obtained at 254 nm and 366 nm.

All other compounds reported here were identified in the three other honey samples in the same manner. Table 2 summarises the identified honey constituents. Figure 3C,D shows the identified compounds in *Agonis flexuosa* (Coastal Peppermint) honey. Figure 6A,B summarises the identified compounds in *Corymbia calophylla* (Marri) honey, while Figure 6C,D summarises the identified compounds in *Eucalyptus marginata* (Jarrah) honey.

**Figure 7:**
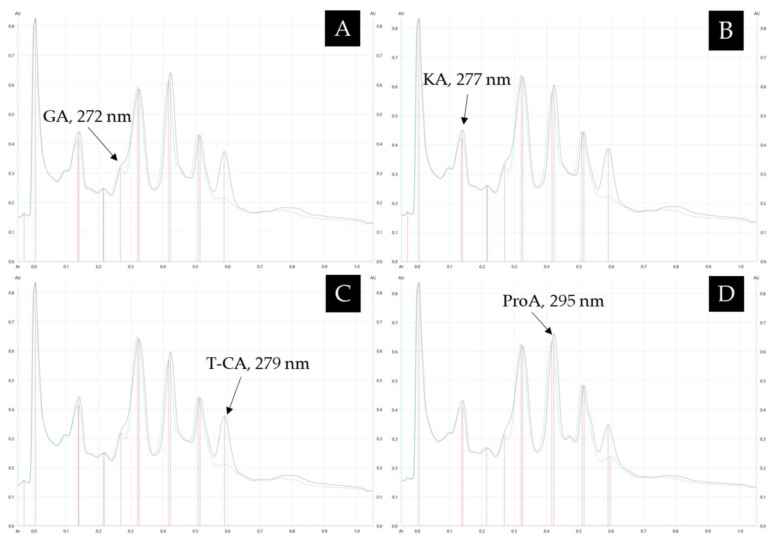
(**A**–**D**) Peak profile comparison of *Calothamnus* spp. (Red Bell) honey (green) and *Calothamnus* spp. (Red Bell) honey spiked with the identified compounds based on Database 2A and 2B (blue) scanned at the λmax of each specific compound prior to derivatisation.

A comparison between the peak profile of each honey and the respective honey over-spotted with a mixture of its identified constituents was also used to further confirm the phenolic compound determination. For confirmation, scans were performed, for example, at each specific λmax of each identified compound in *Calothamnus* spp. (Red Bell) honey (Figure 7A–D) using databases 2A and 2B and based on this analysis, the Rf of the matched compounds were found to be similar to that of the identified bands in the honey. Moreover, an increase in the absorbance confirmed the presence of the compounds in the honey. Profile comparisons for the other investigated honeys are included in the Supplementary Materials (Figures S5–S11).

### 3.5. Phenolic Compound Quantification

**Figure 8:**
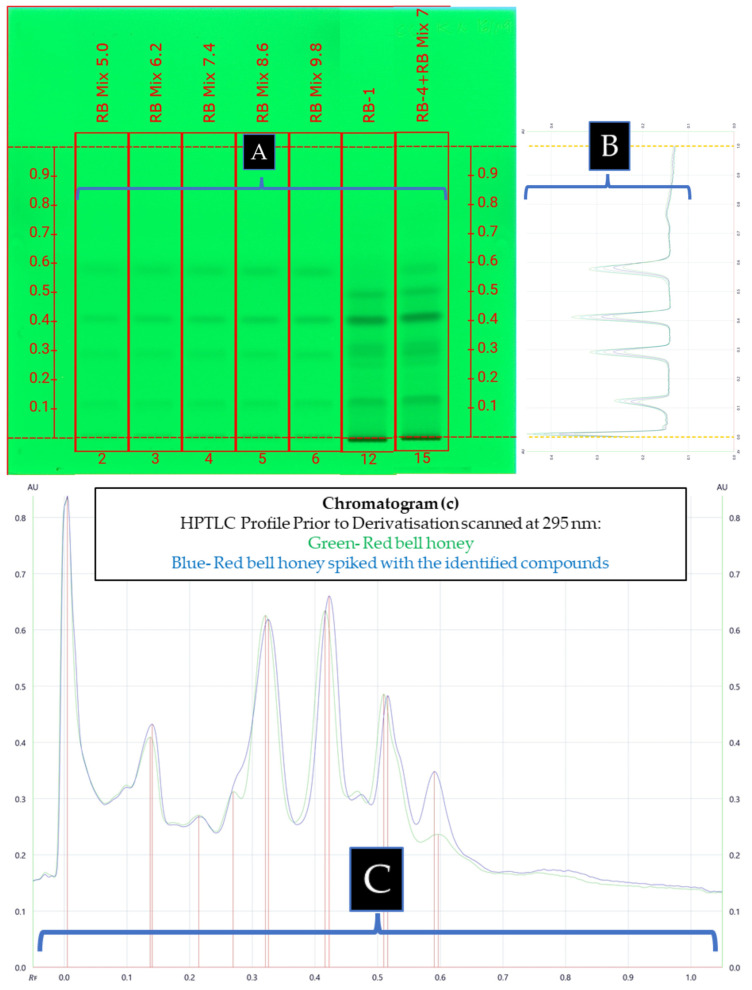
(**A**) HPTLC Images of the compound mixture of identified compounds in *Calothamnus* spp. (Red Bell) honey using various application volumes (Tracks 2–6) as compared to Red Bell honey (Track 12), and Red Bell honey spiked with the mixture of identified compounds (Track 15); (**B**) peak profile of compounds identified in *Calothamnus* spp. (Red Bell) honey; (**C**) peak profile of Red Bell honey (green), and Red Bell honey spiked with the identified compound mixture (blue) scanned at 295 nm using MPB.

Optimised parameters, such as standard concentrations, application volumes, mode in obtaining the profile/chromatogram, and derivatisation for quantification of the identified phenolic compounds in the four pooled honey samples, are shown in Table S19 (Supplementary Materials). Based on the findings of the optimisation, it was concluded that standard concentrations ranging from 5 µg/mL to 50 µg/mL, application volumes ranging from 5.0 to 9.8 µL (1.2 µL interval), peak profiles obtained by scanning the plate at the respective specific λmax, and the absence of any derivatisation constituted the best approach for quick and accurate quantification of phenolic compounds in the honey matrices. Linearity was observed to be greater than 0.99 for each standard and the percent recovery ranged from 95.2 to 102.6%. Table 2 details the standard concentrations, linearity, and % recovery of each identified constituent that was used in the quantification experiment. Furthermore, a sample of an HPTLC plate and its corresponding peak profile used in the quantification of phenolic compounds in *Calothamnus* spp. (Red Bell) honey is shown in Figure 8A–C.

By utilising the optimised conditions, the concentration of the compounds identified in honey ranged from 0.003 µg/g (t-cinnamic acid (**13**)) to 13.49 µg/g (2,3,4-trihydroxy benzoic acid (**6**)) (see Table 6 for the specific quantities).

Protocatechuic acid (**10**) was found to be the most abundant constituent in *Calothamnus* spp. (Red Bell) honey, followed by epigallocatechin gallate (**5**), kojic acid (**14**), gallic acid (**8**), and t-cinnamic acid (**13**). In the case of *Agonis flexuosa* (Coastal Peppermint) honey, epicatechin (**4**) was found to be the most abundant, followed by kojic acid, epigallocatechin gallate, lumichrome (**15**), syringic acid (**11**), luteolin (**1**), and m-coumaric acid (**12**). For *Corymbia calophylla* (Marri) honey, gallic acid was found to be the most abundant compound, followed by epigallocatechin gallate, eudesmic acid (**7**), epicatechin, luteolin, taxifolin (**3**), kojic acid, and m-coumaric acid (**12**). 2,3,4-Trihydroxy benzoic acid (**6**) was found to be the most abundant compound in *Eucalyptus marginata* (Jarrah) honey, followed by epicatechin, o-anisic acid (**9**), taxofolin, lumichrome, m-coumaric acid, kojic acid, and hesperitin (**2**) (see Figure 5 for structures).

### 3.6. HPTLC DPPH Assay

The DPPH-HPTLC assay was carried out to determine which constituents contributed to the respective honey’s overall antioxidant activity. Previously, the HPTLC-DPPH assay was developed to determine the DPPH antioxidant activity after 1 h of exposure to the reagent, the corresponding peak profiles were obtained using white light, and the antioxidant activity was expressed as mg GAE/100 g of honey [32]. In this experiment, incubation time as well as the mode for peak profile generation were optimised (Figure S12, Supplementary Materials). It was found that the colour of the unreacted DPPH on the plate degraded by 14.7% after 2 h and by 19.3% after 3 h. It was also observed that a significant decrease in the absorbances of the test compounds was observed after 2 and 3 h of incubation time as compared to 1 h (Figure S8, Supplementary Materials). Furthermore, naringenin (used as reference standard) showed a DPPH radical scavenging activity (% DPPH RSA) of 66.5% after 1 h, 81.4% after 2 h, and 81.9% after 3 h of incubation. DPPH scavenging activity is generally evaluated at the point when the absorbance remains constant [50]. Because of this, in this study the photo-documentations and the recording of the corresponding peak profiles were carried out after 2 h in order to allow sufficient time for compounds to react with the DPPH reagent but not too long that the reagent would autodegrade and produce false positive results. Furthermore, it was also observed that peak profiles generated using a scan at 517 nm were more sensitive in determining the reaction of the individual bands as compared to profiles generated using white light, and therefore scanning at 517 nm was adopted for all analyses in this study (Figure S12, Supplementary Materials).

The use of a change in hue to determine the DPPH antioxidant activity was also validated. Based on the findings (Table 7), all standards showed an increase in % DPPH RSA which correlated with increases in sample concentration, indicating that hue values can be a very useful tool in describing the antioxidant activity of a particular compound.

The DPPH antioxidant activity of the four pooled and extracted Western Australian honeys along with the phenolic compounds that were previously identified in each honey were analysed in the HPTLC-DPPH assay using two solvent systems (MPA and MPB). Figure 8 shows the DPPH-HPTLC plate of Red Bell honey. The images of the other honeys are shown in Figures S13–S15.

*Calothamnus* spp. (Red Bell) honey was found to have nine bands with DPPH antioxidant activity of which the band at Rf 0.390 had very high activity, a medium activity band was found at Rf 0.115, and low activity bands at Rf 0.505, 0.450, 0.281, 0.246, 0.207, and 0.174. A total of nine bands were also observed to be antioxidant in the case of *Agonis flexuosa* (Coastal Peppermint) honey, where the bands at Rf 0.473, 0.395, 0.352, 0.279, 0.187, 0.114, 0.100, and 0.090 were all found to be low in activity. *Corymbia calophylla* (Marri) honey presented 11 antioxidant bands, of which a medium active antioxidant band was found at Rf 0.391, while bands at Rf 0.484, 0.444, 0.391, 0.313, 0.275, 0.26, 0.212, 0.179, 0.146, 0.105 were all low in activity. *Eucalyptus marginata* (Jarrah) honey, on the other hand, showed 12 antioxidant bands, of which the band at Rf 0.391 had medium activity and low activity bands were observed at Rf 0.530, 0.455, 0.322, 0.282, 0.254, 0.22, 0.189, 0.147, 0.117, 0.100, 0.083, and 0.024. The average antioxidant band activity of each honey (% AVE) was also calculated based on the total % DPPH RSA over the total number of antioxidant bands. It was found that *Calothamnus* spp. (Red Bell) honey had an average of 33.6% DPPH RSA, *Eucalyptus marginata* (Jarrah) honey had an average of 21.1%, *Agonis flexuosa* (Coastal Peppermint) honey an average of 18.4%, and *Corymbia calophylla* (Marri) honey an average of 18.2%, a trend which is similar to that reported for the total DPPH antioxidant activity of each individual honey (Table 8).

The DPPH antioxidant activity of the compounds identified in each pooled honey sample was also determined at a low concentration to mimic the concentration of the compounds in each honey and also at a high concentration in order to determine whether the activity is based on its concentration in the honey or an inherent antioxidant activity of the constituent (see Table 9). Based on the data generated, it was found that most compounds were antioxidant with the exception of eudesmic acid, o-anisic acid, t-cinnamic acid, and lumichrome, which remained inactive even when analysed at a higher concentration (see Figure 5 for structures).

**Figure 9:**
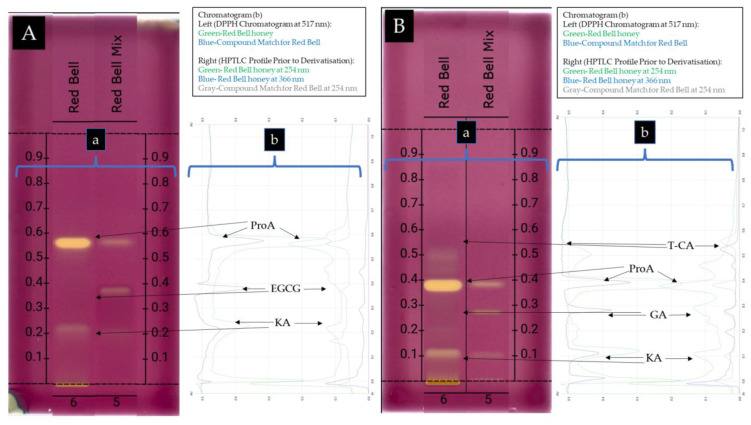
HPTLC-DPPH plate image (a) of *Calothamnus* spp. (Red Bell) honey after development in MPA (**A**) and after development in MPB (**B**) recorded with transmission white light, and comparison of the peak profiles of *Calothamnus* spp. (Red Bell) honey (green) and Red Bell honey spiked with the identified compounds (blue) after derivatisation with DPPH reagent and scanning at 517 nm (b-left) and comparison of the profiles of *Calothamnus* spp. (Red Bell) honey obtained at 254 nm (green) and 366 nm (blue) prior to derivatisation and the profile of *Calothamnus* spp. (Red Bell) honey spiked with the identified compounds (gray) obtained at 254 nm prior to derivatisation (b-right).

For *Calothamnus* spp. (Red Bell) honey, one of the identified constituents, t-cinnamic acid at Rf 0.562, was observed to be inactive, which was consistent with the finding that the t-cinnamic acid (**13**) standard did not possess any DPPH antioxidant activity. The other compounds in Red Bell honey, identified as protocatechuic acid (**10**) at Rf 0.390, gallic acid (**8**) at Rf 0.281, and kojic acid (**14**) at Rf 0.115, were found to be the dominant antioxidants in the honey (Figure 9B).

For *Agonis flexuosa* (Coastal Peppermint) honey, the respective quantities present for the bands at Rf 0.473 (m-coumaric acid (**12**)), at Rf 0.420 (syringic acid (**11**)), at Rf 0.395 (luteolin (**1**)), at Rf 0.187 (epicatechin (**4**)), at Rf 0.100 (kojic acid (**14**)), and at Rf 0.090 (epigallocatechin gallate (**5**)), showed the expected antioxidant activity based on the calibrated antioxidant activity of the standards, except for the constituent at Rf 0.279 (identified as lumichrome (**15**)) which showed an unexpected result since lumichrome standard itself was found to be inactive, indicating that there might be a constituent that was co-eluting with lumichrome at this Rf value which might cause the honey band at this Rf to show antioxidant activity (Figure S13, Supplementary Materials).

In the case of *Corymbia calophylla* (Marri) honey, the bands at Rf 0.484 (eudesmic acid (**7**)), Rf 0.444 (m-coumaric acid), Rf 0.391 (luteolin), Rf 0.313 (taxifolin (**3**)), Rf 0.275 (gallic acid (**8**)), Rf 0.179 (epicatechin), and Rf 0.105 (kojic acid) all showed the expected antioxidant activity consistent with that of the calibrated antioxidant activity of the standards (Figure S14, Supplementary Materials).

For *Eucalyptus marginata* (Jarrah) honey, the band at Rf 0.530, identified as hesperitin (**2**), was found to be consistent in its behaviour with the analysed activity of the corresponding standard, which was found to be inactive at low concentration. The band at Rf 0.455 (o-anisisc acid (**9**)) was found to be active, whereas the corresponding standard showed no activity, even at higher concentration, implying that this honey band has a co-eluting constituent which causes a low level of antioxidant activity. The band at Rf 0.282 was identified as lumichrome (**15**), which showed a very low level of activity similar to the standard, which was found to be inactive in the investigated concentrations. The activity of the compounds at Rf 0.420 (m-coumaric acid (**12**)), Rf 0.322 (taxifolin (**3**)), Rf 0.100 (kojic acid (**14**)), and Rf 0.083 (epigallocatechin gallate (**5**)) were found to be consistent with the activity of the respective standards (Figure S15, Supplementary Materials).

## 4. Discussion

The data obtained in the TPC assay were consistent with previous studies where Red Bell honey had shown higher phenolic content than nine other monofloral honeys from Western Australia [37]. By using the same conditions for the assay, Manuka honey from Australia and New Zealand [43] was found to have a TPC of 35.08 mg GAE/100 g (minimum 22.6, maximum 66.3) indicating that *Calothamnus* spp. (Red Bell) honey and also *Eucalyptus marginata* (Jarrah) honey have higher TPC than Manuka honey, which is generally seen as a honey with high antioxidant activity [51]. By comparing the findings of this study with TPC data for other monofloral honeys across the globe, TPC values of 18.9 ± 3.82 to 23.7 ± 4.37 GAE/100 g [52] were reported for some Romanian monofloral honeys, while Mexican monofloral honeys had TPC range of 18.02 ± 0.49 to 102.77 ± 1.29 GAE/100 g [53], Czech and Slovak honeys had TPC between 54.0 ± 1.7 and 254.2 ± 1.4 GAE/100 g [54], and Brazilian honeys were reported to have TPC between 13.3 and 100 GAE/100 g [55]. The TPC values obtained in this study are lower in comparison. The assay used in this study was, however, a modified Folin–Ciocalteu assay in which the concentration of the sodium carbonate solution was optimised in such a way that sugar interference was muted, as sugars were also observed to react with the reagent leading to an overestimation of TPC without this modification [41].

Similar to the generated TPC values, the FRAP activity of *Calothamnus* spp. (Red Bell) honey was also observed to be higher compared to the other investigated Western Australian honeys [37] and also when compared to that of Manuka honey (2.88 to 10.72 mmol Fe^2+^/kg) [43]. By comparing the FRAP activity with that of other monofloral honeys from across the globe, Bangladeshi monofloral honeys were reported to have FRAP activity of 1.00–8.00 mmol Fe^2+^/kg [56], the FRAP activity of Oak honeydew honey from Croatia was reported to be 4.8 mmol Fe^2+^/kg [57], Polish monofloral honeys were reported to have between 1.00 and 7.00 mmol Fe^2+^/kg FRAP activity [58], and Thai monofloral honeys 0.61 to 4.34 mmol Fe^2+^/kg [59]. Compared to these findings, the honeys from Western Australia investigated in this study showed a higher FRAP activity.

The DPPH radical scavenging activity of *Calothamnus* spp. (Red Bell) honey was also observed to be higher compared to the other investigated Western Australian honeys [37] and when compared to that of Manuka honey (mean = 1.98, range of 0.56 to 4.35 mmol TE/kg) [43]. Polish monofloral honeys were reported to have 0.20 to 1.20 mmol TE/kg DPPH activity [58], Oak honeydew honey from Croatia was reported to have a DPPH activity of 4.5 mmol TE/kg [57], and Thai monofloral honeys of 0.107 to 1.224 mmol TE/kg [59]. These values were lower compared to the DPPH radical scavenging activity of the investigated WA honeys.

High correlations between TPC values, FRAP and DPPH antioxidant activity were observed in this study, consistent with other reports [46,47,48,49]. DPPH and FRAP assays were chosen to express the total antioxidant activities of honey because the application of multiple assays can be helpful in reflecting the antioxidant properties of honeys more accurately than a single assay can do [60]. DPPH and FRAP assays have been widely used to determine the antioxidant activity of various plant extracts and food products since they use stable free radicals and the determination of antioxidant capacity is simple, quick and easy to perform, results are readily validated, accurate, and highly reproducible and the reagents are inexpensive and easy to prepare [61,62]. The TPC assay was employed to confirm that the antioxidant assay can be attributed to the phenolic compounds present in honey as it has been found that high antioxidant potential in FRAP and DPPH assays is usually observed for samples with high phenolic and flavonoid content [11].

By employing the HPTLC database to identify the phenolic constituents in the honeys, kojic acid (**14**) and epigallocatechin gallate (**5**) were found in all investigated honeys. m-Coumaric acid (**12**) was present in most honeys except *Calothamnus* spp. (Red Bell) honey. Lumichrome (**15**) was identified in *Agonis flexuosa* (Coastal Peppermint) and *Eucalyptus marginata* (Jarrah) honey, gallic acid (**8**) was found in both Red Bell and *Corymbia calophylla* (Marri) honey, taxifolin (**3**) was only found in Marri and Jarrah honey, while luteolin (**1**) and epicatechin (**4**) were only found in Coastal Peppermint and Marri honey. Hesperitin (**2**) was only identified in Jarrah honey, eudesmic acid (**7**) only in Marri honey, syringic acid (**11**) only in Coastal Peppermint honey, and protocatechuic acid (**10**) and t-cinnamic acid (**13**) only in Red Bell honey. Compounds that were only identified in a specific honey might in the future potentially be used as biomarkers for that honey.

The HPTLC-based database for phenolic compound identification was previously employed in the analysis of Manuka honey where kojic acid, gallic acid, epigallocatechin gallate, lumichrome, 2,3,4-trihydroxy benzoic acid, and o-anisic acid were also identified. However, leptosperine, mandelic acid, lepteridine, methyl syringate, salicylic acid, and benzoic acid were only found in Manuka honey [30]. This implies that there are some compounds that are ubiquitous in honeys while others are unique and can only be found in a certain honey. Since all the honeys investigated in this study along with Manuka honey belong to the plant family Myrtaceae, it can be speculated that this might explain some of the overlaps in the compounds identified in the four honey types.

To date, reports on the presence and concentration of phenolic compounds in honeys originating from Western Australia has been very scant. Prior to this study, only for Jarrah honey had some compounds been reported. Using HPLC-ESI-MS/MS analysis, Anand et al. in 2019 were able to quantify quercetin, hesperitin, cinnamic acid, methyl syringate, rutin, sinapic acid, ferulic acid, p-coumaric acid, phenyllactic acid, syringic acid, caffeic acid, vanillic acid, chlorogenic acid, p-hydroxybenzoic acid, protocatechuic acid, and gallic acid [39]. By employing the HPTLC-based database in this study, only hesperitin was identified from the compounds reported by Anand et al., which can be attributed to a number of reasons: Firstly, Anand et al. (2019) utilised a Strata-X cartridge solid phase extraction which was eluted with acidified water (pH-2), and then with methanol prior to their analysis [39]. This study, however, employed a liquid–liquid solvent extraction using dichloromethane and methanol (1:1) as a solvent system.

The solvent system used in the development of the HPTLC plates was toluene: ethyl acetate: formic acid 6:5:1 (MPB) which has frequently been used to fingerprint honeys [32,33,40,63,64]. A more polar solvent system, toluene: ethyl acetate: formic acid 2:8:1 (MPA), was also utilised in order to identify compounds of higher polarity. A better separation in the bands in honey was observed with MPB, however, it was found to be unable to fully develop all honey constituents as seen by dark bands on the baseline of the plate prior to derivatisation, and after derivatisation with NP-PEG and VSA reagents (Figure 2 and Figure 5), as well as after derivatisation with DPPH reagent (Figure 9). It is a recommendation that another solvent system with higher polarity is also used in the future in order to identify those more polar compounds that were not fully captured by the solvent systems used in the current study.

This research utilised pooled honey samples, as the composition of such a pooled sample will be more representative of the typical chemical composition of the respective honey compared to the analysis of a randomly chosen single sample. Specifically, eight samples were pooled to represent the Red Bell honey used in this study, six samples each were pooled for Coastal Peppermint and Jarrah honey, and 13 samples were used to represent Marri honey.

An HPTLC-DPPH assay was previously employed in the qualitative and quantitative analysis of the antioxidant fingerprints of honeys [32,33,34]. Islam et al. in 2020 and 2021 utilised the method for the quantification of antioxidant band activities for various Australian honeys. However, the analysis was performed using dichloromethane as an extraction solvent, the incubation time was set to only 1 h, and peak profiles were obtained with white light [32,33]. In this study, however, a more polar extraction solvent was used (dichloromethane: methanol 1:1 *v*/*v*) which led to the observation of more antioxidant bands. The incubation time was also optimised as it was found that 1 h was not enough for some phenolic compounds to fully react with the DPPH reagent (Figure S8, Supplementary Materials). Longer incubation times of 2 h and 3 h were also tested and it was found that 2 h is the optimum time for the multiple types of polyphenols present in honey to react with the reagent but not long enough for the DPPH reagent to autodegrade.

The findings of the HPTLC-DPPH assay for honeys are often expressed in a qualitative manner by presenting active bands that showed a discoloration of the DPPH reagent [34]. In some instances, the antioxidant band activity was also quantified, expressed as mg GAE/100 g of honey [32,33]. Quantification of individual bands is, however, challenging given that some antioxidant bands are very low in absorbance and might thus be below the limit of detection of this quantification method.

In this study, the colours of the unreacted DPPH reagent and the colours of the active bands in the analysed samples, converted into hue values, were compared and from this, their DPPH radical scavenging activity was calculated. The HPTLC software usually provides colour information in the form of RGB values which can be converted into hue values (based on the hue, saturation, and brightness (HSB) colour space) [30]. The use of hues in expressing the colour of a particular band was found to be very helpful in the early stage of identification of an unknown sample using the HPTLC-derived database where, upon the use of a suitable derivatisation agent, a discrimination of one compound group from another based on colour was possible [30]. It was found in this study that colour captured in the form of hue values can also be used to express the results of the HPTLC-DPPH assay (Table 6, Table 7 and Table 8). Various antioxidant compounds were tested, and the findings demonstrated that hues varied according to the sample concentration that was applied. However, linear regression did not reach 0.99 indicating that the current parameters used in this study are only able to describe the antioxidant results in a semi-quantitative manner, expressed here in inferences ranging from + to +++. More optimisation is required in order to use the method for full quantification of the antioxidant activity of individual bands in an unknown sample.

The DPPH antioxidant activities of the individual constituents that were identified in each honey were also determined as a mixture at low concentration to mimic the concentrations that were quantified in honey and also at higher concentration (Table 7) to determine whether its activity is based on its concentration in the honey or inherent antioxidant activity of the constituent. All identified compounds except eudesmic acid (**7**), o-anisic acid (**9**), t-cinnamic acid (**13**), and lumichrome (**15**) showed activity towards the DPPH reagent. The inactive compounds (Table 7) lack a hydroxyl group in the phenolic ring that can react with the DPPH reagent [65,66] indicating that not all phenolic compounds are antioxidant.

**Figure 10:**
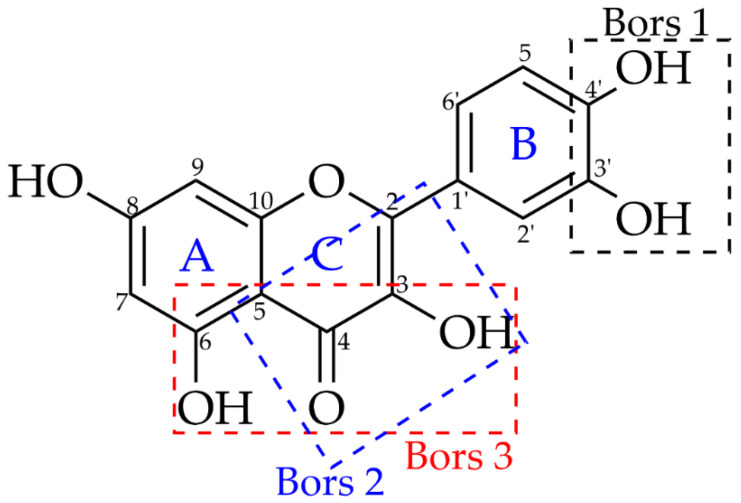
Bors Criteria to describe flavonoid activity (adapted from Platzer et al. [65]).

The reaction of compounds with DPPH is governed by the reagent’s steric accessibility indicating that smaller molecules have greater access to the radical site as compared to larger molecules [50]. This explains why flavonoids tend to react slower compared to smaller molecules like simple phenolic acids. The reactivity of flavonoids with DPPH on the other hand is dictated by the so-called Bors criteria (Figure 10).

The first criterion is the presence of a catechol group on Ring B (Bors 1), which increases the stability of the resulting antioxidant radical. The second is the presence of a 2,3 double bond combined with a 4-oxo group on Ring C (Bors 2), which facilitates electron delocalization. The third is the presence of OH groups at positions 3 and 5 in combination with a 4-oxo group, which enables electron delocalization via hydrogen bonds (Bors 3) [65,66]. Among the flavonoids identified in this study, taxifolin (**3**) possesses Bors 1 and 2 criteria, confirmed by very high radical scavenging activity even when analysed at a lower concentration. Luteolin (**1**), epicatechin (**4**), and epigallocatechin gallate (**5**) all possess the Bors 1 criterion, while hesperetin (**2**) does not possess any, which explains why it has shown only a very weak radical scavenging activity. The trends seen in the antioxidant activity of the different phenolic compounds investigated in this study were consistent with the trends that were previously reported [65,66,67]. The HPTLC-DPPH assay has thus been demonstrated to be a very powerful tool in the identification of antioxidant constituents. However, DPPH or a similarly structured radical does not exist in a biological or food system [50] and it is therefore suggested that a more biochemically relevant antioxidant model should be used in future studies.

This is the first report on the use of band colours as a basis of expressing antioxidant activity in samples, which demonstrates that colour values derived from HPTLC analysis can also be used to (semi-quantitatively) express antioxidant activity in addition to more traditional quantification (using generated standard curves) that HPTLC can also perform. While this presents a novel analytical angle to HPTLC-DPPH analysis, some limitations need to be acknowledged. Given the very general nature of the DPPH assay and its common use in natural product research as a screening tool for antioxidant activity, a qualitative (i.e., active or inactive) or semi-quantitative (i.e., activity ranges from + to +++) approach might suffice in many instances. This can be achieved, as illustrated in this study, by expressing antioxidant activity of individual honey bands as % RSA, which is a widely accepted way of expressing antioxidant activity. However, should a fully quantitative result be the aim, more optimisation is needed, specifically to determine the concentration range of each match compound that yields linear regression equal to or greater than 0.99. 

In recent years High Performance Thin Layer Chromatography (HPTLC) has emerged as a very versatile tool for various aspects of honey analysis. It can, for example, be used to identify and quantify various sugars in honey [68,69] and with this can also be used to identify post-harvest sugar adulterations [70]. It is also applied to identify and quantify the presence of hydroxymethyl furfural (HMF) in honey, which is a marker for excessive heat treatment-associated degradation and thus reduced honey quality [71,72,73]. HPTLC in combination with DPPH derivatisation has also been successfully used to visualise and quantify (as gallic acid equivalents) antioxidant honey constituents [32,33]. Moreover, the HPTLC analysis of organic honey extracts has been demonstrated to yield unique signatures that are reflective of a honey’s floral origin and can thus be used for honey authentication [40,63,64]. This study contributes to the growing body of literature that demonstrates the versatility of HPTLC in the analysis of honey. The identity of some phenolic constituents in the four investigated Western Australian honeys was revealed using a HPTLC-based database along with their quantification, also using HPTLC. Moreover, compounds that contribute to these honeys’ antioxidant activity could be identified and semi-quantified using a modification of the previously published HPTLC-DPPH analysis protocol.

## 5. Conclusions

This study investigated the antioxidant activity of four Western Australian honeys, *Calothamnus* spp. (Red Bell), *Agonis flexuosa* (Coastal Peppermint), *Corymbia calophylla* (Marri) and *Eucalyptus marginata* (Jarrah) honey. It was found that Red Bell honey has the highest total phenolic content, followed by Jarrah, Coastal Peppermint, and Marri honey. The same trends were observed for their respective FRAP and DPPH antioxidant activities.

t-Cinnamic acid, protocatechuic acid, gallic acid, epigallocatechin gallate, and kojic acid were identified and quantified in Red Bell honey. For Coastal Peppermint honey, the presence of syringic acid, m-coumaric acid, luteolin, epicatechin, lumichrome, and kojic acid was determined and quantified. Eudesmic acid, epicatechin, epigallocatechin gallate, luteolin, gallic acid, kojic acid, m-coumaric acid, and taxifolin were identified and quantified in Marri honey, and hesperitin, o-anisic acid, taxifolin, kojic acid, m-coumaric acid, lumichrome, epigallocatechin gallate, kojic acid, and 2,3,4-trihydroxy benzoic acid in Jarrah honey.

HPTLC-DPPH bioautography was also carried out to determine which honey constituents contribute to the respective honey’s antioxidant activity using a novel method of analysis based on the changes of hues on reaction with the DPPH reagent. This change in hue was used to determine the % RSA of each active band. The method was able to identify the individual bands that contribute to the honeys’ overall antioxidant activity. Based on the findings of this analysis, most identified compounds showed antioxidant activity except for t-cinnamic acid, lumichrome, o-anisic acid, and eudesmic acid due to the absence of hydroxyl groups in their benzene ring.

As most analyses were carried out using HPTLC, the study was also able to demonstrate the versatility of this instrumentation in the analysis of various aspects of honey chemistry and bioactivity.

## Figures and Tables

**Table 1:** Key parameters for the phenolic standards applied in the quantification experiments.

Compound and Code	Purity (%)	Supplier	Mobile Phase	Concentration (µg/mL)	Rf	UV Detection (nm)	R^2^	% Accuracy (*n* = 3)
Luteolin– Lut (**1**)	97	C	MPB	12.5	0.375	352	0.998	97.4
Hesperetin– Hesp (**2**)	95	C	MPB	12.5	0.527	290	0.999	98.9
Taxifolin– Tax (**3**)	95	E	MPB	5	0.335	292	0.998	98.5
Epicatechin– Epi (**4**)	98	D	MPB	50	0.176	281	0.990	97.8
Epigallocatechin gallate– EGCG (**5**)	98	B	MPB	50	0.065	282	0.996	99.3
2,3,4-Trihydroxy benzoic acid– 2,3,4-THBA (**6**)	98	B	MPA	25	0.589	267	1.000	98.4
Eudesmic acid– EudA (**7**)	98	C	MPB	50	0.478	264	0.991	95.8
Gallic acid– GA (**8**)	NI	F	MPB	25	0.27	272	0.997	98.9
o-Anisic acid– o-AA (**9**)	NI	A	MPB	25	0.44	299	1.000	98.9
Protocatechuic acid– ProA (**10**)	98	C	MPB	25	0.377	295	0.997	97.7
Syringic acid– SyrA (**11**)	98	C	MPB	25	0.395	277	0.998	96.3
m-Coumaric acid– m-CoA (**12**)	98	C	MPB	25	0.467	280	0.996	95.2
t-Cinnamic acid– TCA (**13**)	99	C	MPB	12.5	0.557	279	0.994	97.2
Kojic acid– KA (**14**)	NI	A	MPB	25	0.13	277	0.995	97.0
Lumichrome– Lum (**15**)	NI	A	MPB	10	0.266	357	0.993	97.5

Legend: NI-No information, Suppliers: A = Sigma Aldrich (Castle Hill, NSW, Australia), B = Angene International Ltd. (Nanjing, China), C = Combi-Blocks Inc., (San Diego, CA, USA), D = Wuhan ChemFaces Biochemical Co., Ltd. (Wuhan, Hubei, China), E = AK Scientific, Inc. (Union City, CA, USA), F = Ajax Finechem Pvt. Ltd., (Sydney, NSW, Australia), MPA—toluene: ethyl acetate: formic acid (2:8:1, *v*/*v*/*v*), MPB—toluene: ethyl acetate: formic acid (6:5:1, *v*/*v*/*v*).

**Table 2:** Conditions used in performing the HPTLC analysis.

Name	Solvent System	Derivatising Agent
DB-1A	MPA	NP-PEG
DB-1B	MPA	VSA
DB-2A	MPB	NP-PEG
DB-2B	MPB	VSA

**Table 3:** Categories of antioxidant activity for individual bands based on DPPH % RSA.

% DPPH RSA	Category	Inference
0.0%	0	No activity
1.0–33.3%	+	Low activity
33.4–66.6%	++	Medium activity
66.7–100.0%	+++	High activity

**Table 4:** Total phenolic content and antioxidant activity of different Western Australian honeys.

Assay	Honey	Mean	Range	Minimum	Maximum
TPC (mg GAE/100 g)	*Calothamnus* spp. (Red Bell, *n* = 8)	59.4 ± 7.91	27.48	48.09	75.56
	*Agonis flexuosa* (Coastal Peppermint, *n* = 5)	36.08 ± 4.20	12.66	30.05	42.71
	*Corymbia calophylla* (Marri, *n* = 13)	29.15 ± 5.46	17.7	18.91	36.61
	*Eucalyptus marginata* (Jarrah, *n* = 6)	50.58 ± 3.76	9.85	46.24	56.09
FRAP (mmol Fe^2+^/kg)	*Calothamnus* spp. (Red Bell, *n* = 8)	9.24 ± 1.68	4.90	6.76	11.66
	*Agonis flexuosa* (Coastal Peppermint, *n* = 5)	5.45 ± 1.64	4.72	3.69	8.41
	*Corymbia calophylla* (Marri, *n* = 13)	4.48 ± 0.82	3.05	3.47	6.52
	*Eucalyptus marginata* (Jarrah, *n* = 6)	6.83 ± 1.38	3.61	5.20	8.81
DPPH mmol TE/kg	*Calothamnus* spp. (Red Bell, *n* = 8)	3.88 ± 0.96	2.95	2.45	5.41
	*Agonis flexuosa* (Coastal Peppermint, *n* = 5)	2.04 ± 0.57	1.61	1.45	3.06
	*Corymbia calophylla* (Marri, *n* = 13)	1.76 ± 0.58	2.35	1.01	3.36
	*Eucalyptus marginata* (Jarrah, *n* = 6)	2.3 ± 0.76	2.08	1.67	3.75

**Table 5:** Match compounds, correlations, and % similarity of match compounds identified in Western Australian honey (Note: Compound codes are based on a previously published phenolic database paper [30]).

Honey	Data-Base	Rf	Name and Code	Rf	UV DEV	%	UV NP	%	UV VS	%	Match
*Calothamnus* spp. (Red Bell)	1A and 1B	0.630									none
		0.570	Daidzein (**31**)	0.600	0.926	51.2	0.290	24.2	0.841	64.1	Protocatechuic acid
			3,5-DHBA (**39**)	0.594	0.744	23.1	0.476	38.0	0.819	47.0	
			Protocatechuic acid (**55**)	0.577	0.998	100.0	0.978	75.8	0.707	61.2	
			Vanillic acid (**59**)	0.623	0.993	45.5	−0.127	15.4	−0.117	42.7	
		0.455									none
		0.423	EGCG (**29**)	0.407	0.430	57.1	0.711	19.5	0.812	52.7	EGCG
		0.382									none
		0.327									none
		0.299									none
		0.226	Kojic Acid (**105**)	0.287	0.952	39.7	0.583	33.8	−0.072	13.1	Kojic Acid
		0.178									none
		0.110									none
		0.078									none
		0.050									none
		0.020									none
	2A and 2B	0.550	m-Toluic Acid, (**51**)	0.577	0.817	18.7	−0.574	1.2	−0.684	1.99	t-Cinnamic acid
			o-Toluic Acid, (**53**)	0.591	0.791	18.7	0.606	31.5	−0.805	1.99	
			t-Cinnamic acid, (**75**)	0.557	0.963	28.6	−0.376	7.6	0.584	5.98	
		0.515									none
		0.465									none
		0.410									none
		0.335									none
		0.185									none
		0.380	Protocatechuic acid (**55**)	0.377	0.996	65.9	0.987	45.4	0.814	29.1	Protocatechuic acid
		0.270	Gallic acid (**44**)	0.270	0.965	45.0	0.750	40.6	0.580	14.0	Gallic acid
		0.115	Kojic Acid (**105**)	0.130	0.999	50.8	0.779	47.4	0.650	11.1	Kojic Acid
		0.075									none
		0.028									none
*Agonis flexuosa* (Coastal Peppermint)	1A and 1B	0.692									none
		0.615	Benzoic acid (**40**)	0.663	0.892	26.4	0.647	96.7	0.364	11.7	Syringic acid
			Methyl syringate (**48**)	0.610	0.936	44.0	0.829	25.4	0.738	70.5	
			Syringic acid (**58**)	0.577	0.941	45.1	0.859	26.2	0.797	80.8	
			m-Coumaric acid (**67**)	0.633	0.848	39.6	0.921	46.7	−0.806	3.2	
		0.588	Luteolin (**15**)	0.584	0.758	68.1	0.597	34.7	0.331	16.8	Luteolin
		0.500	Epicatechin (**27**)	0.499	0.646	17.6	0.748	31.4	0.284	16.8	Epicatechin
		0.460	Lumichrome (**107**)	0.464	0.837	67.5	0.832	32.7	0.253	20.2	Lumichrome
		0.380	EGCG (**29**)	0.407	0.804	61.5	0.713	19.5	0.442	15.1	EGCG
		0.265	Kojic Acid (**105**)	0.287	0.979	43.7	0.729	29.8	0.715	31.1	Kojic Acid
		0.195									none
		0.140									none
		0.050									none
	2A and 2B	0.475	2,3,4-TMBA (**37**)	0.453	0.883	23.1	0.631	17.1	−0.620	8.3	m-Coumaric acid
			Eudesmic acid (**43**)	0.478	0.970	28.6	0.793	29.5	0.363	3.4	
			Methyl syringate (**48**)	0.471	0.947	40.7	0.805	25.1	0.363	3.4	
			p-Hydroxybenzoic acid (**54**)	0.462	0.784	20.9	0.596	10.0	0.589	14.2	
			m-Coumaric acid (**67**)	0.467	0.866	80.2	0.906	24.3	0.918	7.7	
		0.450									none
		0.415	Syringic acid (**58**)	0.395	0.946	34.1	0.967	24.2	0.739	18.5	Syringic acid
			p-HPAA, HPAAD (**82**)	0.427	0.742	13.2	0.702	11.0	−0.827	19.9	
			DL-p-HPLA, HPLAD (**84**)	0.444	0.777	15.4	0.750	14.3	0.490	29.9	
		0.375	Luteolin (**8**)	0.372	0.911	71.3	0.597	66.5	0.781	28.5	Luteolin
		0.325									none
		0.266	Lumichrome (**107**)	0.266	0.613	63.4	0.638	12.7	0.796	33.3	Lumichrome
		0.235									none
		0.180	Epicatechin (**27**)	0.176	0.727	20.9	0.817	34.3	−0.278	16.8	Epicatechin
		0.105	Kojic Acid (**105**)	0.13	0.962	46.0	0.612	35.1	0.664	46.4	Kojic Acid
		0.090	EGCG (**29**)	0.065	0.852	58.2	0.577	42.2	−0.028	21.7	EGCG
		0.050									none
*Corymbia calophylla* (Marri)	1A and 1B	0.697									none
		0.620	2,3,4-TMBA (**54**)	0.637	0.915	21.3	−0.634	12.9	−0.092	78.3	Eudesmic acid
			Eudesmic acid (**94**)	0.602	0.983	46.7	−0.339	26.7	0.161	32.8	
			p-Hydroxybenzoic acid (**33**)	0.663	0.846	20.5	−0.711	12.9	−0.064	70.1	
		0.600									None
		0.550	Luteolin (**8**)	0.584	0.431	17.1	0.639	90.6	0.500	36.5	Luteolin
		0.475	Epicatechin (**26**)	0.520	0.800	42.9	0.862	12.4	0.765	39.3	Epicatechin
		0.375	EGCG (**29**)	0.407	0.791	79.1	0.679	14.3	0.653	45.6	EGCG
		0.250	Kojic Acid (**105**)	0.241	0.926	98.9	0.711	23.5	−0.061	25.9	Kojic Acid
		0.259									none
		0.216									none
		0.186									none
		0.156									none
		0.115									none
		0.105									none
		0.052									none
		0.025									none
		0.012									none
	2A and 2B	0.470	2,3,4-TMBA (**37**)	0.453	0.977	30.8	0.858	16.8	0.564	11.7	Eudesmic acid
			Eudesmic acid (**43**)	0.478	0.941	65.9	0.981	44.6	0.334	6.0	
			p-HBA (**54**)	0.462	0.955	27.5	0.796	19.8	0.564	14.2	
		0.426	Methyl syringate (**48**)	0.471	0.335	18.7	0.820	22.6	0.211	17.9	m-Coumaric acid
			o-Anisic acid (**52**)	0.440	−0.075	23.1	0.604	16.5	0.644	26.5	
			Syringic acid (**58**)	0.395	0.350	18.7	0.841	23.3	0.751	20.5	
			m-Coumaric acid (**67**)	0.467	0.180	68.1	0.918	42.1	0.758	8.0	
			DL-p-HPLA (**84**)	0.444	0.319	9.9	0.641	11.3	0.601	36.5	
		0.390	Luteolin (**8**)	0.372	0.574	23.9	0.033	31.5	0.887	34.2	Luteolin
		0.346									none
		0.300	Taxifolin (**25**)	0.335	0.758	29.5	0.759	70.1	0.661	8.5	Taxifolin
		0.270	Gallic acid (**44**)	0.270	0.906	71.6	0.655	37.5	0.632	16.5	Gallic acid
		0.186									none
		0.150	Epicatechin (**27**)	0.176	0.774	19.8	0.751	35.7	−0.345	35.9	Epicatechin
		0.110	Kojic Acid (**105**)	0.130	0.960	38.9	0.339	32.5	0.633	27.6	Kojic Acid
*Eucalyptus marginata* (Jarrah)	1A and 1B	0.633	Genistein (**33**)	0.633	0.959	59.6	0.769	31.6	0.432	40.5	2,3,4-THBA
			2,3,4-THBA (**36**)	0.589	0.979	65.1	0.901	76.9	0.900	50.1	
			p-HBA (**54**)	0.637	0.885	24.5	−0.653	8.8	0.707	55.8	
		0.562									none
		0.538									none
		0.471	Lumichrome (**107**)	0.471	0.278	59.7	0.090	18.7	0.592	50.5	Lumichrome
		0.371	EGCG (**29**)	0.371	0.940	41.3	0.165	25.4	0.357	31.0	EGCG
		0.320									none
		0.250	Kojic Acid (**105**)	0.241	0.970	34.1	0.760	44.3	0.128	45.9	Kojic Acid
		0.196									none
		0.117									none
		0.072									none
	2A and 2B	0.525	Hesperetin (**18**)	0.520	0.598	29.8	0.821	40.6	−0.369	32.2	Hesperetin
		0.470	Methyl syringate (**48**)	0.471	0.912	35.5	0.346	36.8	−0.060	16.0	m-Coumaric acid
			m-Coumaric acid (**67**)	0.467	0.944	76.0	0.334	21.8	0.909	7.7	
			p-HPAA (**82**)	0.427	0.709	14.0	0.137	18.0	−0.867	8.8	
			DL-p-HPLA (**84**)	0.444	0.690	14.0	0.627	60.2	−0.722	23.1	
			Phloretic acid (**87**)	0.46	0.704	14.0	0.641	37.6	−0.538	3.1	
		0.420	o-Anisic acid (**52**)	0.44	0.729	25.2	0.689	21.8	0.524	19.9	o-Anisic acid
		0.375									none
		0.320	Taxifolin (**25**)	0.335	0.600	63.9	0.800	71.8	0.661	8.5	Taxifolin
		0.270	Lumichrome (**107**)	0.266	0.136	35.1	0.594	12.7	0.745	64.1	Lumichrome
		0.130	Kojic Acid (**105**)	0.13	0.942	53.4	0.969	82.0	0.695	87.9	Kojic Acid
		0.090	EGCG (**29**)	0.065	0.978	47.7	0.806	49.6	−0.478	14.6	EGCG

**Table 6:** Quantity of Specific Phenolic Constituents (in µg/g, *n* = 3) identified in different Western Australian Honeys.

Compound (see Figure 5 for Structures)	*Calothamnus* spp. (Red Bell)	*Agonis flexuosa* (Coastal Peppermint)	*Corymbia calophylla* (Marri)	*Eucalyptus marginata* (Jarrah)
Luteolin (**1**)	-	1.14 ± 0.00	1.50 ± 0.01	-
Hesperitin (**2**)	-	-	-	0.62 ± 0.01
Taxifolin (**3**)	-	-	1.40 ± 0.01	1.34 ± 0.00
Epicatechin (**4**)	-	6.90 ± 0.03	2.40 ± 0.03	
EGCG (**5**)	3.81 ± 0.01	2.45 ± 0.04	5.11 ± 0.03	5.61 ± 0.04
2,3,4-THBA (**6**)	-	-	-	13.49 ± 0.16
Eudesmic acid (**7**)	-	-	3.25 ± 0.02	-
Gallic acid (**8**)	1.64 ± 0.00	-	5.84 ± 0.00	-
o-Anisic acid (**9**)		-	-	3.52 ± 0.04
Protocatechuic acid (**10**)	5.09 ± 0.02	-	-	-
Syringic acid (**11**)	-	1.47 ± 0.02	-	-
m-Coumaric acid (**12**)	-	0.58 ± 0.02	0.54 ± 0.02	0.72 ± 0.01
t-Cinnamic acid (**13**)	0.003 ± 0.00	-	-	-
Kojic acid (**14**)	3.64 ± 0.00	2.88 ± 0.02	1.32 ± 0.01	0.64 ± 0.01
Lumichrome (**15**)	-	1.94 ± 0.01	-	1.03 ± 0.00

**Table 7:** Colour and % DPPH RSA of various compounds tested for validation of the HPTLC-DPPH analysis.

Sample	Conc. (µg/mL)	H°					%RSA					R^2^
		Volume Application (µL)					Volume Application (µL)					
		5	6.2	7.4	8.6	9.8	5	6.2	7.4	8.6	9.8	
Plate (unreacted DPPH)	0.0	**335.0**	**335.0**	**335.0**	**335.0**	**335.0**	0	0	0	0	0	NA
Luteolin (**1**)	12.5	**347.9**	**358.5**	**3.8**	**11.6**	**15.9**	19.8	36.2	44.3	56.3	62.9	0.979
Epicatechin (**4**)	50.0	**22.7**	**29.2**	**32.3**	**35.6**	**36.2**	73.4	83.4	88.2	93.2	94.2	0.916
Epigallocatechin gallate (**5**)	50.0	**27.0**	**29.3**	**33.2**	**35.0**	**36.5**	80.0	83.5	89.5	92.3	94.6	0.972
Gallic acid (**8**)	25.0	**23.0**	**31.5**	**34.0**	**35.9**	**38.3**	73.8	86.9	90.8	93.7	97.4	0.883
Protocatechuic acid (**10**)	25.0	**28.8**	**32.7**	**34.6**	**34.8**	**35.0**	82.8	88.8	91.7	92.0	92.3	0.768
m-Coumaric acid (**12**)	25.0	**349.9**	**352.6**	**353.9**	**354.1**	**359.2**	22.9	27.1	29.1	29.4	37.2	0.984
Kojic acid (**14**)	25.0	**1.5**	**6.3**	**10.6**	**12.7**	**14.3**	40.8	48.2	54.8	58.0	60.5	0.950

**Table 8:** Percentage DPPH RSA antioxidant activity of individual bands in *Calothamnus* spp. (Red Bell), *Agonis flexuosa* (Coastal Peppermint), *Corymbia calophylla* (Marri) and *Eucalyptus marginata* (Jarrah) honey along with their corresponding matched compounds.

Sample	Rf	Match Compounds	H°	% RSA	Category	% AVE
Baseline	NA	NA	**335.0**	0.0	0	0
Gallic acid (**8**)	NA	NA	**38.3**	97.4	+++	97.4
*Calothamnus* spp. (Red Bell)	0.562	t-CA	**335.0**	0.0	0	33.6
	0.505	-	**342.6**	11.7	+	
	0.450	-	**344.6**	14.8	+	
	0.390	ProA	**37.9**	96.8	+++	
	0.281	GA	**339.1**	6.3	+	
	0.246	-	**337.2**	3.4	+	
	0.207	-	**340.6**	8.6	+	
	0.174	-	**338.7**	5.7	+	
	0.115	KA	**14.5**	60.8	++	
	0.000	-	**36.3**	94.3	+++	
*Agonis flexuosa* (Coastal Peppermint)	0.473	m-CoA	**342.3**	11.2	+	18.4
	0.420	SyrA	**335.0**	0.0	0	
	0.395	Lut	**344.4**	14.5	+	
	0.352	-	**336.9**	2.9	+	
	0.279	Lum	**337.1**	3.2	+	
	0.187	Epi	**337.6**	4.0	+	
	0.114	-	**337.7**	4.2	+	
	0.100	KA	**344.8**	15.1	+	
	0.090	EGCG	**344.8**	15.1	+	
	0.000	-	**37.3**	95.8	+++	
*Corymbia calophylla* (Marri)	0.484	EudA	**336.1**	1.7	+	18.2
	0.444	m-CoA	**340.9**	9.1	+	
	0.391	Lut	**7.6**	50.2	++	
	0.313	Tax	**342.5**	11.5	+	
	0.275	GA	**337.4**	3.7	+	
	0.260	-	**337.2**	3.4	+	
	0.212	-	**337.6**	4.0	+	
	0.179	Epi	**337.6**	4.0	+	
	0.146	-	**339.1**	6.3	+	
	0.105	KA	**343.9**	13.7	+	
	0.00	-	**35.2**	92.6	+++	
*Eucalyptus* *marginata* (Jarrah)	0.530	Hesp	**335.0**	0.0	0	21.1
	0.455	o-AA	**344.3**	14.3	+	
	0.420	m-CoA	**335.0**	0.0	0	
	0.391	-	**8.3**	51.2	++	
	0.322	Tax	**336.1**	1.7	+	
	0.282	Lum	**336.3**	2.0	+	
	0.254	-	**339.1**	6.3	+	
	0.220	-	**336.6**	2.5	+	
	0.189	-	**336.6**	2.5	+	
	0.147	-	**337.6**	4.0	+	
	0.117	-	**339.4**	6.8	+	
	0.100	KA	**351.6**	25.5	+	
	0.083	EGCG	**350.3**	23.5	+	
	0.024	-	**350.8**	24.3	+	
	0.00	-	**32.3**	88.2	+++	

**Table 9:** Colour and % DPPH RSA of matched compounds at a higher and lower application volume (Note: See Table 3 for Antioxidant Category and Inference).

Compound and Code	Sample Applied (ng)	H°	% RSA	Category	Sample Applied (ng)	H°	% RSA	Category	No. of OH	Bors Criteria	Remarks
background (plate)	0	**336.6**	0.0	0	0	**336.6**	0.0	0	NA	NA	NA
Luteolin (**1**)	87.5	**336.7**	2.6	+	350	**20.3**	69.7	++	NA	1	Active
Hesperetin (**2**)	87.5	**334.0**	−1.5	0	700	**356.9**	31.2	+	3	None	Active
Taxifolin (**3**)	35	**340.6**	8.6	+	140	**2.4**	42.2	++	NA	1,3	Active
Epicatechin (**4**)	350	**33.1**	89.4	+++	700	**37.1**	95.5	+++	NA	1	Active
Epigallocatechin gallate (**5**)	350	**28.7**	82.6	+++	700	**36.8**	95.1	+++	NA	1	Active
2,3,4-Trihydroxy benzoic acid (**6**)	175	**345**	15.4	+	700	**28.9**	85.8	+	3	NA	Active
Eudesmic acid (**7**)	350	**333.7**	−2.0	0	1400	**336.6**	0.0	0	0	NA	Inactive
Gallic acid (**8**)	175	**354.1**	29.4	+	700	**38.2**	97.2	+++	3	NA	Active
o-Anisic acid (**9**)	175	**333.7**	−2.0	0	700	**334.5**	−3.2	0	0	NA	Inactive
Protocatechuic acid (**10**)	175	**10.4**	54.5	++	700	**34.6**	91.7	+++	2	NA	Active
Syringic acid (**11**)	175	**340.6**	8.6	+	700	**31.8**	87.4	+++	1	NA	Active
m-Coumaric acid (**12**)	175	**336.6**	2.5	+	700	**352.9**	25.1	+	1	NA	Active
t-Cinnamic acid (**13**)	87.5	**334.0**	−1.5	0	700	**337.2**	0.9	+	0	NA	Inactive
Kojic acid (**14**)	175	**344.6**	14.8	+	700	**11.6**	56.3	++	1	NA	Active
Lumichrome (**15**)	35	**333.7**	−2.0	0	140	**336.6**	0.0	0	0	NA	Inactive

## Data Availability

No new data were created or analysed in this study. Data sharing is not applicable to this article.

## Supplementary Materials

The following supporting information can be downloaded at: https://www.mdpi.com/article/10.3390/antiox12010189/s1, Table S1: Table S1 summarises the identity, botanical origin, and families of honeys collected as part of a study on Western Australia honeys; Table S2: Honey Sample Collection and Floral Information, TPC, FRAP, and DPPH Antioxidant Activity, Table S3: Summary of the data used to determine the identity of the unknown bands in *Calothamnus* spp. (Red Bell) honey (Database 1A), Table S4: Summary of the data used to determine the identity of the unknown bands in *Calothamnus* spp. (Red Bell) honey (Database 1B), Table S5: Summary of the data used to determine the identity of the unknown bands in *Calothamnus* spp. (Red Bell) honey (Database 2A), Table S6: Summary of the data used to determine the identity of the unknown bands in *Calothamnus* spp. (Red Bell) honey (Database 2B), Table S7: Summary of the data used to determine the identity of the unknown bands in *Agonis flexuosa* (Coastal Peppermint) honey (Database 1A), Table S8: Summary of the data used to determine the identity of the unknown bands in *Agonis flexuosa* (Coastal Peppermint) honey (Database 1B), Table S9: Summary of the data used to determine the identity of the unknown bands in *Agonis flexuosa* (Coastal Peppermint) honey (Database 2A), Table S10: Summary of the data used to determine the identity of the unknown bands in *Agonis flexuosa* (Coastal Peppermint) honey (Database 2B), Table S11: Summary of the data used to determine the identity of the unknown bands in *Corymbia calophylla* (Marri) honey (Database 1A), Table S12: Summary of the data used to determine the identity of the unknown bands in *Corymbia calophylla* (Marri) honey (Database 1B), Table S13: Summary of the data used to determine the identity of the unknown bands in *Corymbia calophylla* (Marri) honey (Database 2A), Table S14: Summary of the data used to determine the identity of the unknown bands in *Corymbia calophylla* (Marri) honey (Database 2B), Table S15: Summary of the data used to determine the identity of the unknown bands in *Eucalyptus marginata* (Jarrah) honey (Database 1A), Table S16: Summary of the data used to determine the identity of the unknown bands in *Eucalyptus marginata* (Jarrah) honey (Database 1B), Table S17: Summary of the data used to determine the identity of the unknown bands in *Eucalyptus marginata* (Jarrah) honey (Database 2A), Table S18: Summary of the data used to determine the identity of the unknown bands in *Eucalyptus marginata* (Jarrah) honey (Database 2B); Table S19: Parameters used in optimising the quantification of phenolic compounds in honey, Figure S1. HPTLC fingerprint patterns for various samples of *Calothamnus* spp. (Red bell, *n* = 8), Figure S2. HPTLC fingerprint patterns for various samples of *Agonis flexuosa* (Coastal Peppermint, *n* = 5), Figure S3. HPTLC fingerprint patterns for various samples of *Corymbia calophylla* (Marri, *n* = 13), Figure S4. HPTLC fingerprint patterns for various samples of *Eucalyptus marginata* (Jarrah, *n* = 6), Figure S5A–C. Profile comparison of *Calothamnus* spp. (Red bell) honey (green) and *Calothamnus* spp. (Red bell) honey spiked with the identified compounds based on database 1A and 1B (blue) scanned at the λmax of each specific compounds prior to derivatization, Figure S6A–E. Profile comparison of *Agonis flexuosa* (Coastal Peppermint) Honey (green) and *Agonis flexuosa* (Coastal Peppermint) honey spiked with the identified compounds based on database 1A and 1B (blue) scanned at the λmax of each specific compounds prior to derivatization, Figure S7A–F. Profile comparison of *Agonis flexuosa* (Coastal Peppermint) honey (green) and *Agonis flexuosa* (Coastal Peppermint) honey spiked with the identified compounds based on database 2A and2B (blue) scanned at the λmax of each specific compounds prior to derivatization, Figure S8A–E. Profile comparison of *Corymbia calophylla* (Marri) honey (green) and *Corymbia calophylla* (Marri) spiked with the identified compounds based on database 1A and 1B (blue) scanned at the λmax of each specific compounds prior to derivatization, Figure S9A–G. Profile comparison of *Corymbia calophylla* (Marri) honey (green) and *Corymbia calophylla* (Marri) spiked with the identified compounds based on database 2A and 2B (blue) scanned at the λmax of each specific compounds prior to derivatization, Figure S10A–D. Profile comparison of *Eucalyptus marginata* (Jarrah) honey (green) and *Eucalyptus marginata* (Jarrah) spiked with the identified compounds based on database 1A and 1B (blue) scanned at the λmax of each specific compounds prior to derivatization, Figure S11A–G. Profile comparison of *Eucalyptus marginata* (Jarrah) honey (green) and *Eucalyptus marginata* (Jarrah) spiked with the identified compounds based on database 2A and 2B (blue) scanned at the λmax of each specific compounds prior to derivatization, Figure S12A,B. Comparison of the profiles of compounds identified in *Corymbia calophylla* (Marri) honey after derivatised with DPPH reagent and obtained after 1 h with transmittance in white light (green) vs. scanned at 517 nm (A) and comparison of the profiles of compounds identified in *Corymbia calophylla* (Marri) honey after being derivatised with DPPH reagent scanned at 517 nm and taken at 1 h (green), 2 h (blue), 3 h (grey) (B) developed using mobile phase 1B, Figure S13A–D. HPTLC plate image (a) of *Agonis flexuosa* (Coastal Peppermint) honey after derivatised with DPPH reagent and developed using mobile phase A (A,B) and developed using mobile phase B (C,D) obtained with transmission in white light, and comparison of the profiles of *Agonis flexuosa* (Coastal Peppermint) honey (green) and *Agonis flexuosa* (Coastal Peppermint) honey spiked with the identified compounds (blue) after being derivatised with DPPH reagent obtained at 517 nm (b-left) and comparison of the profiles of *Agonis flexuosa* (Coastal Peppermint) honey obtained at 254 nm (green) and 366 nm (blue) prior to derivatisation and the profile of *Agonis flexuosa* (Coastal Peppermint) honey spiked with the identified compounds (grey) obtained at 277 nm prior to derivatisation (b right), Figure S14A–C. HPTLC plate image (a) of *Corymbia calophylla* (Marri) honey after being derivatised with DPPH reagent and developed using mobile phase A (A) and developed using mobile phase B (B,C) obtained with transmission in white light, and comparison of the profiles of *Corymbia calophylla* (Marri) honey (green) and *Corymbia calophylla* (Marri) honey spiked with the identified compounds (blue) after being derivatised with DPPH reagent obtained at 517 nm (b-left) and comparison of the profiles of *Corymbia calophylla* (Marri) honey obtained at 254 nm (green) and 366 nm (blue) prior to derivatisation and the profile of *Corymbia calophylla* (Marri) honey spiked with the identified compounds (grey) obtained at 277 nm prior to derivatisation (b right), Figure S15A–D. HPTLC plate image (a) of *Eucalyptus marginata* (Jarrah) honey after being derivatised with DPPH reagent and developed using mobile phase A (A and B) and developed using mobile phase B (C,D) obtained with transmission in white light, and comparison of the profiles of *Eucalyptus marginata* (Jarrah) honey (green) and *Eucalyptus marginata* (Jarrah) honey spiked with the identified compounds (blue) after being derivatised with DPPH reagent obtained at 517 nm (b-left) and comparison of the profiles of *Eucalyptus marginata* (Jarrah) honey obtained at 254 nm (green) and 366 nm (blue) prior to derivatisation and the profile of *Eucalyptus marginata* (Jarrah) honey spiked with the identified compounds (grey) obtained at 277 nm prior to derivatisation (b right).

## Sample Availability

Samples of the compounds and honeys are available from the authors.
