# Supplementary material for: Antioxidant Activity and Phenolic Compound Identification and Quantification in Western Australian Honeys
Source: Antioxidants (Basel). 2023 Jan 12;12(1):189. doi: 10.3390/antiox12010189 (PMC9854687; doi:10.3390/antiox12010189)
Supplement: Supplementary file 1 [file antioxidants-12-00189-s001.zip › antioxidants-2087996-supplementary.pdf]

Supplementary Files of "Antioxidant Activity and Phenolic Compound Identification and Quantification in Western Australian Honeys".

Supplementary Table S1: Table S1 summarises the identity, botanical origin, and families of honeys collected as part of a study on Western Australia honeys

Honey	Honey Type	Floral Source	Family	Code	n
Jarrah	Monofloral	*Eucalyptus marginata*	Myrtaceae	JAR	58
Marri	Monofloral	*Corymbia calophylla*	Myrtaceae	MAR	38
Manuka (Control)	Monofloral	*Leptospermum scoparium*	Myrtaceae	MAN	25
Karri	Monofloral	*Eucalyptus diversicolor*	Myrtaceae	KAR	24
Peppermint	Monofloral	*Agonis flexuosa*	Myrtaceae	PEP	24
Leptospermum spp.	Monofloral	*Leptospermum* spp.	Myrtaceae	LEP	19
Parrot Bush	Monofloral	*Banksia sessilis*	Proteaceae	BAS	16
Blackbutt	Monofloral	*Eucalyptus patens*	Myrtaceae	BLA	15
Powderbark	Monofloral	*Eucalyptus accedens*	Myrtaceae	POW	15
Wandoo	Monofloral	*Eucalyptus wandoo*	Myrtaceae	WAN	15
Whitegum	Monofloral	*Eucalyptus wandoo*	Myrtaceae	WHI	14
Yate	Monofloral	*Eucalyptus cornuta*	Myrtaceae	YAT	13
Red Bell	Monofloral	*Calothamnus spp.*	Myrtaceae	RED	12
Banksia menziesii	Monofloral	*Banksia menziesii*	Proteaceae	BAM	10
Mallee	Monofloral	*Eucalyptus* spp.	Myrtaceae	MAL	10
Melaleuca	Monofloral	*Melaleuca* spp.	Myrtaceae	MEL	10
Moort	Monofloral	*Eucalyptus platypus*	Myrtaceae	MOO	10
Blackbutt Coastal	Monofloral	*Eucalyptus* spp.	Myrtaceae	BLC	9
Eucalyptus spp.	Monofloral	*Eucalyptus* spp.	Myrtaceae	EUC	9
Multifloral	Multifloral	N.I.	N.I.	MUL	9
Callistemon	Monofloral	*Callistemon* spp.	Myrtaceae	CAL	8
Goldfields	Multifloral	N.I.	N.I.	GOL	8
Wildflower	Multifloral	N.I.	N.I.	OH	6
Banksia	Monofloral	*Banksia* spp.	Proteaceae	OH	5
Brown Mallet	Monofloral	*Eucalyptus astringens*	Myrtaceae	OH	5
Bloodwood	Monofloral	*Corymbia zygophylla*	Myrtaceae	OH	4
Spring	Multifloral	N.I.	N.I.	OH	4
Watermelon	Monofloral	*Citrullus lanatus*	Cucurbitaceae	OH	4
Canola	Monofloral	*Brassica napus*	Brassicaceae	OH	3
Coastal	Multifloral	N.I.	N.I.	OH	3
Eremophila	Monofloral	*Eremophila* spp.	Scrophulariaceae	OH	3
Flooded Gum	Monofloral	*Eucalyptus rudis*	Myrtaceae	OH	3
Grevillea	Monofloral	*Grevillea* spp.	Proteaceae	OH	3
Merrit	Monofloral	*Eucalyptus flocktoniae*	Myrtaceae	OH	3
Tagasaste	Monofloral	*Cytisus proliferus*	Fabaceae	OH	3
Tedera	Monofloral	*Bituminaria bituminosa*	Fabaceae	OH	3
Tuart	Monofloral	*Eucalyptus gomphocephala*	Myrtaceae	OH	3
York Gum	Monofloral	*Eucalyptus loxophleba*	Myrtaceae	OH	3

Lophostemon	Monofloral	*Lophostemon confertus*	Myrtaceae	OH	2
Scholtzia	Monofloral	*Scholtzia* spp.	Myrtaceae	OH	2
Acacia	Monofloral	*Acacia* spp.	Fabaceae	OH	1
Almond	Monofloral	*Prunus dulcis*	Rosaceae	OH	1
Banksia grandis	Monofloral	*Banksia grandis*	Proteaceae	OH	1
Banksia prionotes	Monofloral	*Banksia prionotes*	Proteaceae	OH	1
Banksia victoriae	Monofloral	*Banksia victoriae*	Proteaceae	OH	1
Blackbutt Goldfields	Monofloral	*Eucalyptus* spp.	Myrtaceae	OH	1
Bullich	Monofloral	*Eucalyptus megacarpa*	Myrtaceae	OH	1
Capeweed	Monofloral	*Arctotheca calendula*	Asteraceae	OH	1
Chamelaucium	Monofloral	*Chamelaucium spp.*	Myrtaceae	OH	1
Christmas Tree	Monofloral	*Nuytsia* spp.	Loranthaceae	OH	1
Clivicola	Monofloral	*Eucalyptus clivicola*	Myrtaceae	OH	1
Field Pea	Monofloral	*Pisum sativum*	Fabaceae	OH	1
Gimlet	Monofloral	*Eucalyptus salubris*	Myrtaceae	OH	1
Orange Blossum	Monofloral	*Citrus* spp.	Rutaceae	OH	1
Spotted Gum	Monofloral	*Corymbia maculata*	Myrtaceae	OH	1
Taxandria	Monofloral	*Taxandria* spp.	Myrtaceae	OH	1
Tingle	Monofloral	*Eucalyptus jacksonii*	Myrtaceae	OH	1
Pasteur (Control)	Multifloral	N.I.	N.I.	OH	1
no data	N.I.	N.I.	N.I.	ND	11

Supplementary Table S2: Honey Sample Collection and Floral Information, TPC, FRAP, and DPPH Antioxidant Activity

Code	Date Collected	Location	Post Code	Floral Source	TPC (GA eq.mg /100g)	SD	RSD (%)	FRAP (mmol Fe/kg)	SD	RSD (%)	DPPH (mmol TE /kg at 2 hrs)	SD	RSD (%)
RED-86	01-Dec-17	Cervantes	6511	*Calothamnus* spp.	59.63	0.440	0.738	9.75	0.21	2.11	3.67	0.06	1.77
RED-95	01-Dec-17	Cervantes	6511	*Calothamnus* spp.	61.47	0.398	0.648	10.25	0.26	2.54	4.60	0.09	2.01
RED-101	01-Nov-17	Cervantes	6511	*Calothamnus* spp.	54.58	0.830	1.520	7.79	0.19	2.39	3.07	0.06	1.82
RED-102	01-Nov-17	Seabird	6042	*Calothamnus* spp.	48.09	0.593	1.232	6.76	0.19	2.74	2.45	0.14	5.54
RED-103	01-Dec-17	Seabird	6042	*Calothamnus* spp.	56.52	0.136	0.240	9.36	0.15	1.60	3.93	0.07	1.67
RED-106	01-Nov-17	Cervantes	6511	*Calothamnus* spp.	53.65	0.836	1.559	7.31	0.23	3.15	3.02	0.11	3.59
RED-134	NI	NI	NI	*Calothamnus* spp.	65.70	0.959	1.459	11.02	0.14	1.26	4.85	0.07	1.52
RED-166	NI	NI	NI	*Calothamnus* spp.	75.56	0.262	0.347	11.66	0.06	0.47	5.41	0.09	1.67
PEP-191	10-Jan-19	Quedjinup	6281	*Agonis flexuosa*	37.29	0.917	2.458	4.41	0.05	1.19	1.98	0.07	3.55
PEP-240	01-Nov-17	Yalgorup	6215	*Agonis flexuosa*	33.94	0.187	0.552	3.69	0.20	5.29	1.45	0.08	5.40
PEP-260	NI	Yallingup	6282	*Agonis flexuosa*	42.71	0.520	1.217	8.41	0.16	1.91	3.06	0.05	1.60
PEP-264	NI	NI	NI	*Agonis flexuosa*	36.40	0.641	1.761	5.81	0.19	3.29	2.06	0.14	7.03
PEP-310	01-Jan-18	Albany	6330	*Agonis flexuosa*	30.05	0.511	1.699	4.92	0.06	1.18	1.63	0.12	7.38
MAR-53.	28-Mar-18	Bindoon	6502	*Corymbia calophylla*	32.04	0.363	1.132	4.61	0.02	0.46	1.50	0.06	3.70
MAR-54.	28-Mar-18	Bindoon	6502	*Corymbia calophylla*	30.52	0.336	1.100	3.84	0.06	1.51	1.01	0.08	8.31
MAR-117	2018	Mooliabeenie	6504	*Corymbia calophylla*	33.62	0.563	1.673	4.40	0.08	1.73	1.77	0.07	3.69
MAR-118	2018	Mooliabeenie	6504	*Corymbia calophylla*	34.19	0.488	1.427	4.50	0.28	6.24	1.72	0.11	6.59
MAR-120	NI	NI	NI	*Corymbia calophylla*	36.61	0.027	0.075	4.51	0.16	3.50	1.64	0.12	7.35
MAR-121	NI	NI	NI	*Corymbia calophylla*	32.15	0.327	1.017	3.86	0.22	5.61	1.79	0.06	3.31
MAR-309	01-Mar-19	Gingin	6503	*Corymbia calophylla*	31.55	1.216	3.853	5.86	0.21	3.64	2.16	0.16	7.62
MAR-315	08-Feb-20	Harvey	6220	*Corymbia calophylla*	20.05	0.242	1.206	3.86	0.03	0.88	1.26	0.05	4.08
MAR-316	23-Feb-20	Harvey	6220	*Corymbia calophylla*	18.91	0.196	1.035	3.47	0.02	0.67	1.11	0.08	7.08
MAR-322	NI	Gidgegannup	6083	*Corymbia calophylla*	24.50	0.209	0.851	4.41	0.05	1.14	2.16	0.18	8.31
MAR-323	NI	Allanson	6225	*Corymbia calophylla*	26.43	0.324	1.225	4.43	0.08	1.76	1.77	0.06	3.58
MAR-325	NI	NI	NI	*Corymbia calophylla*	33.73	0.367	1.089	6.52	0.09	1.44	3.36	0.06	1.69

MAR-329	17-Feb-20	Yarloop	6218	*Corymbia calophylla*	24.63	0.377	1.530	3.96	0.18	4.66	1.67	0.05	3.00
JAR-09	01-Nov-15	Yarloop	6218	*Eucalyptus marginata*	56.09	1.254	2.236	8.25	0.21	2.57	2.83	0.09	3.33
JAR-50	NI	Dwellingup	6213	*Eucalyptus marginata*	54.43	1.205	2.214	8.81	0.13	1.53	3.75	0.07	1.93
JAR-68	06-Jan-17	Jarrahdale	6124	*Eucalyptus marginata*	47.88	0.175	0.366	6.30	0.11	1.74	1.79	0.07	4.13
JAR-69	06-Jan-17	Jarrahdale	6124	*Eucalyptus marginata*	47.54	0.378	0.794	5.34	0.35	6.63	1.67	0.03	1.91
JAR-75	08-Dec-16	Jarrahdale	6124	*Eucalyptus marginata*	51.31	0.584	1.139	7.11	0.18	2.49	1.98	0.11	5.79
JAR-78	NI	NI	NI	*Eucalyptus marginata*	46.24	0.278	0.602	5.20	0.19	3.72	1.82	0.10	5.23

Supplementary Table S3: Summary of the data used to determine the identity of the unknown bands in *Calothamnus* spp. (Red Bell) honey (Database 1A)

Name and Code	Rf 1	H° DEV 254 nm	H° DEV 366 nm	H° NP 366 nm	Fl DEV λ	Fl DEV λ m	UV DEV λ1	UV DEV λ2	UV DEV λ3	Fl NP λ	UV NP λ1
RB	0.035	140.8	197.1	195.6	223	260	273	0	0	249	299
RB	0.055	139.9	193.8	192.6	223	263	283	0	0	249	322
RB	0.078	140.0	193.0	192.2	225	260	287	0	0	250	319
RB	0.11	140.3	196.9	194.1	225	261	288	0	0	250	324
RB	0.178	139.8	193.9	191.4	222	258	282	0	0	250	315
RB	0.226	139.5	190.3	188.9	210	258	272	0	0	250	313
RB	0.299	141	189.4	191.6	223	258	289	0	0	250	319
RB	0.327	140.3	191.3	193.9	225	258	286	0	0	250	317
RB	0.382	139.9	191.0	194.7	223	254	287	0	0	250	294
RB	0.423	139.0	191.0	199.9	222	254	287	0	0	249	299
RB	0.455	140.4	190.8	199.8	224	261	287	0	0	250	312
RB	0.576	139.5	195.2	196.2	224	255	262	296	0	245	328
RB	0.609	139.3	202.0	200.2	225	255	261	292	0	246	329

Legend: **Rf1** – retention factor in MPA, **H° DEV 254 nm** – hue equivalent at 254 nm prior to derivatisation, **H° DEV 366 nm** – hue equivalent at 366 nm prior to derivatisation, **H° NP 366 nm** – hue equivalent at 366 nm after derivatisation w/ NP-PEG derivatisation reagent, **Fl DEV λ**– fluorescence λ max prior to derivatisation, **Fl DEV λ m** – fluorescence λ min prior to derivatisation, **UV DEV $λ_{1-3}$** – UV-Vis λ max prior to derivatisation, **Fl NP λ**– fluorescence λ max after derivatisation with NP-PEG reagent, **UV NP $λ_{1-3}$**– UV-Vis λ max after derivatisation with NP-PEG reagent, Note: coloured cells represent colours as seen on HPTLC plate.

Supplementary Table S4: Summary of the data used to determine the identity of the unknown bands in *Calothamnus* spp. (Red Bell) honey (Database 1B)

Name and Code	Rf 1	H° DEV 254 nm	H° DEV 366 nm	H° VSA 366 nm	H° T VSA	Fl DEV λ	Fl DEV λ m	UV DEV λ_1	UV DEV λ_2	UV DEV λ_3	Fl VS λ	UV VS λ
RB	0.035	139.7	196.3	205.2	24.6	223	260	272	0	0	248	377
RB	0.055	140.0	192.3	205.1	25.3	223	263	287	0	0	254	454
RB	0.078	140.3	193.3	203.4	26.0	223	260	286	0	0	254	454
RB	0.11	139.7	198.3	210.5	18.1	223	261	289	0	0	246	458
RB	0.178	139.8	193.1	205.1	21.6	222	258	283	0	0	249	450
RB	0.226	139.5	191.1	202.2	28.2	222	258	276	0	0	248	447
RB	0.299	139.7	189.4	202.2	25	225	258	288	0	0	249	472
RB	0.327	139.7	190.2	209.2	14.5	223	258	287	0	0	249	472
RB	0.382	140.0	191.0	206.2	21.5	225	254	287	0	0	249	472
RB	0.423	139.1	190.2	208.5	22.7	223	254	287	0	0	248	461
RB	0.455	140.4	190.5	210.8	26.1	223	261	287	0	0	249	464
RB	0.576	139.1	195.2	212.7	2.4	225	255	261	295	0	249	453
RB	0.609	139.5	202.0	219.1	204.6	225	255	262	293	0	249	461

Legend: **Rf1** – retention factor in **MPA**, **H° DEV 254 nm** – hue and colour equivalent at 254 nm prior to derivatisation, **H° DEV 366 nm** – hue and colour equivalent at 366 nm prior to derivatisation, **H° VS 366 nm** – hue and colour equivalent at 366 nm after derivatisation w/ VSA derivatisation reagent, **H° T VS** – hue and colour equivalent at transmittance in white light after derivatisation w/ VSA derivatisation reagent; **Fl DEV λ max**– fluorescence λ max prior to derivatisation, **Fl DEV λ m**– fluorescence λ min prior to derivatisation, **UV DEV $\lambda_{1\text{-}3}$** – UV-Vis λ max prior to derivatisation, **Fl VS λ**– fluorescence λ max after derivatisation with VSA reagent, **UV VS λ**–– UV-Vis λ max after derivatisation with VSA reagent, Note: coloured cells represent colours as seen on HPTLC plate.

Supplementary Table S5: Summary of the data used to determine the identity of the unknown bands in *Calothamnus* spp. (Red Bell) honey (Database 2A)

Name and Code	Rf 2	H° DEV 254 nm	H° DEV 366 nm	H° NP 366 nm	Fl DEV λ	Fl DEV λ m	UV DEV λ_1	UV DEV λ_2	UV DEV λ_3	Fl NP λ	UV NP λ1
RB	0.040	139.6	198.3	193.6	224	256	277	0	0	246	321
RB	0.061	139.8	196.7	188.9	225	256	277	0	0	246	317
RB	0.087	139.7	194.7	190.3	221	252	279	0	0	245	316
RB	0.130	138.8	189.3	192.5	221	252	277	0	0	245	314
RB	0.220	140.5	188.0	205.9	221	254	285	0	0	246	322
RB	0.252	139.7	190.2	195.6	219	259	282	0	0	244	296
RB	0.290	140.2	189.4	83.0	221	252	286	0	0	243	315
RB	0.305	140.7	189.3	83.0	223	252	286	0	0	243	315
RB	0.355	139.9	191.4	196.3	221	259	286	0	0	244	314
RB	0.385	139.5	192.8	207.5	224	255	261	295	0	246	327
RB	0.404	137.9	192.2	197.3	225	256	262	295	0	244	328
RB	0.435	139.8	191.4	207.1	225	243	262	294	0	244	328
RB	0.489	139.6	196.5	197.5	227	253	261	295	0	243	322
RB	0.513	140.0	201.2	190.4	226	255	261	0	0	243	304
RB	0.567	140.8	189.3	194.9	226	258	284	0	0	246	338

Legend: **Rf2** – retention factor in MPB, **H° DEV 254 nm** – hue equivalent at 254 nm prior to derivatisation, **H° DEV 366 nm** – hue equivalent at 366 nm prior to derivatisation, **H° NP 366 nm** – hue equivalent at 366 nm after derivatisation w/ NP-PEG derivatisation reagent, **Fl DEV λ**– fluorescence λ max prior to derivatisation, **Fl DEV λ m** – fluorescence λ min prior to derivatisation, **UV DEV $\lambda_{1\text{-}3}$** – UV-Vis λ max prior to derivatisation, **Fl NP λ**– fluorescence λ max after derivatisation with NP-PEG reagent, **UV NP $\lambda_{1\text{-}3}$**– UV-Vis λ max after derivatisation with NP-PEG reagent, Note: coloured cells represent colours as seen on HPTLC plate.

Supplementary Table S6: Summary of the data used to determine the identity of the unknown bands in *Calothamnus* spp. (Red Bell) honey (Database 2B)

Name and Code	Rf 2	H° DEV 254 nm	H° DEV 366 nm	H° VSA 366 nm	H° T VSA	Fl DEV λ	Fl DEV λ m	UV DEV λ_1	UV DEV λ_2	UV DEV λ_3	Fl VS λ	UV VS λ
RB	0.04	139.9	201.9	213.0	19.1	224	256	277	0	0	253	451
RB	0.061	139.7	196.9	205.3	19.5	225	256	277	0	0	250	451
RB	0.087	139.3	194.7	206.2	18.2	221	252	279	0	0	247	453
RB	0.13	138.6	189.2	206.2	21.3	221	252	277	0	0	248	452
RB	0.22	139.8	188.9	212.1	12.0	221	254	285	0	0	251	473
RB	0.252	139.0	190.6	211.6	12.0	219	259	282	0	0	248	477
RB	0.29	140.1	189.3	214.1	23.4	221	252	286	0	0	248	458
RB	0.305	140.1	192.2	211.2	16.5	223	252	286	0	0	252	464
RB	0.355	140.2	191.7	215.3	358.1	221	259	286	0	0	248	373
RB	0.385	139.2	191.8	232.7	337.3	224	255	261	295	0	252	499
RB	0.404	138.6	192.2	244.7	280.0	225	256	262	295	0	250	499
RB	0.450	139.7	192.8	225.0	213.1	225	243	262	294	0	252	498
RB	0.5	139.8	199.3	210.5	2.3	227	253	267	0	0	248	353
RB	0.513	139.7	199.4	209.1	7.3	226	255	261	0	0	250	481

Legend: **Rf2** – retention factor in **MPB**, **H° DEV 254 nm** – hue and colour equivalent at 254 nm prior to derivatisation, **H° DEV 366 nm** – hue and colour equivalent at 366 nm prior to derivatisation, **H° VSA 366 nm** – hue and colour equivalent at 366 nm after derivatisation w/ VSA derivatisation reagent, **H° T VSA** – hue and colour equivalent at transmittance in white light after derivatisation w/ VSA derivatisation reagent; **Fl DEV λ max**– fluorescence λ max prior to derivatisation, **Fl DEV λ m**– fluorescence λ min prior to derivatisation, **UV DEV $\lambda_{1\text{-}3}$** – UV-Vis λ max prior to derivatisation, **Fl VS λ**– fluorescence λ max after derivatisation with VSA reagent, **UV VS λ**— UV-Vis λ max after derivatisation with VSA reagent, Note: coloured cells represent colours as seen on HPTLC plate.

Supplementary Table S7: Summary of the data used to determine the identity of the unknown bands in *Agonis flexuosa* (Coastal Peppermint) honey (Database 1A)

Name and Code	Rf 1	H° DEV 254 nm	H° DEV 366 nm	H° NP 366 nm	Fl DEV λ	Fl DEV λ m	UV DEV λ_1	UV DEV λ_2	UV DEV λ_3	Fl NP λ	UV NP λ1	UV NP λ2
CP	0.008	127.5	194.1	187.3	223	251	288	0	0	242	298	385
CP	0.019	139.5	198.1	191.4	223	252	287	0	0	243	298	385
CP	0.038	139.7	199.3	192.0	223	253	291	0	0	249	300	400
CP	0.075	138.4	200.0	192.0	223	251	287	0	0	249	296	395
CP	0.134	138.9	166.4	188.2	224	251	280	0	0	249	312	401
CP	0.195	138.8	196.5	194.9	223	241	317	0	0	249	319	398
CP	0.241	138.9	189.6	192.1	224	251	296	0	0	249	315	400
CP	0.261	138.9	191.9	192.2	224	251	287	0	0	251	315	400
CP	0.314	139.1	192.1	192.2	225	251	286	0	0	249	313	400
CP	0.351	138.2	190.6	191.0	224	248	287	0	0	249	296	398
CP	0.388	138.0	192.9	192.9	221	250	286	0	0	248	296	402
CP	0.471	149.6	190.1	195.3	224	252	262	357	0	243	298	393
CP	0.499	140.3	191.3	194.7	221	248	287	0	0	244	298	393
CP	0.545	139.1	191.3	82.4	224	249	268	352	0	244	296	398
CP	0.563	138.0	191.3	82.4	223	248	268	352	0	244	298	398
CP	0.588	137.8	191.3	82.4	224	251	260	0	0	243	291	468
CP	0.615	137.7	201.2	190.6	225	253	272	0	0	244	291	398
CP	0.690	139.4	191.7	190.4	225	253	285	0	0	249	295	398

Legend: **Rf1** – retention factor in MPA, **H° DEV 254 nm** – hue equivalent at 254 nm prior to derivatisation, **H° DEV 366 nm** – hue equivalent at 366 nm prior to derivatisation, **H° NP 366 nm** – hue equivalent at 366 nm after derivatisation w/ NP-PEG derivatisation reagent, **Fl DEV λ**– fluorescence λ max prior to derivatisation, **Fl DEV λ m** – fluorescence λ min prior to derivatisation, **UV DEV $\lambda_{1\text{-}3}$** – UV-Vis λ max prior to derivatisation, **Fl NP λ**– fluorescence λ max after derivatisation with NP-PEG reagent, **UV NP $\lambda_{1\text{-}3}$**– UV-Vis λ max after derivatisation with NP-PEG reagent, Note: coloured cells represent colours as seen on HPTLC plate.

Supplementary Table S8: Summary of the data used to determine the identity of the unknown bands in *Agonis flexuosa* (Coastal Peppermint) honey (Database 1B)

Name and Code	Rf 2	H° DEV 254 nm	H° DEV 366 nm	H° VSA 366 nm	H° T VSA	Fl DEV λ	Fl DEV λ m	UV DEV λ_1	UV DEV λ_2	UV DEV λ_3	Fl VS λ	UV VS λ
CP	0.035	139.7	196.3	205.2	24.6	223	258	272	0	0	248	377
CP	0.055	140.0	192.3	205.1	25.3	223	261	287	0	0	254	454
CP	0.078	140.3	193.3	203.4	26.0	223	261	286	0	0	254	454
CP	0.110	139.7	198.3	210.5	18.1	223	262	289	0	0	246	458
CP	0.178	139.8	193.1	205.1	21.6	222	260	283	0	0	249	450
CP	0.226	139.5	191.1	202.2	28.2	222	258	276	0	0	248	447
CP	0.299	139.7	189.4	248.0	25.0	225	260	288	0	0	249	472
CP	0.327	139.7	190.2	209.2	14.5	223	258	287	0	0	249	472
CP	0.382	140.0	191.0	206.2	21.5	225	261	287	0	0	249	472
CP	0.423	139.1	190.2	208.5	22.7	223	253	282	0	0	248	461
CP	0.455	140.4	190.5	210.8	26.1	223	260	287	0	0	249	464
CP	0.576	139.1	195.2	212.7	2.4	225	255	261	295	0	249	453
CP	0.609	139.5	202.0	219.1	204.6	225	256	262	293	0	249	461

Legend: **Rf1** – retention factor in **MPB**, **H° DEV 254 nm** – hue and colour equivalent at 254 nm prior to derivatisation, **H° DEV 366 nm** – hue and colour equivalent at 366 nm prior to derivatisation, **H° VSA 366 nm** – hue and colour equivalent at 366 nm after derivatisation w/ VSA derivatisation reagent, **H° T VSA** – hue and colour equivalent at transmittance in white light after derivatisation w/ VSA derivatisation reagent; **Fl DEV λ max**– fluorescence λ max prior to derivatisation, **Fl DEV λ m**– fluorescence λ min prior to derivatisation, **UV DEV λ_{1-3}** – UV-Vis λ max prior to derivatisation, **Fl VS λ**– fluorescence λ max after derivatisation with VSA reagent, **UV VS λ**— UV-Vis λ max after derivatisation with VSA reagent, Note: coloured cells represent colours as seen on HPTLC plate.

Supplementary Table S9: Summary of the data used to determine the identity of the unknown bands in *Agonis flexuosa* (Coastal Peppermint) honey (Database 2A)

Name and Code	Rf 1	H° DEV 254 nm	H° DEV 366 nm	H° NP 366 nm	Fl DEV λ	Fl DEV λ m	UV DEV λ1	UV DEV λ2	UV DEV λ3	Fl NP λ	UV NP λ1	UV NP λ2
CP	0.020	142.0	201.0	180.3	223	251	292	0	0	240	268	0
CP	0.055	142.0	198.0	194.0	221	242	317	0	0	233	291	0
CP	0.090	141.0	196.0	193.9	220	255	293	0	0	236	269	291
CP	0.105	141.0	193.0	192.6	220	255	292	0	0	239	269	291
CP	0.135	141.0	193.0	193.8	218	246	286	0	0	237	269	291
CP	0.180	140.0	194.0	194.9	223	250	286	0	0	236	269	291
CP	0.235	140.0	194.0	194.7	222	255	286	0	0	237	269	291
CP	0.265	149.0	193.0	194.3	223	254	263	356	0	238	269	291
CP	0.285	147.0	194.0	193.1	223	255	263	356	0	239	269	291
CP	0.325	139.0	194.0	186.4	222	247	287	0	0	236	269	291
CP	0.355	139.0	198.0	145.0	222	247	283	0	0	236	269	291
CP	0.375	141.0	196.0	102.1	222	248	266	350	0	242	269	291
CP	0.405	140.0	194.0	152.5	225	252	266	350	0	240	269	291
CP	0.415	140.0	192.0	185.3	222	252	280	0	0	239	269	291
CP	0.450	140.0	197.0	193.1	223	235	280	0	0	242	269	291
CP	0.475	140.0	200.0	193.0	227	258	270	0	0	242	269	291
CP	0.550	140.0	192.0	196.0	226	257	285	0	0	242	269	291
CP	0.600	140.0	188.0	194.6	228	266	294	0	0	248	269	291

Legend: **Rf2** – retention factor in MPA, **H° DEV 254 nm** – hue equivalent at 254 nm prior to derivatisation, **H° DEV 366 nm** – hue equivalent at 366 nm prior to derivatisation, **H° NP 366 nm** – hue equivalent at 366 nm after derivatisation w/ NP-PEG derivatisation reagent, **Fl DEV λ**– fluorescence λ max prior to derivatisation, **Fl DEV λ m** – fluorescence λ min prior to derivatisation, **UV DEV $\lambda_{1\text{-}3}$** – UV-Vis λ max prior to derivatisation, **Fl NP λ**– fluorescence λ max after derivatisation with NP-PEG reagent, **UV NP $\lambda_{1\text{-}3}$**– UV-Vis λ max after derivatisation with NP-PEG reagent, Note: coloured cells represent colours as seen on HPTLC plate.

Supplementary Table S10: Summary of the data used to determine the identity of the unknown bands in *Agonis flexuosa* (Coastal Peppermint) honey (Database 2B)

Name and Code	Rf 2	H° DEV 254 nm	H° DEV 366 nm	H° VSA 366 nm	H° T VSA	Fl DEV λ	Fl DEV λ m	UV DEV λ_1	UV DEV λ_2	UV DEV λ_3	Fl VS λ	UV VS λ
CP	0.030	141.6	201.0	207.5	11.7	223	251	316	0	0	261	386
CP	0.050	141.2	195.5	203.8	10.6	223	242	316	0	0	258	386
CP	0.060	141.6	191.3	200.6	12.6	221	242	317	0	0	251	386
CP	0.075	141.1	188.9	203.2	12.7	222	242	280	0	0	258	386
CP	0.105	141.3	190.9	202.5	7.6	220	255	289	0	0	252	386
CP	0.130	140.5	194.1	203.2	7.6	218	246	289	0	0	251	386
CP	0.150	140.5	187.4	206.1	11.9	218	244	288	0	0	248	386
CP	0.190	139.9	192.0	211.6	327.0	223	250	287	0	0	251	386
CP	0.245	140.2	190.3	207.7	358.1	222	255	280	0	0	248	388
CP	0.275	147.5	192.3	190.3	21.6	223	254	262	357	0	275	388
CP	0.290	147.0	192.1	192.1	35.2	223	255	264	356	0	252	388
CP	0.340	138.6	192.8	241.6	240.0	222	247	288	0	0	259	388
CP	0.370	138.6	197.5	218.8	336.9	222	248	258	349	0	258	386
CP	0.390	140.7	195.9	202.2	2.0	222	248	258	349	0	251	384
CP	0.420	140.6	193.0	219.6	3.1	225	252	280	0	0	250	385
CP	0.430	140.1	192.6	272.7	2.4	222	252	280	0	0	248	387
CP	0.455	140.3	197.5	334.0	2.0	223	235	279	0	0	248	450
CP	0.485	140.0	196.6	204.8	13.3	227	258	266	0	0	248	387
CP	0.560	140.0	190.9	201.5	8.9	226	257	285	0	0	248	387
CP	0.600	140.3	189.4	201.2	346.7	225	266	292	0	0	249	387

Legend: **Rf2** – retention factor in **MPB**, **H° DEV 254 nm** – hue and colour equivalent at 254 nm prior to derivatisation, **H° DEV 366 nm** – hue and colour equivalent at 366 nm prior to derivatisation, **H° VSA 366 nm** – hue and colour equivalent at 366 nm after derivatisation w/ VSA derivatisation reagent, **H° T VSA** – hue and colour equivalent at transmittance in white light after derivatisation w/ VSA derivatisation reagent; **Fl DEV λ max**– fluorescence λ max prior to derivatisation, **Fl DEV λ m**– fluorescence λ min prior to derivatisation, **UV DEV $\lambda_{1\text{-}3}$** – UV-Vis λ max prior to derivatisation, **Fl VS λ**– fluorescence λ max after derivatisation with VSA reagent, **UV VS λ**— UV-Vis λ max after derivatisation with VSA reagent, Note: coloured cells represent colours as seen on HPTLC plate.

Supplementary Table S11: Summary of the data used to determine the identity of the unknown bands in *Corymbia calophylla* (Marri) honey (Database 1A)

Name and Code	Rf 1	H° DEV 254 nm	H° DEV 366 nm	H° NP 366 nm	Fl DEV λ	Fl DEV λ m	UV DEV λ_1	UV DEV λ_2	UV DEV λ_3	Fl NP λ	UV NP λ1	UV NP λ2
Marri	0.012	141.5	199.4	180.0	223	258	292	0	0	249	299	0
Marri	0.025	140.7	200.0	193.9	223	258	290	0	0	250	299	0
Marri	0.052	139.7	194.0	191.5	223	258	287	0	0	248	299	0
Marri	0.105	139.7	194.2	189.4	223	253	255	291	0	250	299	0
Marri	0.115	140.0	193.2	187.8	223	252	255	291	0	250	299	0
Marri	0.156	139.6	193.0	186.4	223	255	258	291	0	250	317	0
Marri	0.186	140.2	194.0	186.0	223	258	287	0	0	250	314	0
Marri	0.216	140.5	195.5	177.2	222	258	287	0	0	250	313	0
Marri	0.259	140.7	197.8	184.6	223	255	288	361	0	250	310	0
Marri	0.294	140.1	193.9	186.2	223	258	288	0	0	250	299	0
Marri	0.375	140.1	190.5	191.9	222	250	287	0	0	249	294	0
Marri	0.455	140.1	191.1	185.8	223	252	286	0	0	246	301	0
Marri	0.475	139.3	192.5	165.4	223	252	286	0	0	247	304	0
Marri	0.499	139.2	195.1	102.5	223	250	287	0	0	247	299	0
Marri	0.546	133.9	190.4	53.6	222	249	291	0	0	236	294	0
Marri	0.609	139.3	194.2	110.2	222	249	291	0	0	236	294	0
Marri	0.619	139.3	194.0	144.0	225	254	258	0	0	245	294	0
Marri	0.697	140.2	186.2	190.4	225	254	288	0	0	245	335	0

Legend: **Rf1** – retention factor in MPA, **H° DEV 254 nm** – hue equivalent at 254 nm prior to derivatisation, **H° DEV 366 nm** – hue equivalent at 366 nm prior to derivatisation, **H° NP 366 nm** – hue equivalent at 366 nm after derivatisation w/ NP-PEG derivatisation reagent, **Fl DEV λ**– fluorescence λ max prior to derivatisation, **Fl DEV λ m** – fluorescence λ min prior to derivatisation, **UV DEV** λ_{1-3} – UV-Vis λ max prior to derivatisation, **Fl NP λ**– fluorescence λ max after derivatisation with NP-PEG reagent, **UV NP** λ_{1-3}– UV-Vis λ max after derivatisation with NP-PEG reagent

Supplementary Table S12: Summary of the data used to determine the identity of the unknown bands in *Corymbia calophylla* (Marri) honey (Database 1B)

**Name and Code**	**Rf 1**	**H° DEV 254 nm**	**H° DEV 366 nm**	**H° VSA 366 nm**	**H° T VSA**	**Fl DEV λ**	**Fl DEV λ m**	**UV DEV λ_1**	**UV DEV λ_2**	**UV DEV λ_3**	**Fl VS λ**	**UV VS λ**
Marri	0.055	139.7	197.0	204.0	12.4	220	255	289	0	0	258	385
Marri	0.080	140.3	196.0	201.0	13.9	209	257	289	0	0	250	384
Marri	0.095	140.3	196.0	202.0	13.9	213	257	289	0	0	248	385
Marri	0.115	139.5	193.0	202.0	11.5	213	257	289	0	0	245	387
Marri	0.135	139.9	195.0	201.0	12.5	213	247	289	0	0	248	386
Marri	0.145	139.3	194.0	201.0	14.6	213	247	288	0	0	248	388
Marri	0.165	139.9	191.0	204.0	8.9	213	254	288	0	0	247	386
Marri	0.195	140.0	188.0	211.0	7.8	213	252	288	0	0	254	385
Marri	0.245	139.1	182.0	208.0	16.1	214	252	256	0	0	250	386
Marri	0.285	140.3	192.0	209.0	5.3	222	262	285	0	0	248	385
Marri	0.300	140.1	198.0	207.0	1.7	222	262	286	0	0	248	386
Marri	0.370	135.0	189.0	238.0	358.0	222	248	262	340	0	261	495
Marri	0.395	139.2	191.0	212.0	344.0	222	248	262	340	0	258	505
Marri	0.430	137.9	190.0	211.0	1.4	222	248	289	0	0	258	385
Marri	0.475	139.0	192.0	204.0	2.4	226	253	257	0	0	256	387

Legend: **Rf1** – retention factor in **MPB**, **H° DEV 254 nm** – hue and colour equivalent at 254 nm prior to derivatisation, **H° DEV 366 nm** – hue and colour equivalent at 366 nm prior to derivatisation, **H° VSA 366 nm** – hue and colour equivalent at 366 nm after derivatisation w/ VSA derivatisation reagent, **H° T VSA** – hue and colour equivalent at transmittance in white light after derivatisation w/ VSA derivatisation reagent; **Fl DEV λ max**– fluorescence λ max prior to derivatisation, **Fl DEV λ m**– fluorescence λ min prior to derivatisation, **UV DEV λ_{1-3}** – UV-Vis λ max prior to derivatisation, **Fl VS λ**– fluorescence λ max after derivatisation with VSA reagent, **UV VS λ**— UV-Vis λ max after derivatisation with VSA reagent

Supplementary Table S13: Summary of the data used to determine the identity of the unknown bands in *Corymbia calophylla* (Marri) honey (Database 2A)

Name and Code	Rf 2	H° DEV 254 nm	H° DEV 366 nm	H° NP 366 nm	Fl DEV λ	Fl DEV λ m	UV DEV λ_1	UV DEV λ_2	UV DEV λ_3	Fl NP λ	UV NP λ1	UV NP λ2
Marri	0.050	140.0	197.5	176.9	220	255	293	0	0	237	293	0
Marri	0.080	140.7	199.5	183.7	209	257	293	0	0	236	293	0
Marri	0.090	139.8	198.9	184.8	213	257	293	0	0	236	293	0
Marri	0.110	140.0	194.5	186.8	213	257	293	0	0	242	293	0
Marri	0.120	140.5	194.5	187.8	213	247	292	0	0	242	261	291
Marri	0.150	139.9	192.1	189.7	213	247	291	0	0	242	261	291
Marri	0.185	139.3	190.4	193.9	213	254	286	0	0	242	261	291
Marri	0.240	139.8	187.6	195.0	214	252	259	283	0	242	261	291
Marri	0.275	139.9	192.1	191.5	222	262	286	0	0	242	295	0
Marri	0.295	138.6	194.4	117.5	222	262	286	0	0	239	295	0
Marri	0.355	134.7	188.9	165.5	222	248	295	0	0	233	261	291
Marri	0.380	138.4	192.6	103.3	222	248	268	352	0	233	261	291
Marri	0.415	137.7	189.5	180.0	222	248	289	0	0	237	261	291
Marri	0.420	137.5	187.4	187.3	222	248	289	0	0	233	261	291
Marri	0.470	139.3	197.2	196.0	226	253	259	0	0	242	261	288

Legend: **Rf2** – retention factor in MPA, **H° DEV 254 nm** – hue equivalent at 254 nm prior to derivatisation, **H° DEV 366 nm** – hue equivalent at 366 nm prior to derivatisation, **H° NP 366 nm** – hue equivalent at 366 nm after derivatisation w/ NP-PEG derivatisation reagent, **Fl DEV λ**– fluorescence λ max prior to derivatisation, **Fl DEV λ m** – fluorescence λ min prior to derivatisation, **UV DEV $\lambda_{1\text{-}3}$** – UV-Vis λ max prior to derivatisation, **Fl NP λ**– fluorescence λ max after derivatisation with NP-PEG reagent, **UV NP $\lambda_{1\text{-}3}$**– UV-Vis λ max after derivatisation with NP-PEG reagent

Supplementary Table S14: Summary of the data used to determine the identity of the unknown bands in *Corymbia calophylla* (Marri) honey (Database 2B)

Name and Code	Rf 2	H° DEV 254 nm	H° DEV 366 nm	H° VSA 366 nm	H° T VSA	Fl DEV λ	Fl DEV λ m	UV DEV λ1	UV DEV λ2	UV DEV λ3	Fl VS λ	UV VS λ
Marri	0.055	139.7	197.0	204.3	12.0	220	255	289	0	0	258	385
Marri	0.080	140.3	196.0	201.3	14.0	209	257	289	0	0	250	384
Marri	0.095	140.3	196.0	201.7	14.0	213	257	289	0	0	248	385
Marri	0.115	139.5	193.0	201.6	12.0	213	257	289	0	0	245	387
Marri	0.135	139.9	195.0	201.3	13.0	213	247	289	0	0	248	386
Marri	0.145	139.3	194.0	200.5	15.0	213	247	288	0	0	248	388
Marri	0.165	139.9	191.0	203.8	8.9	213	254	288	0	0	247	386
Marri	0.195	140	188.0	211.0	7.8	213	252	288	0	0	254	385
Marri	0.245	139.1	182.0	207.8	16.0	214	252	256	0	0	250	460
Marri	0.285	140.3	192.0	209.3	5.3	222	262	285	0	0	248	385
Marri	0.300	140.1	198.0	207.1	1.7	222	262	286	0	0	248	386
Marri	0.370	135.0	189.0	237.7	358.0	222	248	262	340	0	261	495
Marri	0.395	139.2	191.0	212.1	344.0	222	248	262	340	0	258	383
Marri	0.430	137.9	190.0	210.8	1.4	222	248	289	0	0	258	385
Marri	0.475	139.0	192.0	203.7	2.4	226	253	257	0	0	256	387

Legend: **Rf2** – retention factor in **MPB**, **H° DEV 254 nm** – hue and colour equivalent at 254 nm prior to derivatisation, **H° DEV 366 nm** – hue and colour equivalent at 366 nm prior to derivatisation, **H° VSA 366 nm** – hue and colour equivalent at 366 nm after derivatisation w/ VSA derivatisation reagent, **H° T VSA** – hue and colour equivalent at transmittance in white light after derivatisation w/ VSA derivatisation reagent; **Fl DEV λ max**– fluorescence λ max prior to derivatisation, **Fl DEV λ m**– fluorescence λ min prior to derivatisation, **UV DEV λ_{1-3}** – UV-Vis λ max prior to derivatisation, **Fl VS λ**– fluorescence λ max after derivatisation with VSA reagent, **UV VS λ**— UV-Vis λ max after derivatisation with VSA reagent

Supplementary Table S15: Summary of the data used to determine the identity of the unknown bands in *Eucalyptus marginata* (Jarrah) honey (Database 1A)

Name and Code	Rf 1	H° DEV 254 nm	H° DEV 366 nm	H° NP 366 nm	Fl DEV λ	Fl DEV λ m	UV DEV λ_1	UV DEV λ_2	UV DEV λ_3	Fl NP λ	UV NP λ1	UV NP λ2
Jarrah	0.015	138.0	195.0	190.0	223	251	284			247	298	
Jarrah	0.043	139.0	197.0	194.0	223	252	290			250	298	
Jarrah	0.072	139.0	193.0	194.0	223	255	275			248	298	
Jarrah	0.117	139.0	192.0	195.0	222	252	286			250	295	
Jarrah	0.139	139.0	176.0	194.0	223	252	281			250	301	
Jarrah	0.196	139.0	194.0	194.0	223	252	291			250	317	
Jarrah	0.241	139.0	193.0	195.0	222	252	281			250	316	
Jarrah	0.320	138.0	192.0	199.0	222	252	247			250	296	
Jarrah	0.371	138.0	192.0	197.0	221	252	286			248	293	
Jarrah	0.398	138.0	193.0	198.0	221	248	281			250	294	
Jarrah	0.471	144.0	191.0	198.0	222	250	261	358		246	297	
Jarrah	0.487	140.0	192.0	198.0	221	248	286			245	298	393
Jarrah	0.538	137.0	196.0	194.0	223	248	288			245	297	393
Jarrah	0.562	136.0	196.0	195.0	222	248	288			245	295	
Jarrah	0.633	139.0	202.0	193.0	225	252	259			246	294	

Legend: **Rf1** – retention factor in MPA, **H° DEV 254 nm** – hue equivalent at 254 nm prior to derivatisation, **H° DEV 366 nm** – hue equivalent at 366 nm prior to derivatisation, **H° NP 366 nm** – hue equivalent at 366 nm after derivatisation w/ NP-PEG derivatisation reagent, **Fl DEV λ**– fluorescence λ max prior to derivatisation, **Fl DEV λ m** – fluorescence λ min prior to derivatisation, **UV DEV $\lambda_{1\text{-}3}$** – UV-Vis λ max prior to derivatisation, **Fl NP λ**– fluorescence λ max after derivatisation with NP-PEG reagent, **UV NP $\lambda_{1\text{-}3}$**– UV-Vis λ max after derivatisation with NP-PEG reagent

Supplementary Table S16: Summary of the data used to determine the identity of the unknown bands in *Eucalyptus marginata* (Jarrah) honey (Database 1B)

Name and Code	Rf 1	H° DEV 254 nm	H° DEV 366 nm	H° VSA 366 nm	H° T VSA	Fl DEV λ	Fl DEV λ m	UV DEV λ_1	UV DEV λ_2	UV DEV λ_3	Fl VS λ	UV VS λ
Jarrah	0.700	138.0	160.0	210.0	5.0	223	238	292	0	0	250	367
Jarrah	0.015	138.0	195.0	210.0	20.0	223	251	284	0	0	254	392
Jarrah	0.043	139.0	197.0	219.0	23.0	223	252	290	0	0	250	380
Jarrah	0.072	139.0	193.0	211.0	21.0	223	255	275	0	0	250	379
Jarrah	0.117	139.0	192.0	208.0	20.0	222	252	286	0	0	249	380
Jarrah	0.139	139.0	176.0	210.0	18.0	223	252	281	0	0	250	383
Jarrah	0.196	139.0	194.0	203.0	18.0	223	252	291	0	0	249	379
Jarrah	0.241	139.0	193.0	204.0	19.0	222	252	281	0	0	249	383
Jarrah	0.320	138.0	192.0	210.0	22.0	222	252	247	0	0	249	383
Jarrah	0.371	138.0	192.0	211.0	16.0	221	252	286	0	0	250	380
Jarrah	0.398	138.0	193.0	212.0	17.0	221	248	281		0	250	383
Jarrah	0.471	144.0	191.0	215.0	4.0	222	250	261	358	0	250	380
Jarrah	0.487	140.0	192.0	216.0	11.0	221	248	286	0	0	250	386
Jarrah	0.538	137.0	196.0	228.0	11.0	223	248	288	0	0	252	379
Jarrah	0.562	136.0	196.0	231.0	340.0	222	248	288	0	0	252	380
Jarrah	0.633	139.0	202.0	209.0	12.0	225	252	259	0	0	249	369

Legend: **Rf1** – retention factor in **MPB**, **H° DEV 254 nm** – hue and colour equivalent at 254 nm prior to derivatisation, **H° DEV 366 nm** – hue and colour equivalent at 366 nm prior to derivatisation, **H° VSA 366 nm** – hue and colour equivalent at 366 nm after derivatisation w/ VSA derivatisation reagent, **H° T VSA** – hue and colour equivalent at transmittance in white light after derivatisation w/ VSA derivatisation reagent; **Fl DEV λ max**– fluorescence λ max prior to derivatisation, **Fl DEV λ m**– fluorescence λ min prior to derivatisation, **UV DEV λ_{1-3}** – UV-Vis λ max prior to derivatisation, **Fl VS λ**– fluorescence λ max after derivatisation with VSA reagent, **UV VS λ**— UV-Vis λ max after derivatisation with VSA reagent

Supplementary Table S17: Summary of the data used to determine the identity of the unknown bands in *Eucalyptus marginata* (Jarrah) honey (Database 2A)

Name and Code	Rf 2	H° DEV 254 nm	H° DEV 366 nm	H° NP 366 nm	Fl DEV λ	Fl DEV λ m	UV DEV λ_1	UV DEV λ_2	UV DEV λ_3	Fl NP λ	UV NP λ1	UV NP λ2
Jarrah	0.070	140.9	215.3	193.8	210	249	300	0	0	236	315	0
Jarrah	0.100	140.3	191.2	193.9	222	251	287	0	0	242	305	0
Jarrah	0.140	140.3	195.5	194.1	203	248	287	0	0	237	297	0
Jarrah	0.185	138.9	191.6	196.0	219	255	288	0	0	236	293	0
Jarrah	0.270	144.4	193.0	195.2	223	254	264	358	0	242	301	0
Jarrah	0.290	138.7	193.3	94.2	223	254	286	0	0	239	307	0
Jarrah	0.330	139.7	195.2	102.2	222	255	287	0	0	239	301	0
Jarrah	0.355	136.8	192.6	178.4	222	248	293	0	0	231	297	0
Jarrah	0.385	139.8	195.5	173.2	222	248	297	0	0	242	301	435
Jarrah	0.430	139.1	194.7	184.0	222	248	297	0	0	239	303	0
Jarrah	0.460	140.3	202.9	196.1	228	245	285	0	0	242	295	0
Jarrah	0.475	139.8	199.4	196.1	226	255	258	0	0	239	296	0
Jarrah	0.525	139.9	194.2	195.2	207	251	287	0	0	248	303	0

Legend: **Rf2** – retention factor in MPA, **H° DEV 254 nm** – hue equivalent at 254 nm prior to derivatisation, **H° DEV 366 nm** – hue equivalent at 366 nm prior to derivatisation, **H° NP 366 nm** – hue equivalent at 366 nm after derivatisation w/ NP-PEG derivatisation reagent, **Fl DEV λ**– fluorescence λ max prior to derivatisation, **Fl DEV λ m** – fluorescence λ min prior to derivatisation, **UV DEV $\lambda_{1\text{-}3}$** – UV-Vis λ max prior to derivatisation, **Fl NP λ**– fluorescence λ max after derivatisation with NP-PEG reagent, **UV NP $\lambda_{1\text{-}3}$**– UV-Vis λ max after derivatisation with NP-PEG reagent

Supplementary Table S18: Summary of the data used to determine the identity of the unknown bands in *Eucalyptus marginata* (Jarrah) honey (Database 2B)

Name and Code	Rf 2	H° DEV 254 nm	H° DEV 366 nm	H° VSA 366 nm	H° T VSA	Fl DEV λ	Fl DEV λ m	UV DEV λ_1	UV DEV λ_2	UV DEV λ_3	Fl VS λ	UV VS λ
Jarrah	0.065	140.4	190.3	200.6	0.9	208	252	298	0	0	258	383
Jarrah	0.080	140.4	201.4	200.2	11.4	210	249	299	0	0	258	383
Jarrah	0.105	139.5	188.4	205.0	9.4	222	251	293	0	0	254	386
Jarrah	0.135	139.8	191.6	205.8	12.5	203	248	289	0	0	255	387
Jarrah	0.150	140.0	193.9	207.9	18.8	208	251	288	0	0	255	477
Jarrah	0.195	138.4	189.3	207.6	8.9	219	255	288	0	0	258	387
Jarrah	0.285	143.2	191.8	206.2	14.2	223	254	264	356	0	256	477
Jarrah	0.305	140.6	193.0	204.9	17.8	222	255	286	0	0	258	477
Jarrah	0.345	140.3	192.2	212.6	1.0	222	248	288	0	0	256	477
Jarrah	0.365	140.3	192.2	212.6	1.0	222	248	290	0	0	261	483
Jarrah	0.375	136.5	192.2	232.7	352.9	222	247	293	0	0	261	483
Jarrah	0.395	139.8	193.6	209.6	351.6	222	248	296	0	0	258	384
Jarrah	0.430	138.8	192.2	197.9	3.9	222	248	297	0	0	251	383
Jarrah	0.465	140.7	199.4	206.7	0.0	226	245	282	0	0	250	386
Jarrah	0.48	139.7	199.8	205.4	1.4	226	255	257	0	0	250	387
Jarrah	0.535	139.8	192.7	203.7	4.6	207	252	287	0	0	248	386
Jarrah	0.545	139.8	192.7	203.7	4.6	207	252	251	0	0	250	386

Legend: **Rf2** – retention factor in **MPB**, **H° DEV 254 nm** – hue and colour equivalent at 254 nm prior to derivatisation, **H° DEV 366 nm** – hue and colour equivalent at 366 nm prior to derivatisation, **H° VSA 366 nm** – hue and colour equivalent at 366 nm after derivatisation w/ VSA derivatisation reagent, **H° T VSA** – hue and colour equivalent at transmittance in white light after derivatisation w/ VSA derivatisation reagent; **Fl DEV λ max**– fluorescence λ max prior to derivatisation, **Fl DEV λ m**– fluorescence λ min prior to derivatisation, **UV DEV λ_{1-3}** – UV-Vis λ max prior to derivatisation, **Fl VS λ**– fluorescence λ max after derivatisation with VSA reagent, **UV VS λ**— UV-Vis λ max after derivatisation with VSA reagent

Supplementary Table S19: Parameters used in Optimising the Quantification of Phenolic Compounds in Honey

Parameters		Correlation (R^2)	Accuracy	Regression Mode	Remarks
Concentration (application)	500 µg/mL	<0.99	>±5%	Polynomial	Match compounds were mixed in order to save time and resources
	100 µg/mL	<0.99	>±5%	Polynomial	
	5-50 µg/mL	>0.99	<±5%	Linear	
Volume (application)	5.0-25.0 µL	>0.99	>±5%	Polynomial	Lower volume application was favoured in order to save time in sample injection
	5.0-12.5 µL	>0.99	<±5%	Polynomial	
	5.0 – 9.8 µL	>0.99	<±5%	Linear	
Profile	254 nm	<0.99	>±5%	Polynomial	Better correlations and accuracy were observed when the specific λmax of the standards were used
	366 nm	<0.99	>±5%	Polynomial	
	Specific λ max	>0.99	<±5%	Linear	
Derivatisation	With NP-PEG	>0.99	<±5%	Linear	No derivatisation was chosen in order to save time
	None	>0.99	<±5%	Linear	

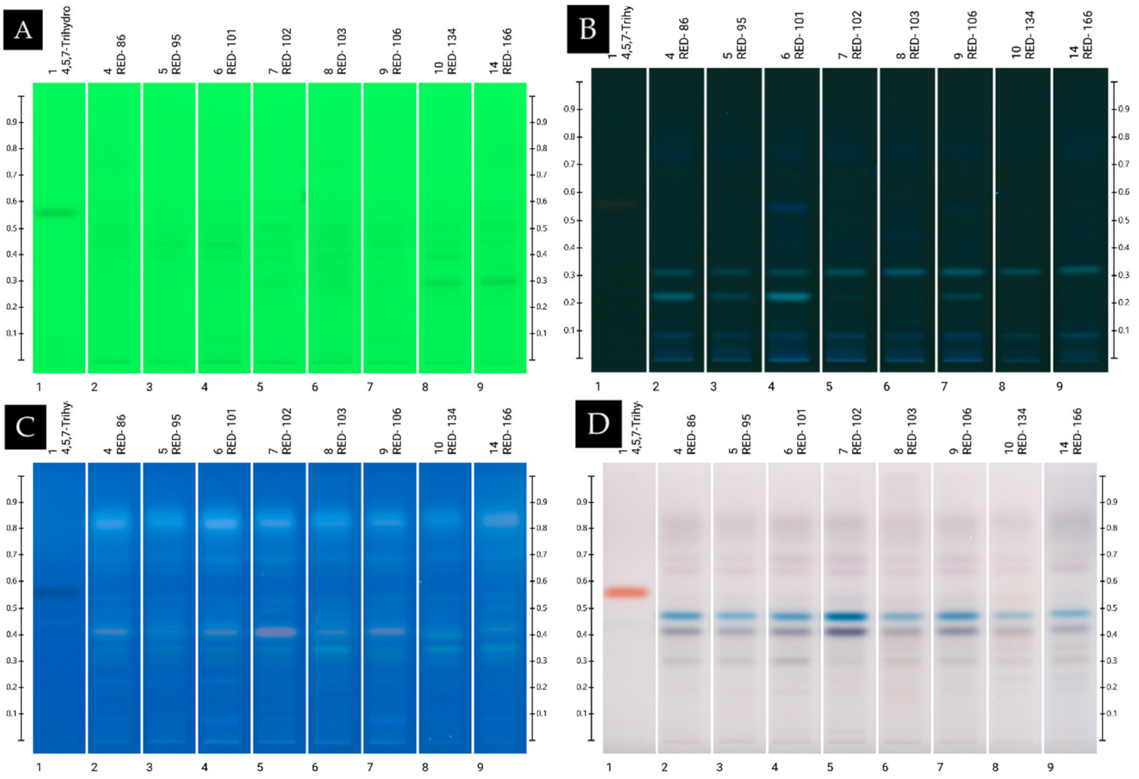

Supplementary Figure S1. HPTLC fingerprint patterns for various samples of *Calothamnus* spp. (Red bell, n=8). Plate images taken and obtained under the following light conditions: 254 nm prior to derivatisation (**A**), 366 nm prior to derivatisation (**B**), 366 nm after derivatised with VSA (**C**), and transmittance in white light after derivatisation with VSA (**D**).

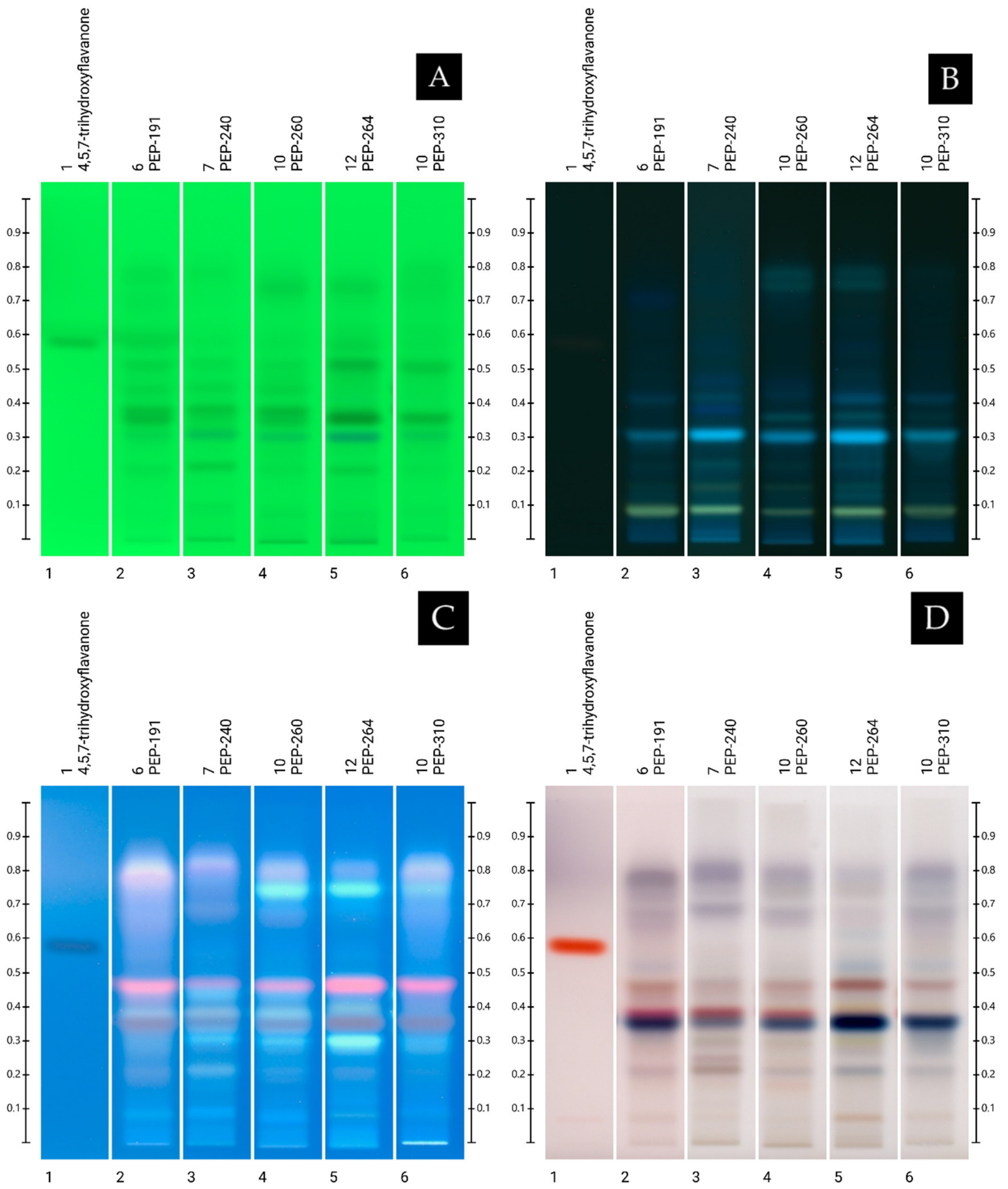

Supplementary Figure S2. HPTLC fingerprint patterns for various samples of *Agonis flexuosa* (Coastal Peppermint, n=5). Plate images taken and obtained under the following light conditions: 254 nm prior to derivatisation (**A**), 366 nm prior to derivatisation (**B**), 366 nm after derivatised with VSA (**C**), and transmittance in white light after derivatisation with VSA (**D**).

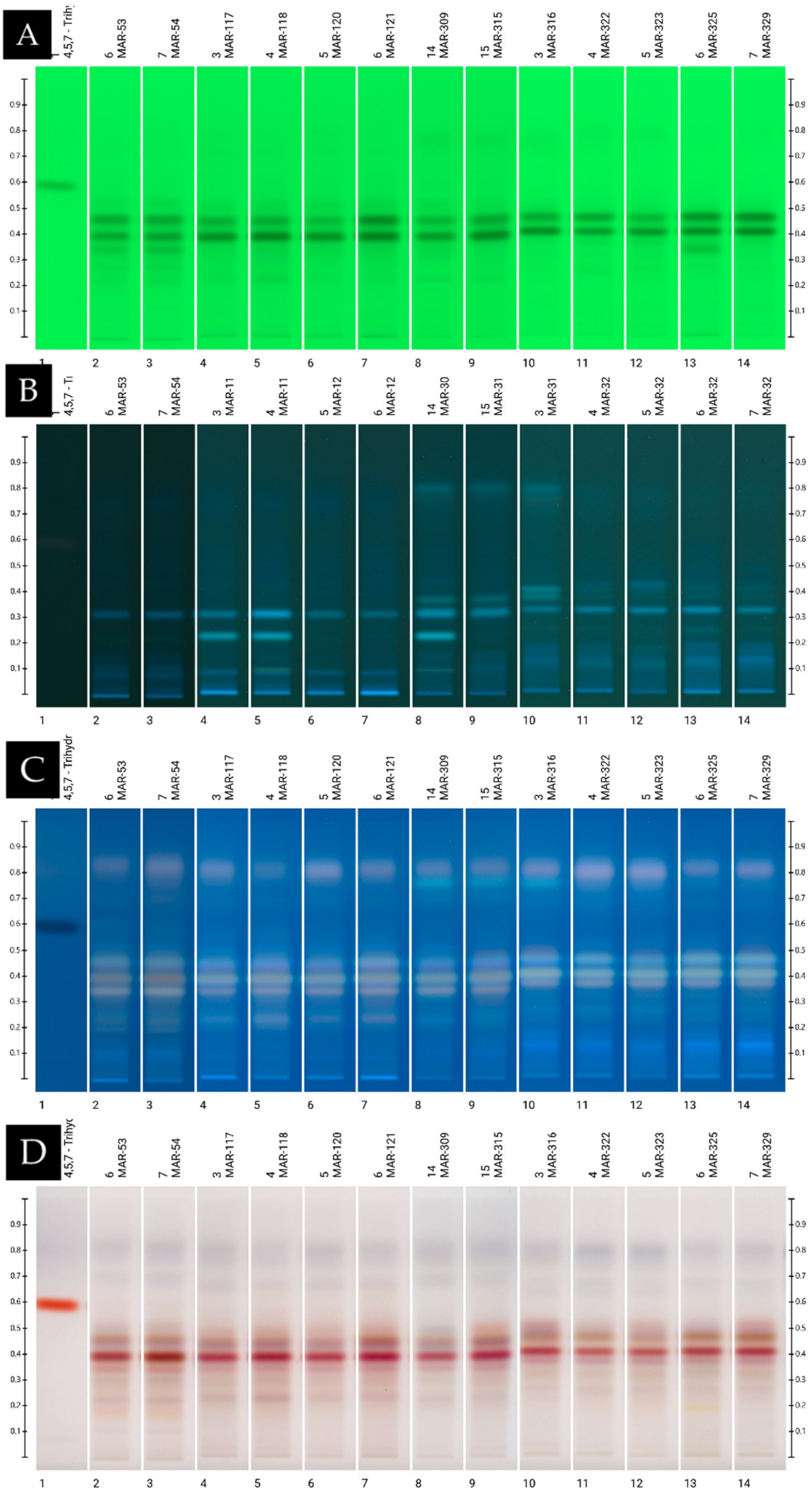

Supplementary Figure S3. HPTLC fingerprint patterns for various samples of *Corymbia calophylla* (Marri, n=13). Plate images taken and obtained under the following light conditions: 254 nm prior to derivatisation (**A**), 366 nm prior to derivatisation (**B**), 366 nm after derivatised with VSA (**C**), and transmittance in white light after derivatisation with VSA (**D**).

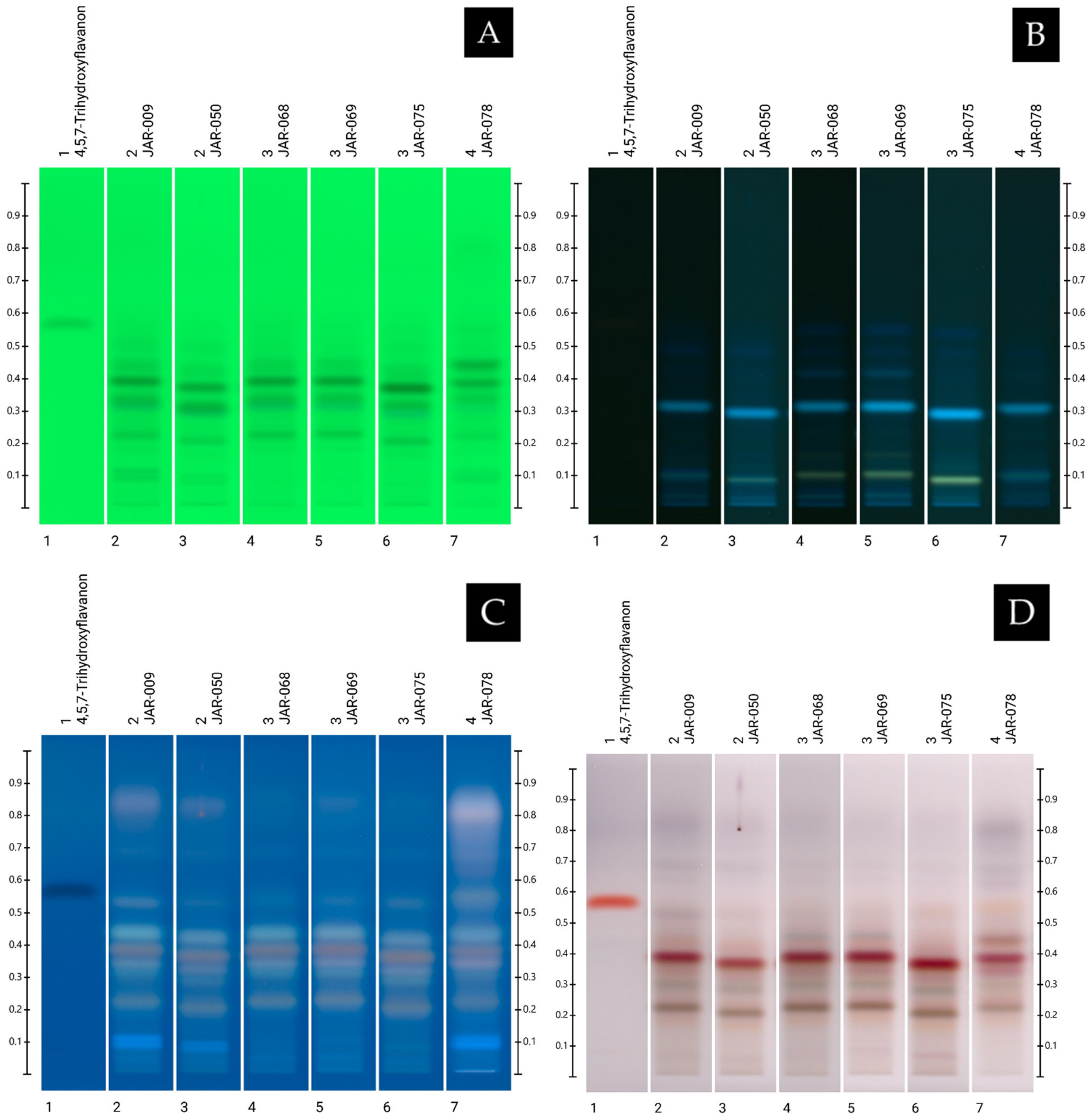

Supplementary Figure S4. HPTLC fingerprint patterns for various samples of *Eucalyptus marginata* (Jarrah, n=6). Plate images taken and obtained under the following light conditions: 254 nm prior to derivatisation (**A**), 366 nm prior to derivatisation (**B**), 366 nm after derivatised with VSA (**C**), and transmittance in white light after derivatisation with VSA (**D**).

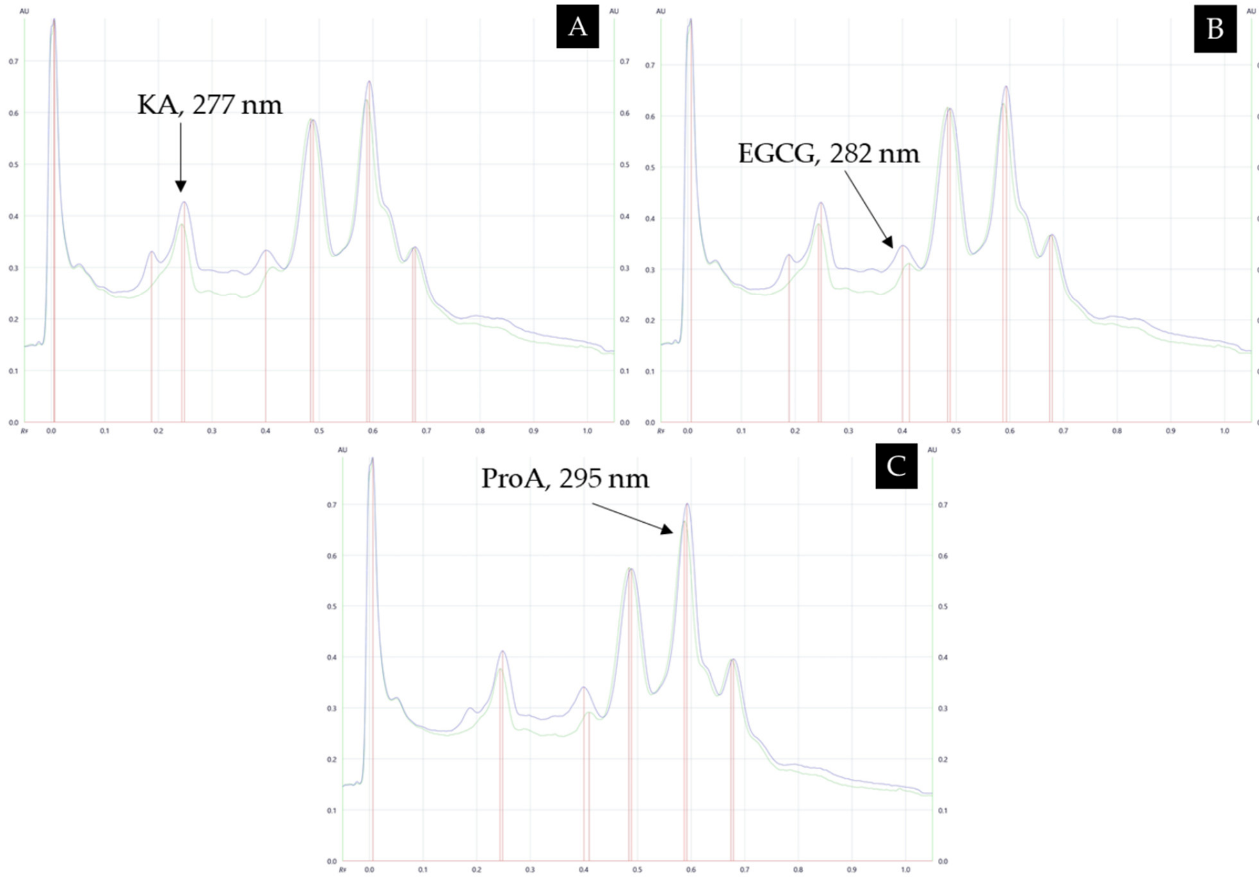

Supplementary Figure S5 (A-C). Profile Comparison of *Calothamnus* spp. (Red bell) Honey (green) and *Calothamnus* spp. (Red bell) Honey Spiked with the Identified Compounds Based on Database 1A and 1B (blue) Scanned at the λmax of Each Specific Compound Prior to Derivatisation

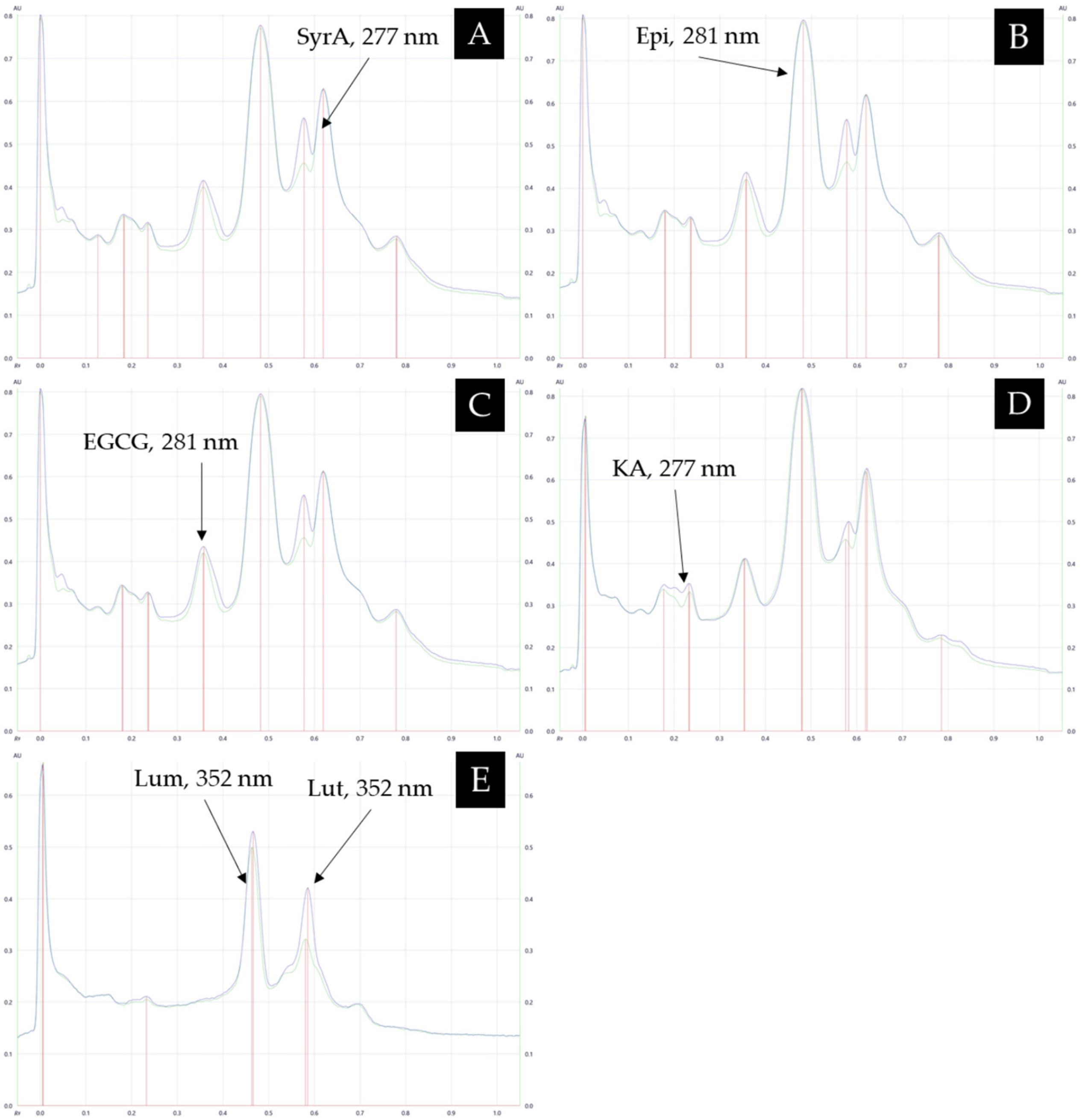

Supplementary Figure S6 (A-E). Profile Comparison of *Agonis flexuosa* (Coastal Peppermint) Honey (green) and *Agonis flexuosa* (Coastal Peppermint) Honey Spiked with the Identified Compounds Based on Database 1A and 1B (blue) Scanned at the λmax of Each Specific Compounds Prior to Cerivatisation

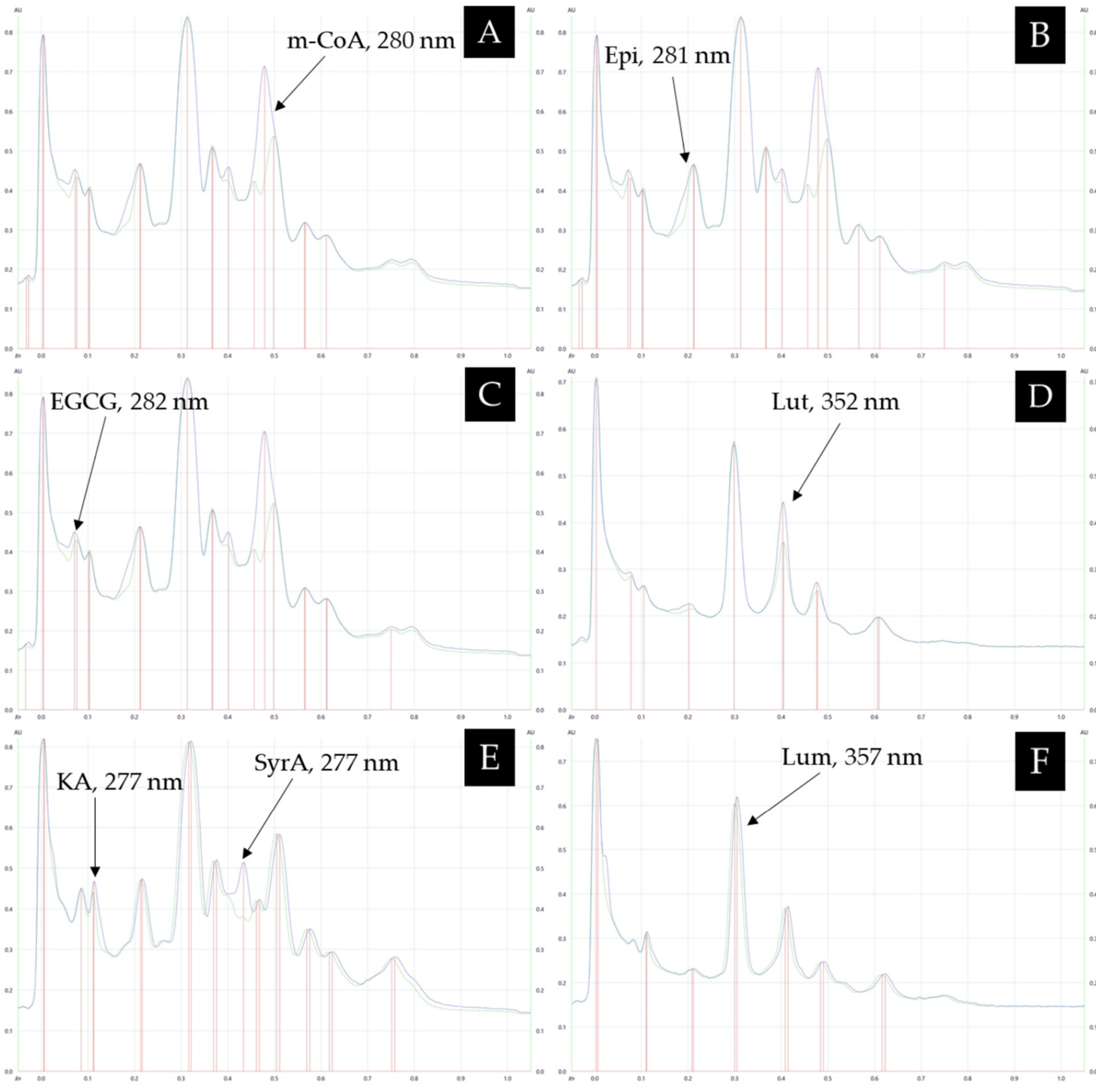

Supplementary Figure S7 (A-F). Profile Comparison of *Agonis flexuosa* (Coastal Peppermint) Honey (green) and *Agonis flexuosa* (Coastal Peppermint) Honey Spiked with the Identified Compounds Based on Database 2A and 2B (blue) Scanned at the λmax of Each Specific Compound Prior to Derivatisation

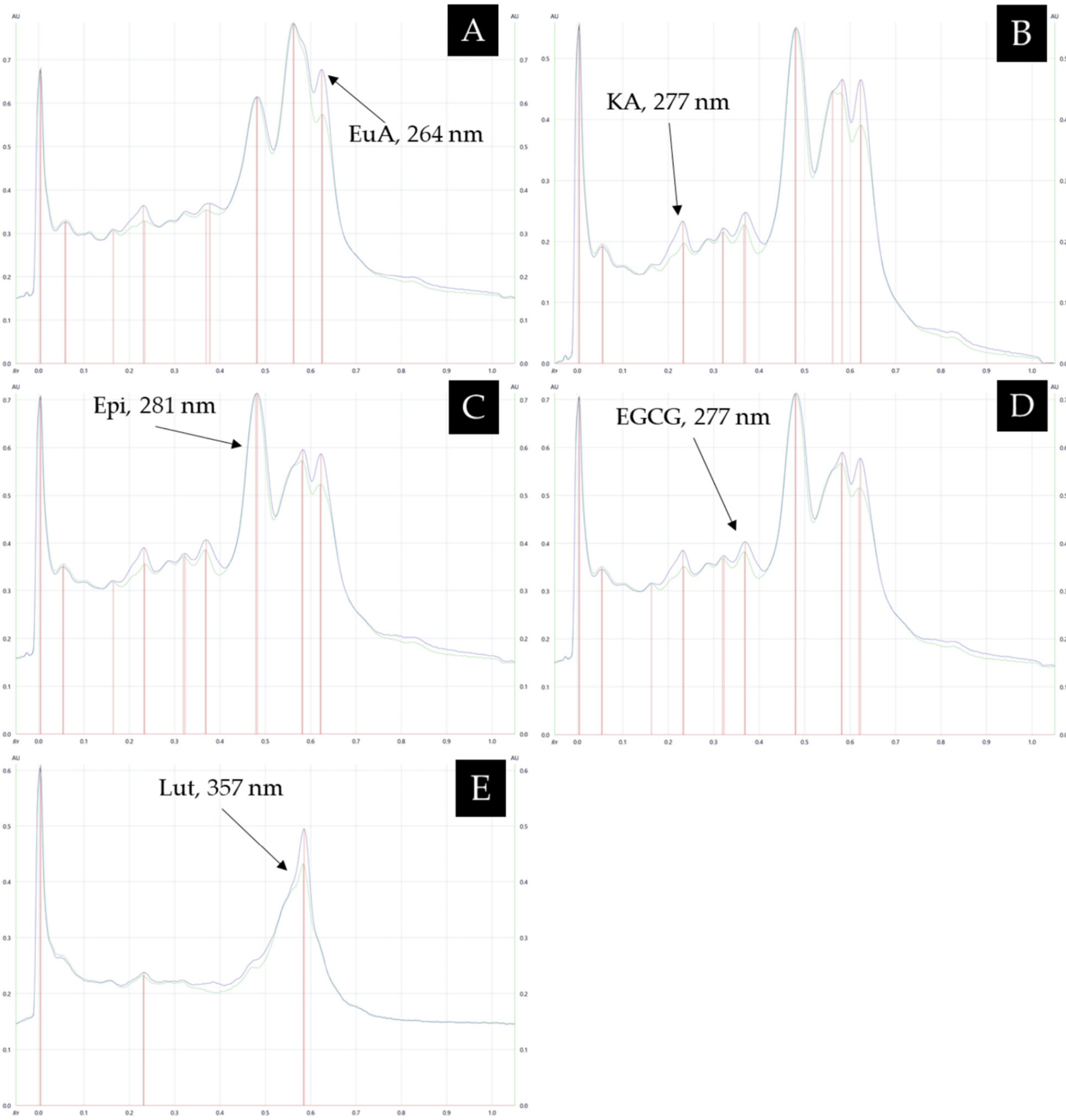

Supplementary Figure S8 (A-E). Profile Comparison of *Corymbia calophylla* (Marri) Honey (green) and *Corymbia calophylla* (Marri) Spiked with the Identified Compounds Based on Database 1A and 1B (blue) scanned at the λmax of Each Specific Compound Prior to Derivatisation

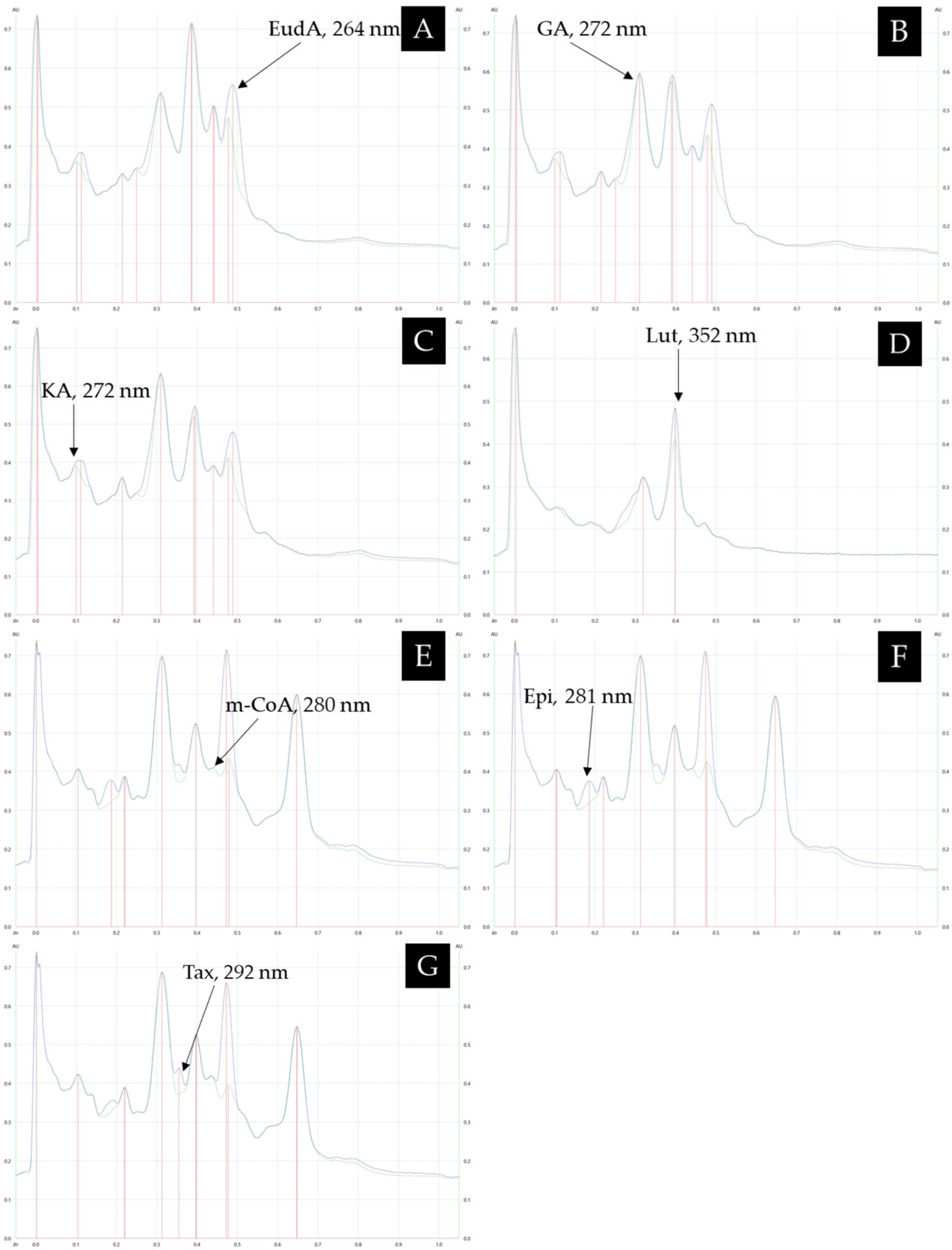

Supplementary Figure S9 (A-G). Profile Comparison of *Corymbia calophylla* (Marri) Honey (green) and *Corymbia calophylla* (Marri) Spiked with the Identified Compounds Based on Database 2A and 2B (blue) Scanned at the λmax of Each Specific Compound Prior to Derivatisation

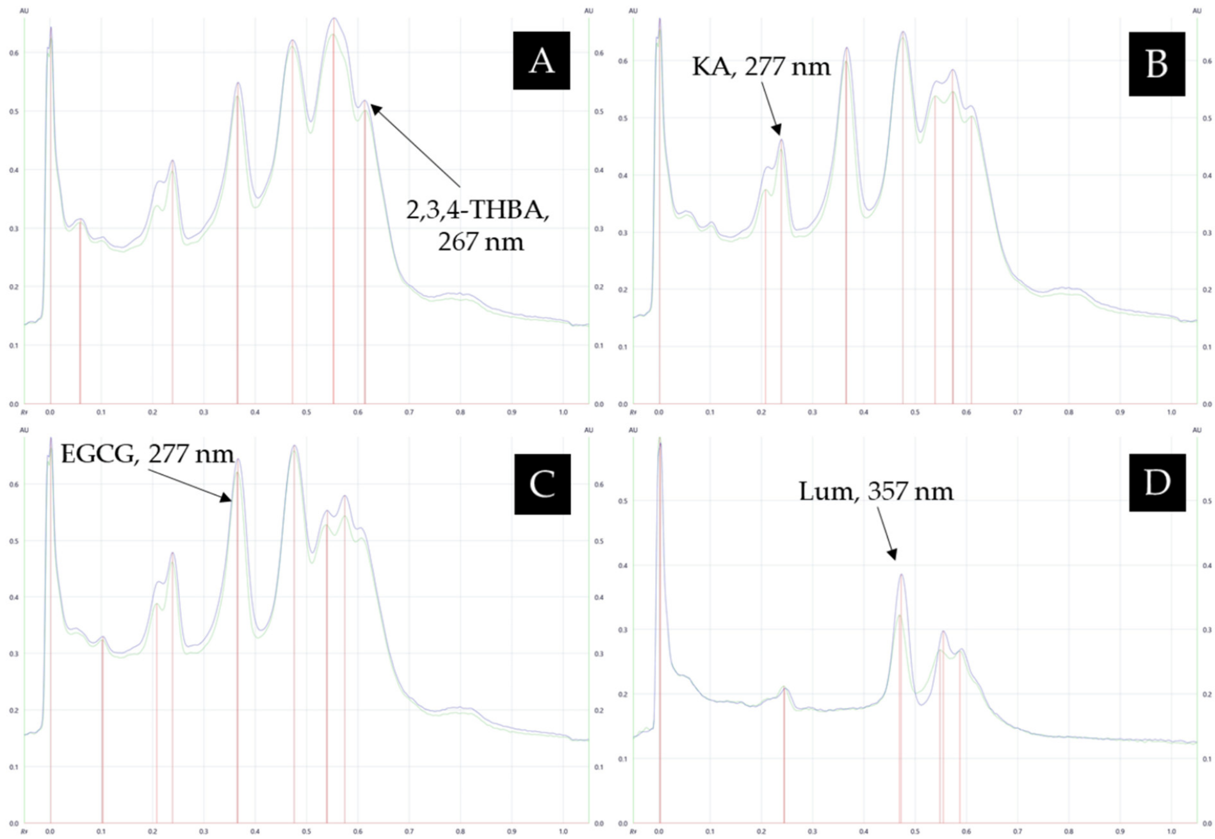

Supplementary Figure S10 (A-D). Profile Comparison of *Eucalyptus marginata* (Jarrah) Honey (green) and *Eucalyptus marginata* (Jarrah) Spiked with the Identified Compounds Based on Database 1A and 1B (blue) Scanned at the λmax of Each Specific Compound Prior to Derivatisation

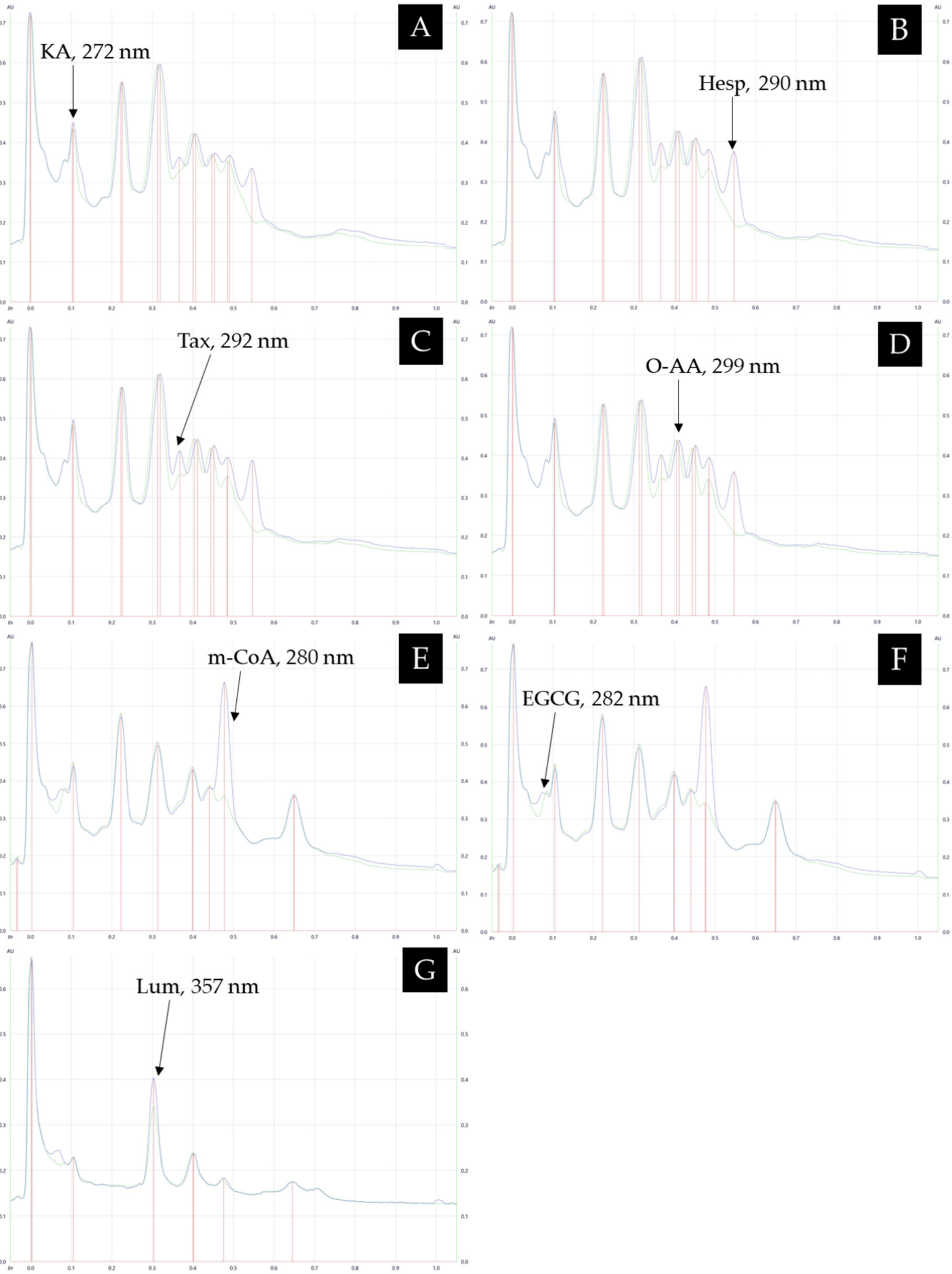

Supplementary Figure S11 (A-G). Profile Comparison of *Eucalyptus marginata* (Jarrah) Honey (green) and *Eucalyptus marginata* (Jarrah) Spiked with the Identified Compounds Based on Database 2A and 2B (blue) Scanned at the λmax of Each Specific Compound Prior to Derivatisation

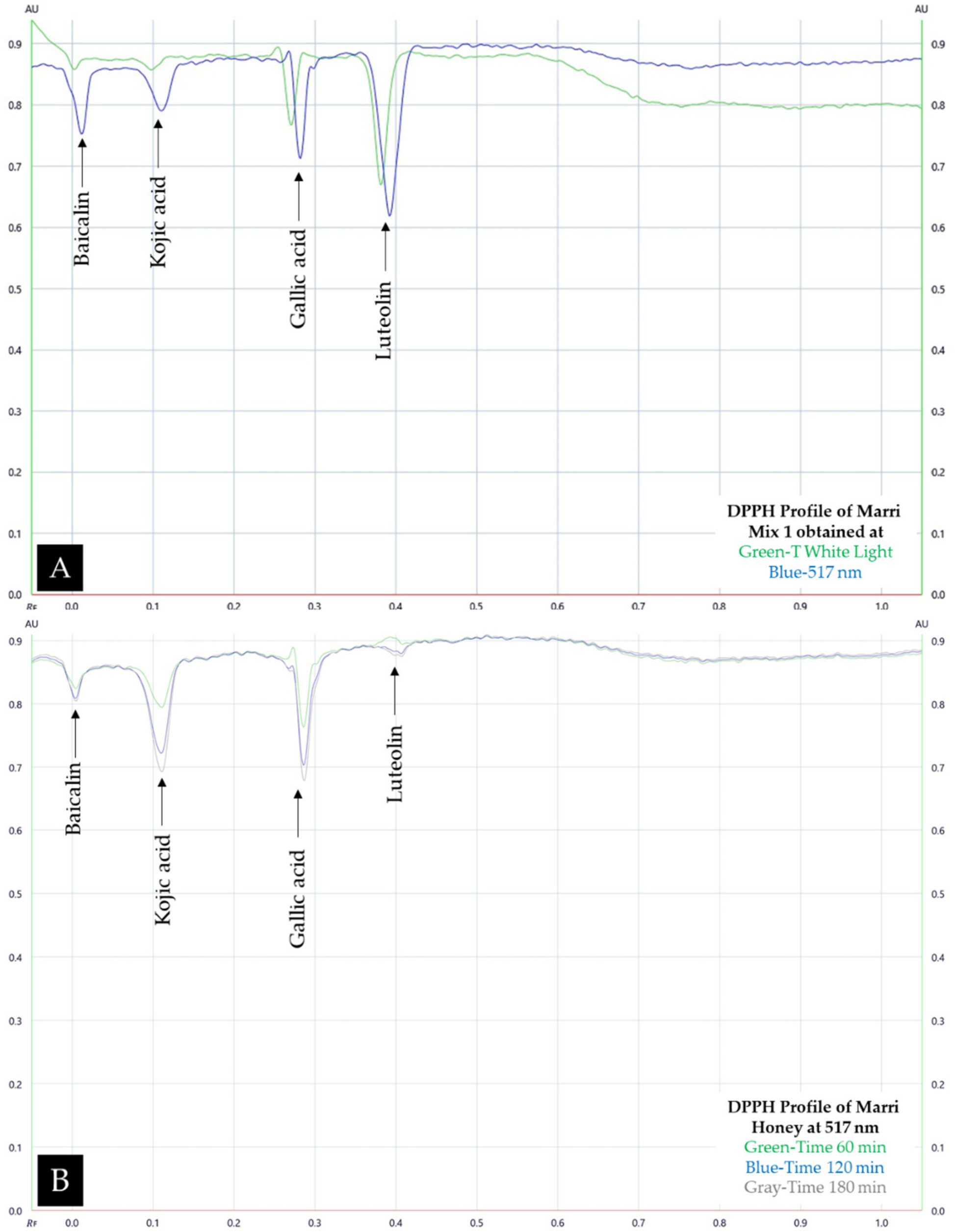

Supplementary Figure S12 (A and B). Comparison of the profiles of compounds identified in *Corymbia calophylla* (Marri*)* honey extract developed using mobile phase 1B after derivatisation with DPPH reagent after 1 h at transmittance in white light (green) vs scanned at 517 nm (**A**) and Comparison of the profiles of compounds identified in *Corymbia calophylla* (Marri*)* honey after derivatisation with DPPH reagent scanned at 517 nm and taken after 1 h (green), 2 h (blue), 3 h (gray) (**B)**.

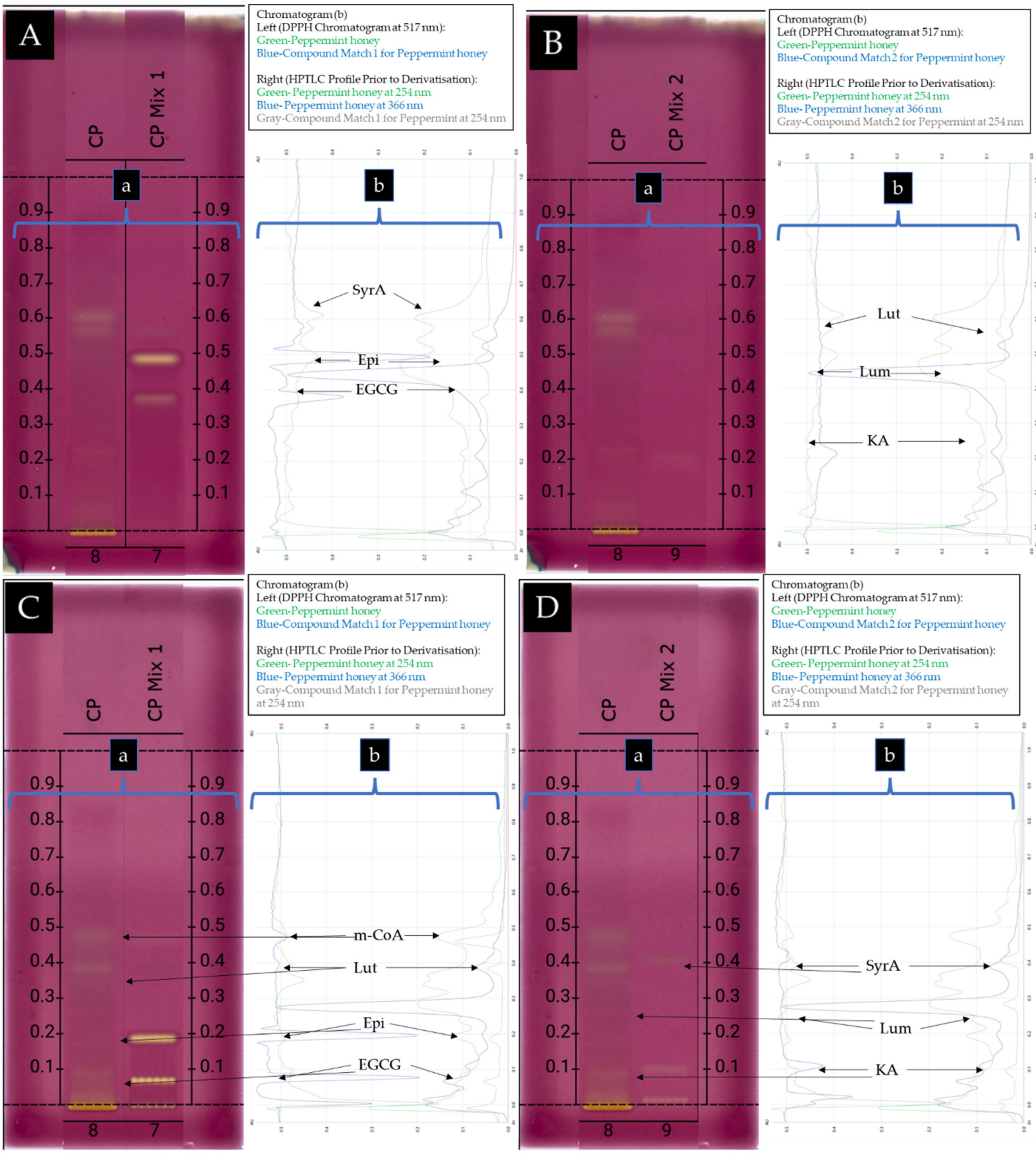

Supplementary Figure S13 (A - D). HPTLC Plate Image (**a**) of *Agonis flexuosa* (Coastal Peppermint) honey after derivatisation with DPPH reagent and developed using MPA (**A and B**) and developed using MPB (**C and D**) obtained at transmission in white light, and comparison of the profiles of *Agonis flexuosa* (Coastal Peppermint) honey (green) and *Agonis flexuosa* (Coastal Peppermint) honey spiked with the identified compounds (blue) after derivatisation with DPPH reagent obtained at 517 nm (**b-left)** and comparison of the profiles of *Agonis flexuosa* (Coastal Peppermint) honey obtained at 254 nm (green) and 366 nm (blue) prior to derivatisation and the profile of *Agonis flexuosa* (Coastal Peppermint) honey spiked with the identified compounds (gray) obtained at 277 nm prior to derivatisation (**b right)**

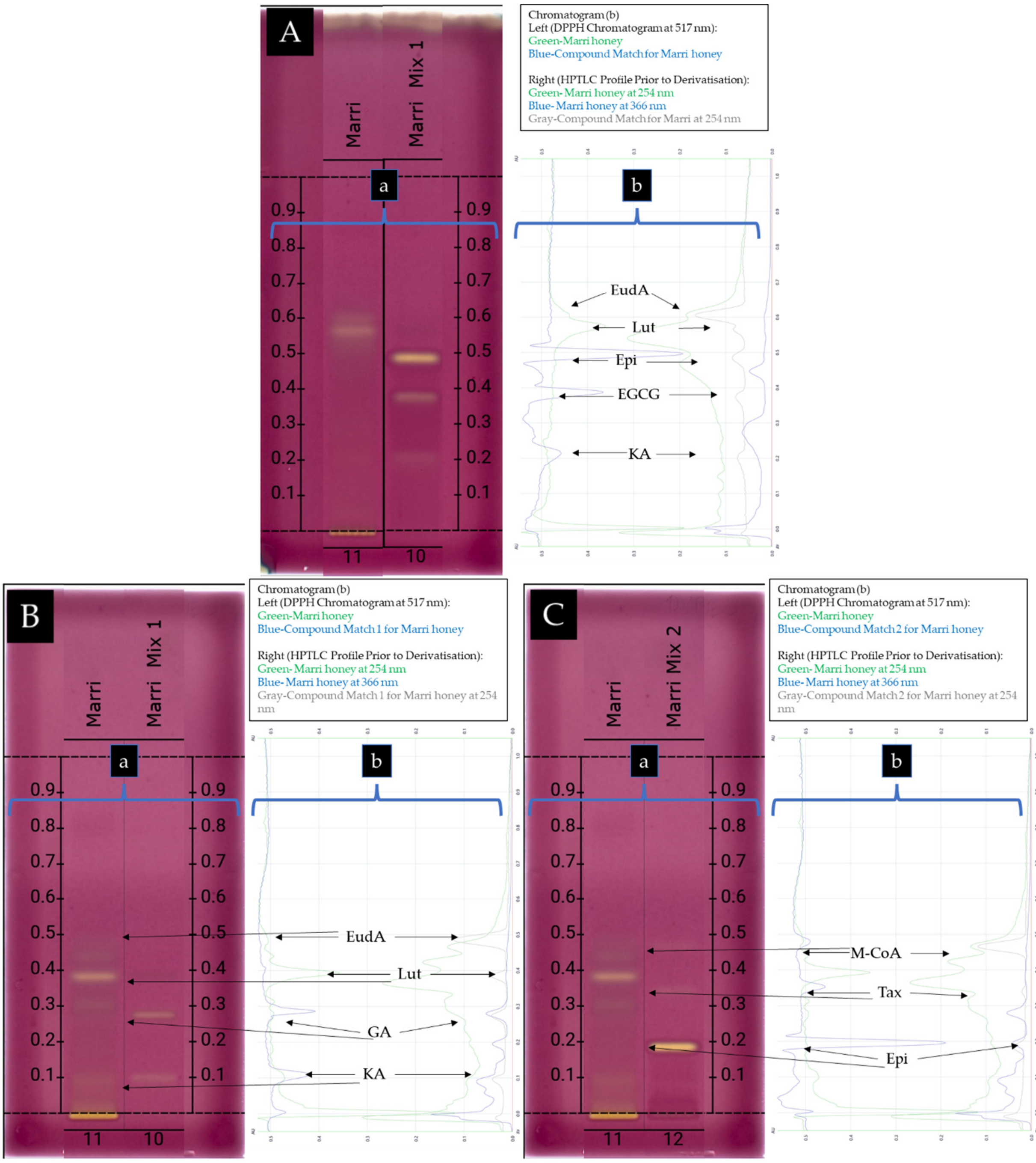

Supplementary Figure S14. (A - C). HPTLC Plate Image (**a**) of *Corymbia calophylla* (Marri) honey after derivatisation with DPPH reagent and developed using MPA (**A**) and developed using MPB (**B and C**) obtained at transmission in white light, and comparison of the profiles of *Corymbia calophylla* (Marri) honey (green) and *Corymbia calophylla* (Marri) honey spiked with the identified compounds (blue) after derivatisation with DPPH reagent obtained at 517 nm (**b-left)** and comparison of the profiles of *Corymbia calophylla* (Marri) honey obtained at 254 nm (green) and 366 nm (blue) prior to derivatisation and the profile of *Corymbia calophylla* (Marri) honey spiked with the identified compounds (gray) obtained at 277 nm prior to derivatisation (**b right)**

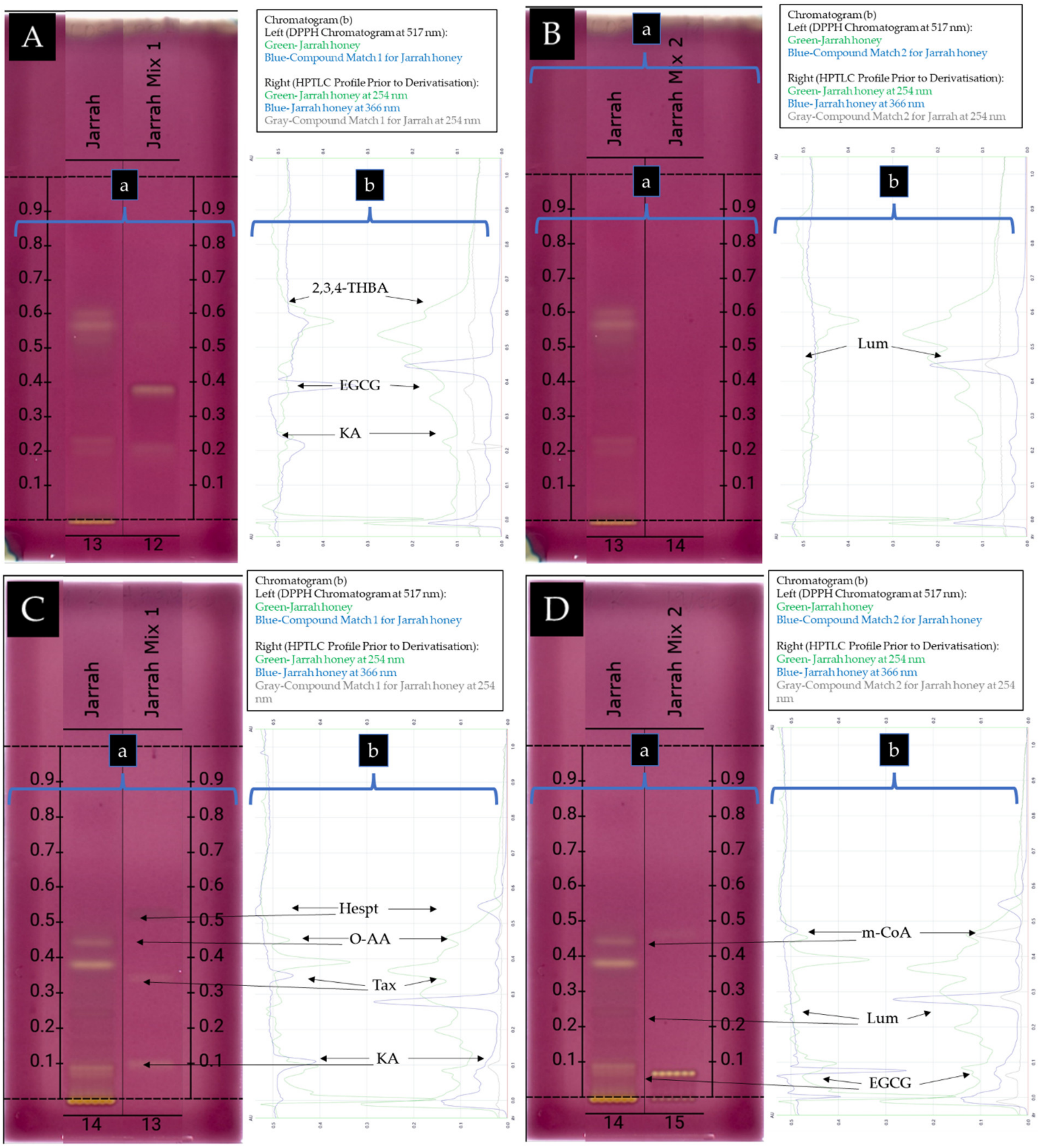

Supplementary Figure S15. (A - D). HPTLC Plate Image (**a**) of *Eucalyptus marginata* (Jarrah) honey after derivatisation with DPPH reagent and developed using MPA (**A and B**) and developed using MPB (**C and D**) obtained at transmission in white light, and comparison of the profiles of *Eucalyptus marginata* (Jarrah) honey (green) and Jarrah honey spiked with the identified compounds (blue) after derivatisation with DPPH reagent obtained at 517 nm (**b-left)** and comparison of the profiles of *Eucalyptus marginata* (Jarrah) honey obtained at 254 nm (green) and 366 nm (blue) prior to derivatisation and the profile of *Eucalyptus marginata* (Jarrah) honey spiked with the identified compounds (gray) obtained at 277 nm prior to derivatisation (**b right)**
